# FRUITFULL controls *SAUR10* expression and regulates Arabidopsis growth and architecture

**DOI:** 10.1093/jxb/erx184

**Published:** 2017-06-06

**Authors:** Marian Bemer, Hilda van Mourik, Jose M Muiño, Cristina Ferrándiz, Kerstin Kaufmann, Gerco C Angenent

**Affiliations:** 1 Laboratory of Molecular Biology, Wageningen University & Research, Droevendaalsesteeg, PB Wageningen, The Netherlands; 2 Department of Biology, Institute of Biology, Humboldt University, Berlin, Germany; 3 Instituto de Biología Molecular y Celular de Plantas (IBMCP),Universidad Politécnica de Valencia (UPV)-Consejo Superior de Investigaciones Científicas (CSIC). Ciudad Politécnica de la Innovación (CPI), Ed. 8E C/ Ingeniero Fausto Elio s/n, Valencia, Spain; 4 Institute for Biochemistry and Biology, Potsdam University, Potsdam, Germany; 5 Laboratory of Molecular Biology and Business Unit Bioscience, Wageningen University & Research, PB Wageningen, The Netherlands

**Keywords:** Architecture, auxin, branching, FRUITFULL, growth, hormones, light response, MADS-box transcription factor, SAUR

## Abstract

MADS-domain transcription factors are well known for their roles in plant development and regulate sets of downstream genes that have been uncovered by high-throughput analyses. A considerable number of these targets are predicted to function in hormone responses or responses to environmental stimuli, suggesting that there is a close link between developmental and environmental regulators of plant growth and development. Here, we show that the Arabidopsis MADS-domain factor FRUITFULL (FUL) executes several functions in addition to its noted role in fruit development. Among the direct targets of FUL, we identified *SMALL AUXIN UPREGULATED RNA 10* (*SAUR10*), a growth regulator that is highly induced by a combination of auxin and brassinosteroids and in response to reduced R:FR light. Interestingly, we discovered that *SAUR10* is repressed by FUL in stems and inflorescence branches. *SAUR10* is specifically expressed at the abaxial side of these branches and this localized activity is influenced by hormones, light conditions and by FUL, which has an effect on branch angle. Furthermore, we identified a number of other genes involved in hormone pathways and light signalling as direct targets of FUL in the stem, demonstrating a connection between developmentally and environmentally regulated growth programs.

## Teaser

The MADS-domain factor FRUITFULL functions in stem growth and plant architecture by directly regulating a number of signalling genes, including the hormone and shade-induced growth regulator *SAUR10*.

## Introduction

Plant growth and development are regulated by interplay between internal and external factors. The timely expression of different sets of transcription factors regulates the default program of plant growth and development, but this program is highly influenced by external factors that allow the plant to adapt its growth according to the environmental conditions. As a result, plants with the same genotype show distinct phenotypic differences when, for example, grown at different temperatures or under different light conditions. This response to environmental conditions is mainly regulated via hormonal pathways and involves auxin, gibberellic acid (GA), cytokinin and brassinosteroids (BRs). In particular auxin, which induces cell elongation, has been shown to be essential for growth responses to environmental conditions such as phototropism and gravitropism (Paponov *et al.*, 2008; Fankhauser and Christie, 2015). Recently, the light-regulated growth of Arabidopsis hypocotyls has been thoroughly investigated and revealed to depend on physical interactions between transcription factors involved in auxin, GA, BR and light responses (Bai *et al.*, 2012; Oh *et al.*, 2014; Ross and Quittenden, 2016). Downstream growth-regulating genes can be induced or repressed by either the hormone-mediated environmental response pathway or by the internal developmental pathway, thus integrating these two pathways in the growth response.

A group of growth regulators that has been shown to be highly responsive to auxin and other hormonal stimuli is the SAUR family of Small Auxin-Upregulated RNAs. *SAUR* transcripts were first discovered in soybean and found to be rapidly upregulated after addition of auxin. Additional research in soybean and other species has revealed that SAUR activity is highly dynamic, as both transcript and protein half-lives were reported to be extremely short (McClure and Guilfoyle, 1987; Knauss *et al.*, 2003). In Arabidopsis, the *SAUR* gene family contains 79 genes (Ren and Gray, 2015), of which approximately two-thirds have been found to respond to auxin in certain tissues (Paponov *et al.*, 2008; Chapman *et al.*, 2012; Bargmann *et al.*, 2013). In addition, several *SAUR* genes have been found to be influenced by other hormones like abscisic acid (ABA), ethylene, GA and BRs (Kodaira *et al.*, 2011; Walcher and Nemhauser, 2012; Stamm and Kumar, 2013; Oh *et al.*, 2014; Li *et al.*, 2015). The function of several Arabidopsis *SAUR* genes has been investigated using overexpression studies, unveiling their general capacity to promote cell elongation in growth-related processes (Chae *et al.*, 2012; Spartz *et al.*, 2012; Stamm and Kumar, 2013; Ren and Gray, 2015; Sun *et al.*, 2016). For a long time, it was unknown how induced *SAUR* gene expression could result in increased cell elongation, but a study by Spartz *et al.* (2014) recently unveiled that SAURs act according to the earlier postulated acid growth theory (Rayle and Cleland, 1992). They interact with protein phosphatases of the PP2C-D family to inhibit their function, thereby preventing dephosphorylation of plasma membrane H^+^-ATPases, resulting in activation of these membrane pumps. Activation of the H^+^-ATPases leads to membrane acidification, which enables cell elongation. Different Arabidopsis SAURs were tested and they were all able to interact with PP2C-Ds (Spartz *et al.*, 2014; Sun *et al.*, 2016).

In addition to being responsive to hormones, *SAUR* genes have also been reported as targets of several transcription factors involved in plant development, such as the MADS domain transcription factors SEPALLATA3 (SEP3) and APETALA1 (AP1), and the TCP (TEOSINTE BRANCHED1/CYCLOIDEA/ PROLIFERATING CELL FACTOR1) family protein TCP20 (Kaufmann *et al.*, 2009; Kaufmann *et al.*, 2010*b*; Danisman *et al.*, 2012), suggesting that growth regulators of the SAUR family can act as integrators of the developmental and environmental growth pathways. However, the interaction between both pathways during plant growth is poorly understood.

We performed a ChIP-seq experiment with the Arabidopsis MADS-domain factor FRUITFULL (FUL) and identified two closely related *SAUR* genes, *SAUR10* and *SAUR16*, as strongly bound target genes. FUL is a major player in the network that regulates Arabidopsis fruit development and determines both fruit patterning and growth (Gu *et al.*, 1998; Ferrándiz *et al.*, 2000*b*). In addition, *ful* mutants were also reported to flower later than wild-type (Ferrándiz *et al.*, 2000*a*) and to exhibit an altered cauline leaf shape (Gu *et al.*, 1998). Here, we demonstrate that FUL plays additional and novel roles in plant growth and is able to directly regulate genes involved in hormone- and light-induced cell elongation, such as the DELLA genes *RGL2* and *GAI*, *PHYTOCHROME INTERACTING FACTOR 3-LIKE 1* (*PIL1*), the *CYTOKININ OXIDASES CKX5* and *CKX6*, and *SAUR10*. The architecture phenotype of *ful* mutants, which exhibit more vertical branch growth, can be explained by the de-repression of *SAUR10*, which is specifically expressed at the abaxial side of the branch. *SAUR10* is repressed by FUL in the stem but can be highly induced by a combination of auxin and brassinosteroids and is upregulated by simulated shade. Both the activity of FUL and the light conditions influence the specific expression of *SAUR10* in branches and thereby affects the Arabidopsis branch angle phenotype. *SAUR10* is thus responsive to both developmental and environmental cues and integrates both in the growth response.

## Materials and methods

### Plant materials and growth conditions

Most plants used in this study were in the Col-0 background, including the overexpression and reporter lines and the *ful-7* (SALK_033647) mutant. For the ChIP analysis, FUL-GFP lines were used from a mixed Ler (*ful-1*)/Col-0 background (Urbanus *et al.*, 2009). The JIC SM T-DNA insertion line SM_3_1724 was received from NASC (Tissier *et al.*, 1999) and the FLAG T-DNA line FLAG_590D09 (Samson *et al.*, 2002) was received from the IJPB in Versailles. Plants were grown on rockwool blocks watered with HYPONeX^®^ solution (1.5 g/l), in a long-day climate chamber (16 h/8 h) at 22^o^C. The climate chamber was equipped with LED lights, resulting in the following control conditions: 87.6 μmol m^−2^ s^−1^ photosynthetically active radiation (PAR); R:FR ratio=30.1. Reduced R:FR conditions were achieved by supplemental far red (730 nm) irradiation, resulting in a PAR of 83,5 μmol m^−2^ s^−1^, and a R:FR ratio of 1.15.

### ChIP-Seq analysis

For ChIP-Seq analysis, pistils/siliques in stages 12–16 were harvested from gFUL-GFP lines (Urbanus *et al.*, 2009). The ChIP-Seq and subsequent data analysis were performed according to Kaufmann *et al.* (2010*a*). Input samples were used as controls. The data analyses were largely performed as described by van Mourik *et al.* (2015). Sequences from each ChIP library were mapped to the unmasked *Arabidopsis thaliana* genome (TAIR9) using SOAPv2 (Li and Durbin, 2009). A maximum of two mismatches and no gaps were allowed. Only uniquely mapped reads were retained. Sequence reads mapping to the plastid and mitochondrial genomes were eliminated. The R package CSAR was used for peak calling (Muiño *et al.*, 2011). The ChIP-seq data have been deposited at the NCBI Gene Expression Omnibus (GEO) under accession number GSE79554.

### Electrophoretic mobility shift assays (EMSAs)

*SAUR10* and *SAUR16* coding sequences were amplified from wild-type Col-0 cDNA and cloned into pSPUTK (see Supplementary Table S2 at *JXB* online for all primer sequences). The pSPUTK promoter allowed *in vitro* protein synthesis using the TnT® SP6 High-Yield Wheat Germ Protein Expression System (Promega) according to manufacturers instructions. For *SAUR10*, the probe fragment consisted of a region of 100 bp with the canonical CArG box in the centre. For *SAUR16*, the fragment consisted of a region of 128 bp below the peak summit (see Supplementary Fig. S2 and Table S2 for primer sequences). Promoter fragments were amplified from genomic DNA; the complete FUL coding sequence was amplified from cDNA. The mutated *SAUR10* fragment was generated by overlapping PCR using primers that replaced the canonical CCAAATATGG CArG-box with CCAACGATGG. EMSAs were performed essentially as described by Smaczniak *et al.* (2012) with minor modifications. Oligonucleotides were fluorescently labelled using DY-682. Labelling was performed by PCR using vector-specific DY-682-labelled primers followed by agarose gel extraction. Gel shifts were visualized using a LiCor Odyssey imaging system at 700 nm.

### Generation of transgenic lines

The Gateway technology (Invitrogen) was used for generation of the overexpression and reporter constructs, using the entry vector pDONR221 and the destination vectors pK2GW7 (*35S:SAUR10* overexpression) and pBGWFS7 (*pSAUR10:GUS* and *pFUL:GUS*) (Karimi *et al.*, 2002). See Supplementary Table S2 for all primers. The *FUL-VP16* line was generated by cloning a genomic fragment of the FUL locus, including 3.9 kB upstream region, which was fused in frame with the coding sequence of the strong activation domain of VP16 and followed by the CaMV 35S terminator, into the pBIN19 vector. Constructs were checked by sequencing, transformed into Agrobacterium strains LBA4404 or EHA105 and transformed to Col-0 plants using floral dip (Clough and Bent, 1998).

### Expression analysis

RNA was extracted using the InviTrap^®^ Spin Plant RNA Mini kit (Stratec Molecular) or with a CTAB/LiCl protocol. The RNA concentrations were adjusted to 200 ng/µl and a DNase treatment was performed using Ambion Turbo DNase (AM1907). For qRT-PCR analysis, the RNA was reverse transcribed using the iScript cDNA synthesis kit (BioRad) and the qRT-PCR reaction was performed with iQ SybrGreen supermix from BioRad. The quantitative RT-PCR analyses were performed on the BioRad iCycler. The *UBC21* and/or *TIP41* genes were used as reference genes (Czechowski *et al.*, 2005).

### Hormone treatments and shade experiments

For the hormone treatments, 10 day-old seedlings were removed from plates, containing 2.2 g/l Murashige and Skoog medium (MS), 10% sucrose and 0.8% agar, and incubated in liquid 2.2 g/l MS medium with or without hormones on the shaker for 4 h. The seedlings were floating with their roots submerged in the medium and their leaves contacting the liquid medium. The following hormone concentrations were used: 5 µM IAA (according to Bargmann *et al.*, 2013), 5 µM brassinolide, 5 µM IAA + 5 µM brassinolide. After incubation, seedlings were frozen in liquid nitrogen and stored at −80°C prior to RNA isolation. To investigate the response to simulated shade, seedlings were grown on plates, containing 2.2 g/l MS, 10% sucrose and 0.9% agar, under control conditions for 12 d and then transferred to reduced R:FR for 4 h (see above) or kept for another 4 h under control conditions. To determine the branching phenotypes under simulated shade conditions, plants were placed under reduced R:FR conditions upon bolting and grown for another 2–3 weeks.

### Histochemical analyses

GUS activity was analyzed by staining various tissues overnight at 37°C in staining solution (10 mm EDTA, 0.2% Triton X-100, 1 mm Fe^2+^CN, 1 mm Fe^3+^CN and 1 mg mL^−1^ 5-bromo-4-chloro-3-indolyl-β-glucuronic acid in 50 mm phosphate buffer, at pH 7.2). DR5:GUS tissue was stained for 2 d, refreshing the staining buffer after day one. The tissue was destained for several days in 70% EtOH, and mounted in 30% glycerol for microscopical analysis or photographed while being submerged in 70% EtOH. For DIC microscopy of epidermal cells from the stem, the two components of President coltene^(R)^ dental paste (REF4667), catalyst and base, were mixed together on a glass slide. Before this mixture became solid, stem segments were softly pressed into the paste on the slide. After solidification, the stem segments were removed leaving a print of the outer cell layer. This print was subsequently covered with transparent nail polish, which was removed after hardening. The nail polish layer that contained the cell shapes was then mounted in 100% glycerol and observed under the microscope using DIC optics.

## Results

### FUL binds many genes involved in hormone pathways, including SAUR10 and SAUR16

The MADS domain transcription factor FUL is well known for its role in pistil and silique patterning (Gu *et al.*, 1998; Ferrándiz *et al.*, 2000*b*) and is expressed in a broad range of tissues (Supplementary Fig. S1). To identify direct targets of FUL, we initially focused on pistil and silique tissues, hereafter called silique, and performed a ChIP-Seq experiment with siliques from floral stages 12–16 (Smyth *et al.*, 1990). This resulted in a list of 616 significantly enriched binding sites with a false discovery rate<0.01 (Supplementary Table S1), corresponding to 939 putative target genes with a peak within 3 kb upstream of the ATG and 1 kb downstream of the stop codon. This list showed a 65% overlap with loci identified for the putative FUL interaction partner SEP3 (De Folter *et al.*, 2005; Kaufmann *et al.*, 2009). The FUL ChIP list contains *SHATTERPROOF2* (*SHP2*), a previously identified target of FUL in the silique (Ferrándiz *et al.*, 2000*b*), but no enrichment was detected for *INDEHISCENT* (*IND*) another well described target of FUL (Liljegren *et al.*, 2004), suggesting that the regulation of *IND* by FUL may be indirect.

**Fig. 1.**
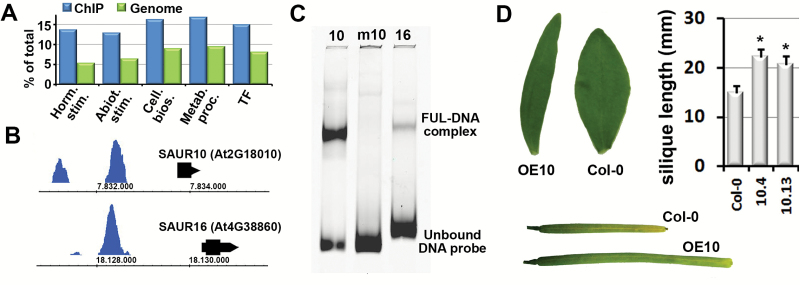
FUL binds many genes involved in hormone pathways, including *SAUR10* and *SAUR16.* (A) Graph showing gene ontology categories in which the FUL targets are significantly over-represented. The y-axis indicates the percentage of genes belonging to this ontology category. A generic GO term finder was used for the analysis (http://go.princeton.edu/cgi-bin/GOTermFinder). The bars represent from left to right: i) response to hormones; ii) response to abiotic stimulus; iii) regulation of cellular biosynthetic process; iv) regulation of primary metabolic process; and v) regulation of transcription, DNA-templated. (B) FUL binding peaks in the upstream regions of *SAUR10* (top) and *SAUR16* (bottom). (C) Binding of the FUL homodimer to different DNA probes in an EMSA assay. Lane 1, *SAUR10* promoter fragment; Lane 2, *SAUR10* promoter fragment with mutated CArG box; Lane 3, *SAUR16* promoter fragment. (D) The overexpression phenotypes of the *35S:SAUR10* lines include longer siliques (picture stage 17B siliques) and differently shaped cauline leaves. See Supplementary Fig. S4 for additional phenotypes. Significant differences from the wild-type (Student’s *t*-test, *P*<0.05) are indicated with an asterisk.

To reveal the processes in which FUL predominantly acts, a gene ontology enrichment analysis was performed (Fig. 1A). Interestingly, the gene category with the highest enrichment in the FUL target set was ‘response to hormones’, directly followed by ‘response to abiotic processes’, indicating that FUL may play an important role in the crosstalk between developmentally and environmentally regulated processes. A closer inspection of the target list revealed in particular many auxin response genes, in addition to gibberellic acid (GA), cytokinin, ABA and ethylene pathway genes (Supplementary Table S1). Two highly enriched, closely related *Small Auxin Upregulated RNA (SAUR*) genes, *SAUR10* and *SAUR16*, were identified, which had FUL binding sites in their promoters, approximately 1380 bp and 1970 bp upstream of the start codon, respectively (Fig. 1B). At the FUL binding positions, the promoter of *SAUR10* contains a canonical CArG box, reported to be commonly bound by MADS domain proteins (Kaufmann *et al.*, 2009), while no clear CArG box was identified in the *SAUR16* promoter (Supplementary Fig. S2). A second, smaller peak was identified in the *SAUR10* promoter about 1200 bp upstream of the large peak, suggesting that *SAUR10* could be regulated by a tetrameric FUL-containing complex that binds to two CArG boxes, as has been shown for MADS complexes involved in floral organ formation (Smaczniak *et al.*, 2012; Theissen and Saedler, 2001). *SAUR10* and *SAUR16* belong to a clade of eight highly homologous *SAUR* genes, comprising *SAUR8*, *SAUR9*, *SAUR10*, *SAUR12*, *SAUR16*, *SAUR50*, *SAUR51*, and *SAUR54* (Kodaira *et al.*, 2011). The genes in this clade have not been functionally characterized yet, but both SAUR9 and SAUR50 have been reported to strongly inhibit PP2C-D activity (Spartz *et al.*, 2014; Atamian *et al.*, 2016), suggesting that proteins of the SAUR10 clade inhibit PP2C-Ds to induce cell expansion via modification of H^+^-ATPases, as has been reported for SAUR19 (Spartz *et al.*, 2014).

To confirm that FUL is able to bind to the upstream regions of *SAUR10* and *SAUR16*, we performed EMSA using the sequence below the peaks as probes (Fig. 1C, see Supplementary Table S2 for the probe sequences). A shift was clearly detected for the *SAUR10* fragment, confirming that FUL can physically bind to this fragment. However, only a faint band indicating a shift was observed for the *SAUR16* fragment, suggesting that FUL is not able to efficiently bind this fragment as a homodimer, in line with the lack of a canonical CArG box in this fragment. Possibly, FUL needs to interact with other transcription factors to strongly bind the *SAUR16* upstream region. To investigate whether the CArG box in the *SAUR10* fragment is essential for FUL binding, we also generated a probe in which the CArG box was disturbed by the mutation of AT to CG in the mid region of the motif (*mSAUR10*). We did not observe a shift for this fragment (Fig. 1C), confirming the importance of the CArG box for the binding of FUL. As FUL is able to bind strongly to sequences in the *SAUR10* promoter *in vivo* and *in vitro*, we further focused on *SAUR10* to unravel its function as direct target of FUL.

### *SAUR10* induces growth

To get an indication of the function of *SAUR10*, we generated an overexpression construct under control of the 35S promoter (*35S:SAUR10*) and transformed it to Col-0 Arabidopsis. The transgenic lines showed pleiotropic growth-related phenotypes, comprising of longer organs and tissues, such as sepals, filaments, etiolated hypocotyls, cauline leaves, pistils and siliques, and a wavy stem (Fig. 1D and Supplementary Fig. S3). These data indicate that *SAUR10* can promote cell elongation similar to other *SAURs* (Chae *et al.*, 2012; Spartz *et al.*, 2012; Stamm and Kumar, 2013; Ren and Gray, 2015). As reported previously for *SAUR36* (Hou *et al.*, 2013), the leaves of the overexpression lines also senesced earlier than the wild-type leaves (Supplementary Fig. S3). We also tested different T-DNA insertion lines for *SAUR10*. However, the insertion in FLAG_590D09 could not be confirmed, while the insertion in SM_3_1724 was found to be located at the 3’ end of the coding sequence and didn’t affect the expression of *SAUR10*. The phenotypes of these lines were similar to the wild-type.

### FUL is a pleiotropic regulator of plant growth and architecture

**Fig. 2.**
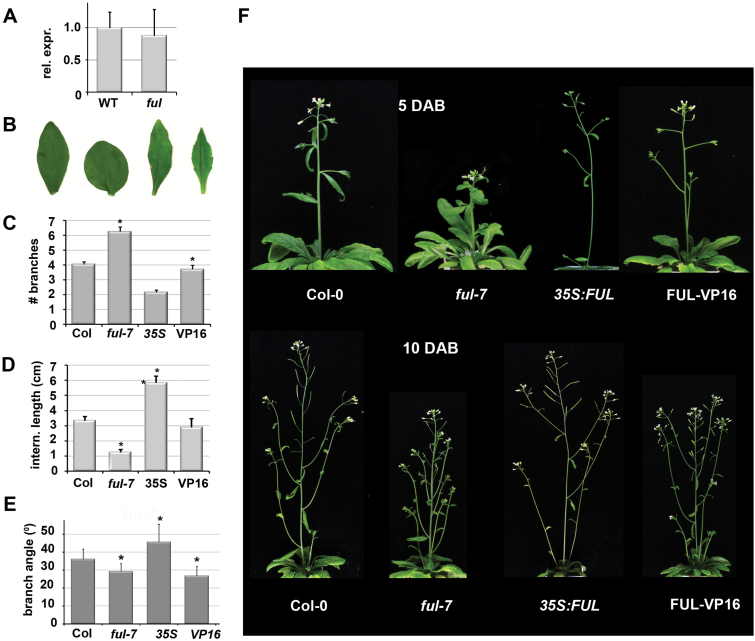
FUL is a pleiotropic regulator of plant growth and architecture. (A) Expression of *SAUR10* in wild-type and *ful-7* siliques from stages 12–16. (B) Cauline leaf phenotypes of Col-0, *ful-7*, *35S:FUL*, and FUL-VP16 leaves (from left to right). (C) The number of branches formed along the main inflorescence. (D) Average internode length between the branches in plants at 10 DAB. (E) Average branch angle of all side branches along the primary stem of plants around 12 DAB. (F) Architecture phenotypes of Col-0, *ful-7*, *35S:FUL*, and FUL-VP16 plants at 5 DAB (upper panel) and 10 DAB (lower panel). In (A), the error bars represent the SE based on two biological replicas. In (C–E), the error bars represent the SE based on at least 20 measurements. Significant differences from the control (Student’s *t*-test, *P*<0.05) are indicated with an asterisk.

To investigate the role of FUL in the regulation of *SAUR10* in the silique, we performed a quantitative RT-PCR experiment (qPCR) to compare the expression of *SAUR10* in wild-type and *ful-7* (SALK_033647) mutant siliques from flower stages 11–14 (Smyth *et al.*, 1990). However, we didn’t find an expression difference, suggesting that *SAUR10* is not regulated by FUL in the silique (Fig. 2A).

We therefore hypothesized that there could be other tissues in which FUL regulates *SAUR10*, possibly explaining some of the growth-related phenotypes observed in *ful* mutants (Fig. 2B–F). In addition to the well described phenotypes in the silique and inflorescence meristem (Gu *et al.*, 1998; Ferrándiz *et al.*, 2000*a*), *ful* mutants also exhibit an altered cauline leaf shape (Fig. 2B, Gu *et al.*, 1998). We also noticed distinct differences in stem development and architecture, including enhanced branching, decreased inflorescence branch angles, and shorter stem and internode lengths in *ful-7* mutants (Fig. 2C–F and Supplementary Fig. S4). Shortly after bolting, wild-type stems elongate considerably, reaching a length on average of 12.8 cm (+/-5.4 cm) at 5 d after bolting (DAB), while *ful-7* stems hardly elongated at that stage and are only on average 5.3 cm (+/- 2.8 cm, *P*<0.001) long. The difference in stem length is still visible at 10 DAB but disappears when the plants grow older (Fig. 2F and Supplementary Fig. S4). *35S:FUL* overexpression lines exhibit a phenotype opposite to *ful-7*, with enhanced stem elongation and increased internode size in combination with reduced branch numbers and increased branch angles (Fig. 2B–F).

We compared the phenotypes of *ful-7* mutants and wild-type plants to a *pFUL:FUL-VP16* line (FUL-VP16). This line consists of a translational fusion of FUL with the strong transcriptional activation domain of the herpes virus protein VP16, driven by the *FUL* promoter. Genes that in wild-type plants are repressed by *FUL* are expected to become activated in this line. Phenotypes of *ful* mutants that are caused by target gene de-repression should thus be similar in the FUL-VP16 line, albeit probably to a lesser extent because FUL-VP16 has been generated in the Col-0 background and still contains an endogenous FUL copy that can repress the targets. The FUL-VP16 plants showed aberrant phenotypes that were probably a mix of enhanced target gene activation, for those targets that in wild-type tissues are activated by FUL, and activation of targets that are normally repressed by FUL. For example, FUL-VP16 siliques exhibited ‘shoulders’ and a short style similar to *35S:FUL* siliques, while their overall phenotype more closely resembled that of *ful-7* siliques (Supplementary Fig. S5A). To verify that FUL-VP16 is not co-suppressing endogenous *FUL*, we tested *FUL* transcript abundance in stem and branches and found a 1.5–2-fold higher expression of *FUL* (Supplementary Fig. S5B), indicative of the presence of an additional *FUL* copy (FUL-VP16) and not of co-suppression. Several FUL-VP16 phenotypes, including stem and cauline leaf growth, were more similar to *35S:FUL* than to *ful-7*, suggesting that these traits are largely regulated by activation of target genes in the wild-type (Fig. 2B, F).

### FUL represses SAUR10 in the stem

**Fig. 3.**
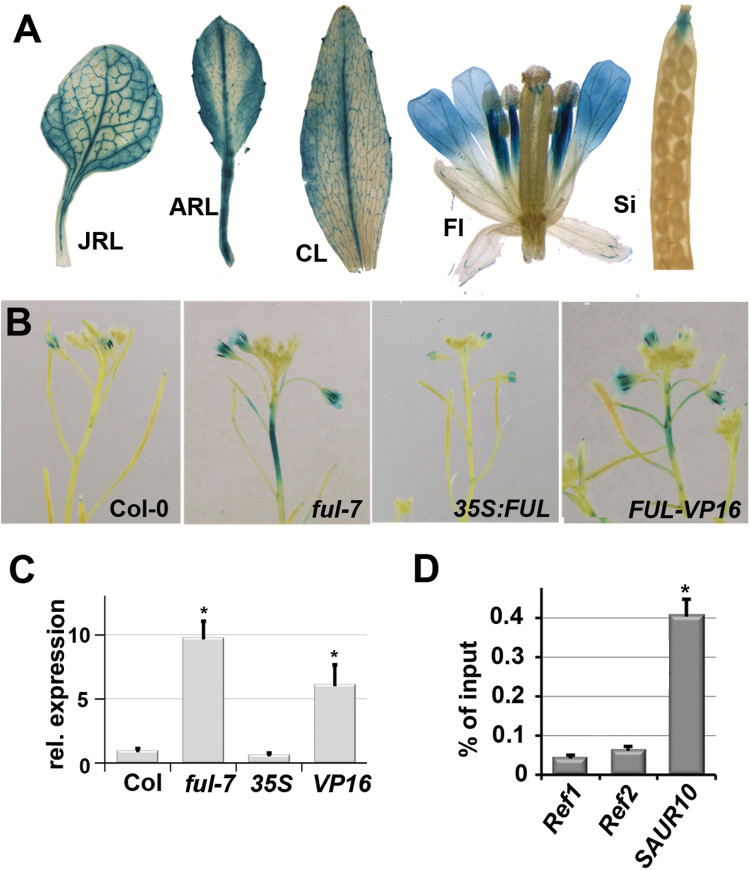
*SAUR10* is regulated by FUL in stems. (A) GUS staining pattern in different organs and tissues of a *pSAUR10:GUS* line. JRL, juvenile rosette leaf; ARL, adult rosette leaf; CL, fully expanded cauline leaf; Fl, flower stage 13; Si, silique stage 17. (B) GUS staining pattern of *pSAUR10:GUS* inflorescence stems from different FUL backgrounds. (C) Relative expression of *SAUR10* in the upper stem segment of Col-0, *ful-7*, *35S:FUL*, and FUL-VP16 plants 8–10 DAB. (D) Graph from a ChIP-qPCR experiment showing the enrichment of the *SAUR10* fragment relative to two reference sequences in a ChIP sample from stem tissue. The enrichment of the fragments was calculated as a percentage of the input sample. Error bars represent the SE of three biological replicas in the case of expression analyses, and two replicas for the ChIP-PCR. Significant differences from the control (Student’s *t*-test, *P*<0.05) are indicated with an asterisk.

To investigate whether FUL can regulate *SAUR10* in tissues other than the silique, we generated reporter lines for *SAUR10* (*pSAUR10:GUS*) and crossed these into the *ful-7*, *35S:FUL* and *FUL-VP16* backgrounds. The *GUS* expression patterns of the *pSAUR10:GUS* lines was rather specific, with staining predominantly in the veins and petioles of rosette leaves and cauline leaves. In the context of the flower, expression appeared only in stage 12 flowers in the vasculature of the style, and in stage 13 flowers in the apical parts of the stamen filaments and petals. Apart from the expression in the style, no expression was observed in the pistil or silique (Fig. 3A).

*SAUR10* expression in *ful-7* and FUL-VP16 stems was clearly different from expression in wild-type plants. In wild-type inflorescences, *pSAUR10:GUS* expression was not, or only very weakly, observed in the stem. However, in both *ful-7* and FUL-VP16 inflorescences, GUS expression was present in the upper stem region, indicating that *SAUR10* is de-repressed in *ful-7* and activated by FUL-VP16 in inflorescence stems (Fig. 3B). To investigate whether *SAUR10* transcript levels were in line with these results, RNA was extracted from a 1 cm stem segment directly below the inflorescence meristem, and qPCR was performed. *SAUR10* expression was found to be approximately 10-fold higher in *ful-7* and 6-fold higher in FUL-VP16, while the levels in *35S:FUL* were not significantly decreased, probably because *SAUR10* expression is already very low in wild-type stems (Fig. 3C). These data are consistent with direct repression of *SAUR10* by FUL, and are in line with our observation that *pFUL:GUS* is active in the inflorescence stem (Supplementary Fig. S1).

Since our ChIP-Seq experiment was performed with silique tissue, we were not certain if FUL could directly bind to *SAUR10* in the stem. To test this, we performed ChIP-qPCR experiments using stem tissue of primary and secondary inflorescences. We found a distinct enrichment for *SAUR10* in both replicates (Fig. 3D), showing that FUL can directly bind to the *SAUR10* locus in stems as well. In conclusion, we show here that FUL can directly repress *SAUR10* expression in the stem.

### Auxin and BR induce SAUR10 expression synergistically

**Fig. 4.**
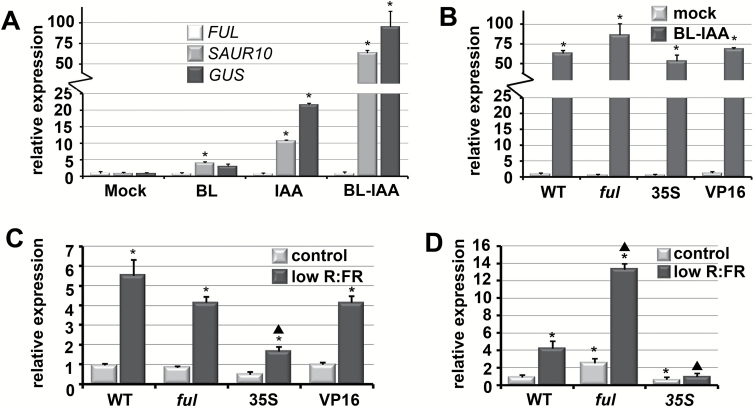
*SAUR10* is induced by a combination of BRs and auxin, and by reduced R:FR ratios, which can be repressed by FUL in the stem. (A) Expression of *SAUR10*, *GUS* (in *pSAUR10:GUS* lines) and *FUL* in 10 day-old seedlings after a 4 h treatment with auxin (IAA), BRs (BL) or a combination of both (BL-IAA). (B) Expression of *SAUR10* in 10 day-old Col-0, *ful-7*, *35S:FUL*, and FUL-VP16 seedlings after a 4 h treatment with a combination of auxin and BRs (BL-IAA). (C) Expression of *SAUR10* in Col-0, *ful-7*, *35S:FUL*, and FUL-VP16 12 day-old seedlings grown for 4 h under reduced R:FR light conditions or under control conditions. (D) Expression of *SAUR10* in Col-0, *ful-7*, *35S:FUL*, and FUL-VP16 stems 7 DAB, grown for 4 h under reduced R:FR light conditions or under control conditions. The error bars represent the SE of three biological replicas. In (A) and (B), significant differences from the mock control (Student’s *t*-test, *P*<0.05) are indicated with an asterisk. In (C) and (D), significant differences from the wild-type control situation (Student’s *t*-test, *P*<0.05) are indicated with an asterisk, while significant differences from the wild-type low R:FR situation are indicated with a triangle.

Experiments with auxin have identified *SAUR10* as one of the Arabidopsis *SAUR* genes clearly upregulated in response to auxin treatment in seedlings (Goda *et al.*, 2004; Paponov *et al.*, 2008; Chapman *et al.*, 2012; Bargmann *et al.*, 2013). *SAUR10* has also been reported to be responsive to brassinosteroids (BRs) (Yu *et al.*, 2011). To investigate the interaction between FUL-controlled repression and hormone-induced upregulation, we treated wild-type, *ful-7*, *35S:FUL*, and FUL-VP16 seedlings with auxin, namely 5 µM indole-3-acetic acid (IAA), and/or BRs, namely 5 µM brassinolide (BL), for 4 h. Wild-type seedlings treated with BL showed a 4-fold induction of *SAUR10* compared with mock-treated seedlings, while the seedlings treated with auxin showed a 10-fold increase, confirming the previously published hormone responses (Fig. 4A). Interestingly, a combination of IAA and BL resulted in a synergistic effect on the induction of transcription and led to an impressive 65-fold higher expression of *SAUR10* in seedlings. In all treatments, the expression of *FUL* did not change, while the *GUS* transcript levels in treated *pSAUR10:GUS* plants showed a response similar to *SAUR10*, indicating that hormone induction is regulated by the promoter rather than through post-transcriptional mechanisms. To test whether FUL could influence the hormone-induced increase in *SAUR10* expression, we performed the IAA-BL treatments in *ful-7*, *35S:FUL*, and FUL-VP16 seedlings. This revealed no significant differences (Fig. 4B), suggesting that FUL does not influence the hormonal upregulation of *SAUR10* in seedlings.

### The response of SAUR10 to shade is influenced by FUL

*SAUR10* can be highly induced by a combination of auxin and BRs, two hormones that have together been associated with shade responses (Pierik *et al.*, 2009; Keuskamp *et al.*, 2011). When exposed to shade, plants sense a decreased R:FR ratio, as well as depletion of blue light, and respond by phenotypic changes such as stem, internode and petiole elongation, and hyponastic leaf movement, together referred to as the shade-avoidance syndrome (SAS). To investigate whether *SAUR10* is responsive to simulated shade, we transferred 12 day-old seedlings to low R:FR light conditions and compared the expression of the genes with seedlings from the control condition. *SAUR10* showed a marked increase of expression after 4 h of low R:FR, indicating that it can indeed positively respond to shade. This response was not different in *ful* or FUL-VP16 seedlings, but was significantly reduced in *35S:FUL* seedlings, suggesting that the ectopic overexpression of FUL represses the shade-induced upregulation of *SAUR10* (Fig. 4C).

To investigate whether the effect of FUL was more distinct in the tissue where it actually represses *SAUR10*, we transferred wild-type, *ful*, and *35S:FUL* plants to low R:FR conditions and harvested stem segments after 4 h. The expression in wild-type stems was upregulated four times compared with control stems, while the upregulation in *ful* mutant stems increased to 14 times (Fig. 4D). The upregulation in *ful* stems is higher than can be explained by an additive effect of de-repression and shade-induced upregulation, suggesting that loss-of-FUL allows a greater response to the light conditions. No significant upregulation compared with control conditions could be detected at all in *35S:FUL* stems (Fig. 4D), pointing to a much stronger effect of FUL repression in the regulation of *SAUR10* expression in the stem than in seedlings. In conclusion, these experiments show that *SAUR10* is a distinct responder to both hormone and light stimuli, and that the light response can be attenuated by FUL in the stem.

### De-repressed SAUR10 expression correlates with longer cells in the stem

**Fig. 5.**
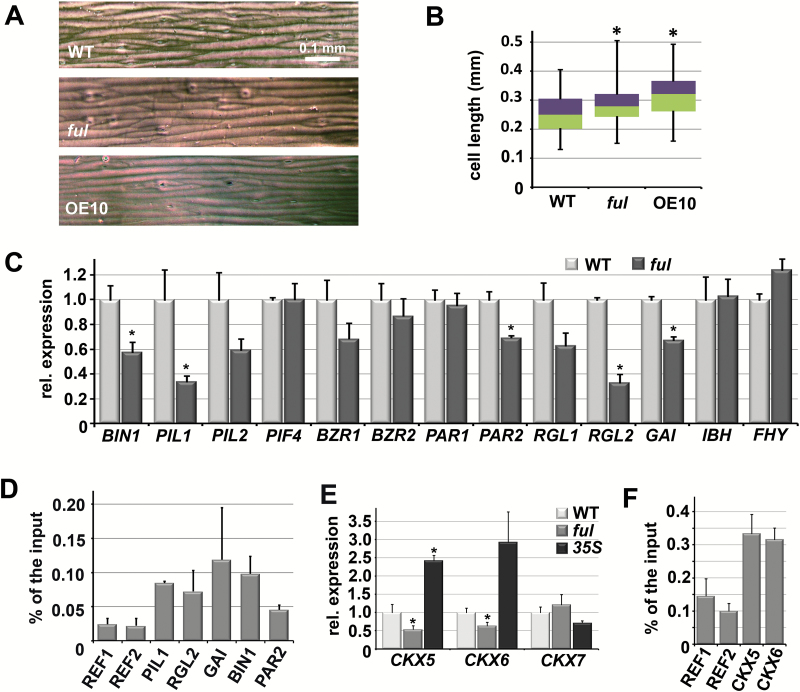
The *ful* mutant stem phenotype is due to a combination of de-regulated genes. (A) Prints of stem segments between internode 1 and 2 from wild-type, *ful-7*, and *35S:SAUR10* stems. The scale bar represents 0.1 mm. (B) Boxplot showing the cell lengths of the stem segments such as in panel (A). The distribution of the lengths is depicted as follows: purple, upper quartile; green, lower quartile; upper error bar, maximum; lower error bar, minimum. Cell lengths were measured with ImageJ and based on at least 40 cells from three different stem segments. (C) Relative expression of a number of genes from the ChIP-seq target list in wild-type and *ful-7* stems. (D) Graph from a ChIP-qPCR experiment showing the enrichment of the *PIL1, RGL2, RGA2*, and *BIN1* fragments relative to two reference sequences in a ChIP sample from stem tissue. The enrichment of the fragments was calculated as a percentage from the input sample. (E) Relative expression of *CKX5, CKX6* and *CKX7* in the upper stem segment of wild-type, *ful-7*, and *35S:FUL* inflorescences. The significance of the differential expression in the *ful-7* samples compared to the wild-type was calculated based on five biological replicates. (F) Graph from a ChIP-qPCR experiment showing the enrichment of the *CKX5* and *CKX6* fragments relative to two reference sequences in a ChIP sample from stem tissue. The enrichment of the fragments was calculated as a percentage of the input sample. Error bars represent the SE of three biological replicas in the case of expression analyses. Significant differences from the control (Student’s *t*-test, *P*<0.05) are indicated with an asterisk. Two replicas were performed for the ChIP-PCR. (This figure is available in colour at *JXB* online)

We inspected the stem and architecture phenotype of the *ful* mutants further to identify phenotypic features that could be attributed to *SAUR10* de-repression. Given the longer organ phenotype of the *SAUR10* overexpression lines, we expected the inflorescence stem of *ful-7* plants, in which *SAUR10* is more highly expressed, to be longer than the wild-type, and that of *35S:FUL* to be shorter. However, we found the opposite effect in young inflorescences, which were shorter in *ful-7* plants, with a significantly smaller distance between side branches (Fig. 2D) and silique internodes (Supplementary Fig. S4). To determine whether the cell sizes in the stem were in line with the internode sizes, we measured cell length in wild-type, *ful-7*, and *35S:SAUR10* stems between internodes one and two. Interestingly, this revealed longer cells in the *ful-7* and *35S:SAUR10* stems compared with wild-type (Fig. 5A, B), showing that *ful-7* stems have longer cells despite having shorter internodes. Thus, the upregulation of *SAUR10* in the *ful-7* stem probably does result in longer cells, but the shorter stem phenotype is caused by reduced cell division as a result of de-regulation of other target genes.

### The ful mutant stem phenotype is caused by a combination of de-regulated genes

FUL represses *SAUR10* in the stem, but the stem phenotype of the *ful-7* line, which shows retarded cell division, indicates that FUL additionally activates other targets to regulate stem growth and architecture. We therefore examined the FUL ChIP target list in more detail for genes that have been associated with growth responses. In addition to genes involved in cytokinin and auxin signalling, such as the cytokinin degradases *CKX5*, *CKX6*, and *CKX7*, and genes encoding the AUX/IAA proteins IAA8 and IAA16, we also found a remarkable number of genes that are implicated in the light-sensitive growth of hypocotyls, encoding transcription factors involved in the BZR-PIF-ARF-DELLA pathway (Bai *et al.*, 2012; Oh *et al.*, 2014). These include the PIF genes *PIL1*, *PIL2*, and *PIL4*, the DELLA genes *RGL1*, *RGL2*, and *GAI*, the BR pathway genes *BZR1* (only one replica), *BZR2*, and *BRASSINOSTEROID INSENSITIVE 1* (*BIN1*), and also the photoreceptor phytochrome A (FHY) and its targets *PHYTOCHROME RAPIDLY REGULATED 1* (*PAR1*) and *PAR2*, which are negative regulators of the shade response and reduce the expression of several *SAURs* (Roig-Villanova *et al.*, 2007).

**Fig. 6.**
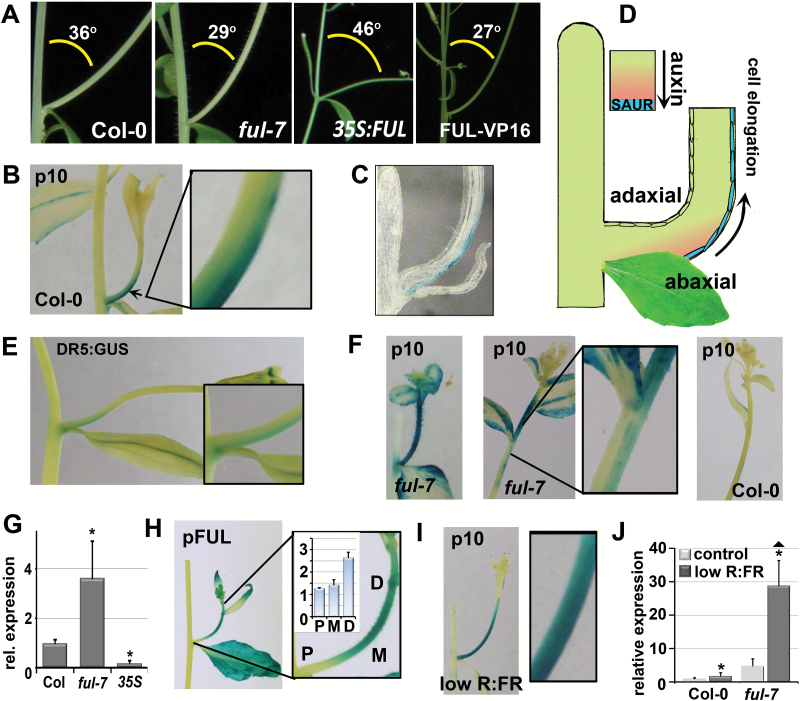
The branch angle phenotype of the *ful* mutant correlates with specific abaxial expression of *SAUR10* in branches. (A) Pictures showing the relation between branch angle and plant architecture in the different FUL backgrounds. The smaller the angle, the more vertical the branches grow. The average branch angle in the different backgrounds is indicated. (B) Localization of *pSAUR10:GUS* at the abaxial side of a young branch. The arrow points to the region that was enlarged. (C) Cross-section of a *pSAUR10:GUS* branch. (D) Model showing the correlation between auxin-induced accumulation of growth factors, SAURs, at the abaxial side and branch bending. (E) Localization of DR5:GUS at the abaxial side of the branch. (F) Expression and localization of *pSAUR10:GUS* in the branches of Col-0 and *ful-7*. Left panel: *pSAUR10:GUS* can be observed on both sides of emerging *ful-7* branches. The middle and right panels show older, elongating branches, where no signal is visible any more at the proximal part near the primary stem, but a difference in the distal part can be observed. *pSAUR10:GUS* is clearly visible in the distal region of elongating *ful-7* branches, and is specifically abaxially expressed directly below that region (middle panel), whereas *pSAUR10:GUS* is not visible in the distal region of elongating older Col-0 branches (right panel). (G) Relative transcript levels of *SAUR10* in young branches from different FUL backgrounds. (H) *FUL* is highly expressed throughout branches, with the highest expression in the distal region. P, proximal region; M, middle region; D, distal region. (I) Localization of *pSAUR10:GUS* on both sides of branches grown under reduced R:FR light conditions. (J) Relative transcript levels of *SAUR10* in Col-0 and *ful-7* branches under control conditions and reduced R:FR light conditions. p10, pictures from *pSAUR10:GUS* stained tissue; pFUL, pictures from pFUL:GUS stained tissue. The error bars depict the SE based on three biological replicas. Significant differences (Student’s *t*-test, *P*<0.05) are indicated with an asterisk.

To determine if any of these genes were regulated by FUL in the stem, we performed qPCR analysis to compare the transcription levels of *ful-7* mutant stems with wild-type. We detected significantly lower transcript levels for *BIN1*, *PIL1*, *RGL2*, *GAI*, and *PAR2*, indicating that FUL activates these genes in wild-type stems (Fig. 5C). We selected these targets to test whether they were also bound by FUL in the stem, and found enrichment for all five tested genes in two independent ChIP experiments (Fig. 5D). However, the decreased expression of these genes will have an effect on cell elongation rather than on cell division (Nam and Li, 2002; Salter *et al.*, 2003; Roig-Villanova *et al.*, 2007; Li *et al.*, 2012) and can thus not entirely explain the *ful* stem phenotype. *CKX5*, *CKX6*, and *CKX7,* on the other hand, are cytokinin oxidase/dehydrogenase (CKX) genes, which can catalyze the degradation of cytokinins and have been reported to determine the activity of the shoot meristem. We found that the transcript levels of *CKX5* and *CKX6* are significantly decreased in *ful-7* stems, while upregulated in *35S:FUL* stems (Fig. 5E). This would lead to reduced cytokinin breakdown in *ful* mutants and a higher meristem activity (Werner *et al.*, 2003; Bartrina *et al.*, 2011), which could explain the increased branching and shorter internodes as observed. We also tested whether *CKX5* and *CKX6* were bound by FUL in the stem and detected a clear enrichment for the *CKX5* and *CKX6* loci compared with two reference loci (Fig. 6D). FUL thus appears to regulate a complex network of genes that are likely to have opposite functions in stem growth. The outcome of this regulation probably depends on other factors that interfere with this network, such as hormone concentration and light quality.

### De-repressed SAUR10 expression correlates with decreased branch angles

We also noticed that the branch angles in *ful-7* and FUL-VP16 plants are significantly smaller than in the wild-type, while being larger in *35S:FUL* (Figs 2E and 6A). Branch angle is highly dynamic and depends on gravitropic and phototropic signals, both of which depend on auxin gradients (Roychoudhry *et al.*, 2013; Liscum *et al.*, 2014). In addition, BRs have also been found to influence auxin-mediated phototropic responses (Liscum *et al.*, 2014). We therefore investigated whether the altered branch angles in *ful* mutants could be correlated to *SAUR10* expression. *pSAUR10:GUS* activity was specifically observed at the abaxial side of young Col-0 branches and sectioning revealed that expression was located in the abaxial epidermal layer (Fig. 6B, C), pointing to a role for *SAUR10* in branch bending. Since directed auxin-induced hypocotyl/stem elongation occurs in accordance with the SAUR-mediated acid growth theory and growth is predominantly regulated by the epidermis (Fendrych *et al.*, 2016; Procko *et al.*, 2016), we reasoned that *SAUR* expression in the abaxial epidermal cell layer is expected to enhance cell elongation on this side, resulting in more vertical growth of the branch (Fig. 6D). To determine if auxin concentrations are higher at the abaxial side of the branch, we examined the DR5:GUS auxin reporter line (Ulmasov *et al.*, 1997). This revealed a weak GUS signal in DR5:GUS branches, specifically at the abaxial side after prolonged staining (Fig. 6E), indicating that auxin levels are indeed higher at the abaxial side of the branch, probably inducing *SAUR10* expression.

Similar to the de-repression of *SAUR10* in the primary inflorescences of *ful-7* and *FUL-VP16* plants, *SAUR10* was also clearly upregulated in the stem of the branch inflorescences. We examined the *pSAUR10:GUS* pattern in detail in the *ful-7* line and found that in young, emerging branches, the GUS signal was higher than in the wild-type and could also be observed at the adaxial side (Fig. 6F). This upregulated *pSAUR10:GUS* signal remained distally visible when the branch elongated and was accompanied by a region with distinct abaxial GUS expression just below the distal region. In contrast, *pSAUR10:GUS* was rarely observed in wild-type branches at this stage (Fig. 6F). In accordance with FUL repressing *SAUR10* in branches, the *pSAUR10:GUS* signal was mostly absent in *35S:FUL* branches (Supplementary Fig. S6A). These *pSAUR10:GUS* data were confirmed by qPCR analysis of *SAUR10* transcript levels in branches 4–6 DAB, which revealed a 3–4-fold higher expression in *ful-7* and a 5-fold reduction in *35S:FUL* (Fig. 6G). The higher abaxial expression in the region below the *ful-7* (Fig. 6F) and FUL-VP16 (Supplementary Fig. S6A) inflorescences can well explain the more vertical branching in these lines. The expression of *FUL* itself also corresponds with the observed *SAUR10* expression, as *FUL* is highly expressed throughout branches and exhibits the highest levels just below the inflorescences (distal part, Fig. 6H). This is in line with the observation that *SAUR10* de-repression is most prominent in this region.

### Abaxial SAUR10 expression is affected by light conditions

To investigate whether the expression of *pSAUR10:GUS* in branches was sensitive to light conditions, we reduced the R:FR ratio and determined the GUS pattern in the inflorescences after 24 h. We observed a relocation of the GUS signal to both sides of the branch (Fig. 6I), consistent with a positive response of *SAUR10* to the shaded, low R:FR conditions at both the adaxial (Ad) and abaxial (Ab) sides and thus an increase of the Ad/Ab ratio. In line with this result, plants that were exposed for a longer period to reduced R:FR conditions exhibited substantially increased branch angles (Supplementary Fig. S7A, B), suggesting a more homogenous growth factor distribution. If *SAUR10* is indeed regulating branch bending, branch angle should also be disturbed in *35S:SAUR10* plants, where *SAUR10* is ectopically expressed at the adaxial side. The expected increased Ad/Ab ratio would then result in more horizontal branch growth. Indeed, we observed larger branch angles in the *35S:SAUR10* line, although branch growth was highly variable and irregular (Supplementary Fig. S7A, B). This irregular growth was even stronger under reduced R:FR conditions (Supplementary Fig. S7B), suggesting that the *SAUR10* overexpression phenotype is enhanced by the SAS response.

We also tested to what extent FUL could influence the effect of simulated shade on *SAUR10* expression levels and branch angle phenotype. In branches of the *ful* mutant, de-repression of *SAUR10* combined with shade-induced expression resulted in an almost 30-fold upregulation of *SAUR10* in young branches (Fig. 6J). This is again more than can be explained by an additive effect alone, suggesting that the absence of FUL allows an enhanced response to the light conditions, causing an increase in expression on both the abaxial and adaxial sides. In line with this, simulated shade resulted in more horizontal branch growth in all backgrounds (Supplementary Fig. S6B).

In conclusion, we found *SAUR10* to be abaxially expressed in branches, presumably as a result of auxin accumulation at the shaded side of the branch. The de-repression of *SAUR10* in the *ful* mutant results in increased abaxial expression in the distal part of the branch, which can cause the increased bending of the branches observed in *ful* mutants. In wild-type branches, FUL represses *SAUR10*, thereby possibly preventing over-bending of the branch and attenuating responses to light conditions.

## Discussion

We show here that the MADS domain transcription factor FUL is a pleiotropic regulator of plant development, which plays important roles in plant growth and architecture in addition to its well known functions in fruit development and flowering time. In particular the deviating branch angles in the *ful* mutants are very interesting, since little is known about this trait, which is particularly important for crop yield. Loci in other species that could be linked to branch angle have been associated with the auxin pathway, such as the LA1 locus in rice (Li *et al.*, 2007) and the *GRETCHEN HAGEN 3* (*GH3*) gene in *Brassica napus* (Liu *et al.*, 2016). Our analysis also indicates that members of the *SAUR* family can play an important role in branch bending, especially in response to environmental conditions like high plant density. We demonstrate that *SAUR10* is specifically expressed at the abaxial side of the branch, thereby affecting branch angle. Enhanced and prolonged abaxial expression of *SAUR10* in the *ful* mutant can explain its more vertical branching phenotype. The activity of *SAUR10* in stems and branches appears to be regulated by interplay between hormone-induced upregulation and FUL-controlled repression. In addition, our data reveal that FUL directly regulates a number of other genes involved in hormone and light signalling, of which the de-regulation contributes to the *ful* mutant phenotype. To what extent these genes contribute to the *ful* mutant phenotype needs to be further investigated. However, a picture is emerging that FUL can regulate plant growth and architecture in concert with the environment by balancing the expression of hormone and light responsive factors. It will be interesting to study how the expression of FUL changes during plant development, and if for example, older plants are less responsive to environmental signals through increased FUL expression.

### FUL interacts with the IAA/BR pathway to repress SAUR10

Despite the broad expression pattern of FUL, de-repression of *SAUR10* in the *ful* mutant only occurs in a limited number of tissues, indicating that *SAUR10* activation requires additional tissue-specific factors, such as high auxin and BR levels. In addition to repressing *SAUR10* under control conditions, we also found that FUL can buffer the hormone- or shade-induced expression of *SAUR10* in stems and branches, suggesting that FUL can attenuate the activity of the auxin and/or BR response transcription factors. Since *SAUR10* has been identified as a direct target of both ARF6 and BZR1 by ChIP-Seq analyses (Oh *et al.*, 2014), it is possible that FUL can interact with either or both of these factors, thereby repressing transcription. FUL binds to a CArG box in the *SAUR10* promoter, which is located 260 bp upstream of a canonical ARF binding motif and only 100 bp downstream of an AuxRE-related element identified by Walcher and Nemhauser (2012). Binding of FUL to the CArG box may disturb the interaction between ARF6 and BZR1 (previously reported by Oh *et al.*, 2014). Our results indicate that FUL is not required to determine the *SAUR10* expression domain, but rather to fine-tune or buffer the response to hormonal stimuli.

### SAURs integrate environmental, hormonal, and developmental signals in the growth response

Different studies in Arabidopsis, soybean and maize have identified *SAUR* genes as hormone-responsive growth regulators. However, *SAUR*s have also been found as direct targets of several transcription factors functioning in plant development, suggesting that they function downstream of both developmental and hormonal regulators to direct plant growth. We demonstrate here that *SAUR10* is regulated by hormonal stimuli, light signals, as well as by the developmental regulator FUL, and can thereby integrate a plethora of signals in the growth response. Several recent reports have strengthened the idea that *SAUR*s can in general respond to a variety of upstream factors, integrating these in the regulation of cell elongation through interaction with PP2C-Ds (Spartz *et al.*, 2014; Challa *et al.*, 2016; Procko *et al.*, 2016; Sun *et al.*, 2016). This has been most thoroughly investigated in seedlings, where *SAUR* genes have been grouped according to their response to light conditions in hypocotyls and cotyledons (Sun *et al.*, 2016), indicating that the growth response is controlled by a cluster of similarly regulated *SAUR*s, rather than by single genes. In that respect, *SAUR10* may not be the only *SAUR* with differential expression between the abaxial and adaxial side of the Arabidopsis branches. Interestingly, a *SAUR50*-like gene - *SAUR50* belongs to the *SAUR10*-clade - has recently been identified to be responsible for heliotropism in sunflower (Atamian *et al.*, 2016). The *SAUR50*-like gene is expressed more highly on the east side of the stem during the day, enabling the shoot apex to move gradually from east to west along with the sun. In addition, several other *SAUR*s have been reported to be responsive to shade (Roig-Villanova *et al.*, 2007; Spartz *et al.*, 2012; Procko *et al.*, 2016), indicating that the dynamic response to shade may to a large extent be executed by SAUR proteins. Differential expression of *SAUR* genes may thus in general allow directional growth in a variety of species.

## Supplementary data

Supplementary data are available at *JXB* online.

Table S1. Identified loci with significant enrichment in the FUL ChIP-Seq

Table S2. Primer list.

Fig. S1. FUL is widely expressed in Arabidopsis.

Fig. S2. Binding sites of FUL in the *SAUR10* and *SAUR16* upstream regions.

Fig. S3. Phenotypes of the *SAUR10* overexpression lines.

Fig. S4. Distance between the silique internodes.

Fig. S5. Characterization of the FUL-VP16 plants.

Fig. S6. FUL represses *SAUR10* in branches, which can be correlated to branch angle.

Fig. S7. The architecture of Col-0 and 35S:SAUR10 plants changes under reduced R:FR conditions.

## Data deposition

ChIP-Seq data. Gene Expression Omnibus (GEO). Accession number GSE79554.

https://www.ncbi.nlm.nih.gov/geo/query/acc.cgi?acc= GSE79554

## Supplementary Material

Supplementary Figures S1-S7

Supplementary Tables S1-S2

## Abbreviations:

ABA: abscisic acid
BL: brassinolide
BRs: brassinosteroids
CKX: cytokinin oxidase/dehydrogenase
DAB: days after bolting
FUL: FRUITFULL
GA: gibberellic acid
IAA: indole-3-acetic acid
SAUR: *SMALL AUXIN UPREGULATED RNA.*
