# Supplementary material for: FRUITFULL controls SAUR10 expression and regulates Arabidopsis growth and architecture
Source: J Exp Bot. 2017 Jun 6;68(13):3391–403. doi: 10.1093/jxb/erx184 (PMC5853401; doi:10.1093/jxb/erx184)
Supplement: Supplementary Figures S1-S7 [file erx184_suppl_supplementary_figures_s1-s7.pdf]

Supplementary data

Figures

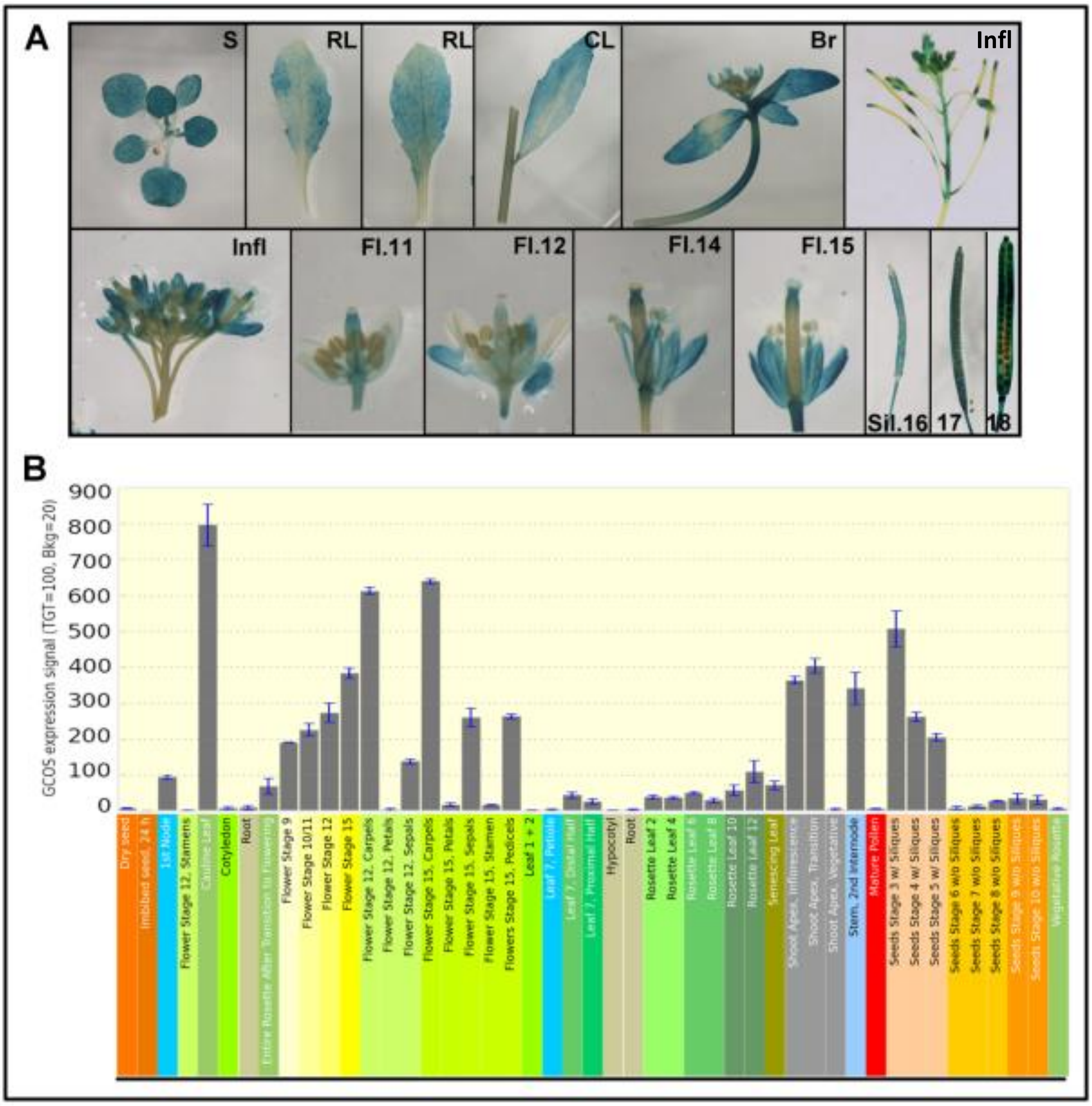

Figure S1. FUL is widely expressed in Arabidopsis. A) GUS staining of tissues from a pFUL:GUS line after overnight staining. S = 14 day old seedling/rosette, RL = rosette leaf, CL = cauline leaf, Br = side branch, Infl = inflorescence, FL.11 = flower stage 11, Fl.12 = flower stage 12, Fl.14 = flower stage 14, Fl.15 = flower stage 15, Sil. 16 = silique stage 16, 17 = silique stage 17, 18 = silique stage 18 (stages according to Smyth et al. (1990)). **B)** Expression profile for FUL based on large-scale transcriptome datasets, visualized with the Efp browser (http://bar.utoronto.ca/efp/cgi-bin/efpWeb.cgi) (Winter et al., 2007. PLoS One 2(8)).

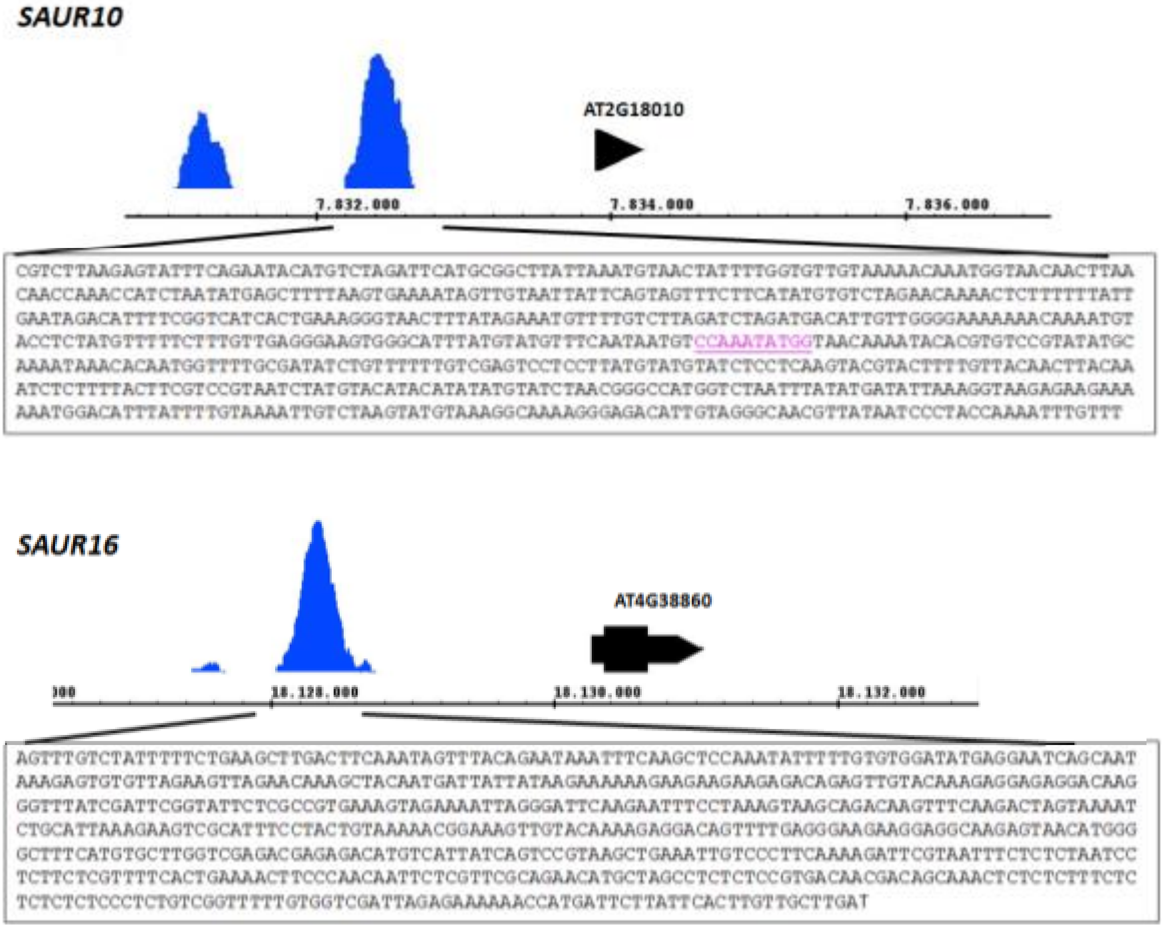

Figure S2. Binding sites of FUL in the *SAUR10* and *SAUR16* upstream regions. The depicted sequences are part of the upstream regions of *SAUR10* (upper panel) and *SAUR16* (lower panel) , spanning the peak region. The canonical CArG-boxes in the SAUR10 promoter is shown in purple and underlined.

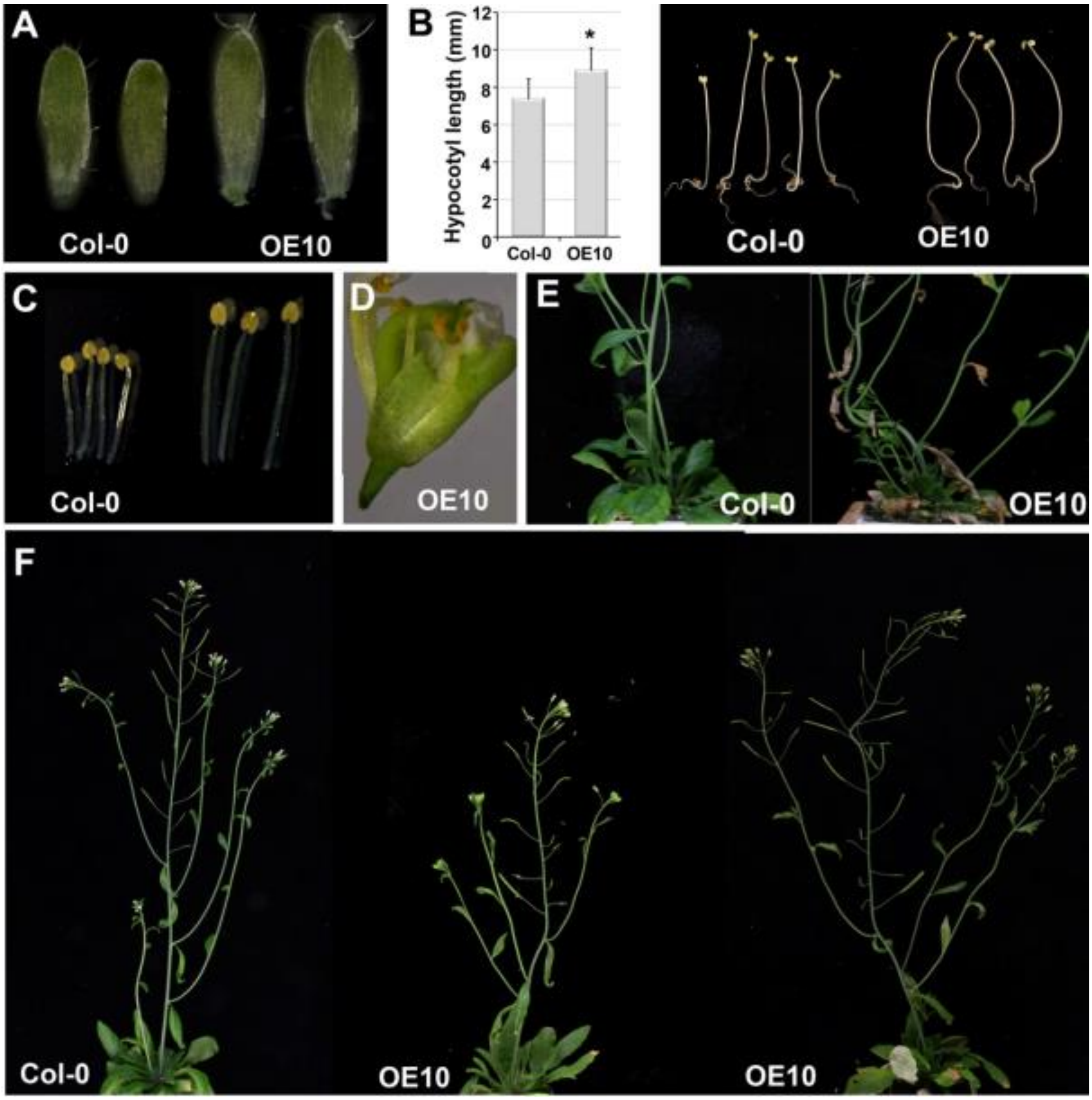

Figure S3. Phenotypes of the SAUR10 overexpression lines. **A)** longer sepals of stage 13 flowers, **B)** longer etiolated hypocotyls at 7 days after stratification, **C)** longer filaments of stage 13 flowers, **D)** very long pistils that often remain unpollinated (maximum silique length is only reached in pollinated pistils), **E)** early senescing rosette and cauline leaves, and **F)** Whole-plant phenotypes of Col-0 and OE10 around 8-10 days after bolting. The overexpression lines display wavy main stems and side branches, show reduced fertility due to the long pistil size, and have an irregular phyllotaxy. Significant differences (t-test, $p<0.05$) are indicated with an asterisk.

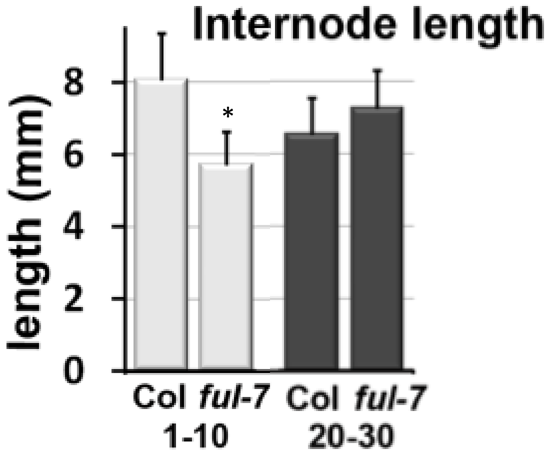

Figure S4. Distance between the silique internodes. *ful-7* plants have a significantly shorter internode distance between the first 10 siliques that appear. Internode distances later in development (e.g. 20-30) are similar to the wild type. Significant differences (t-test, $p<0.05$) are indicated with an asterisk.

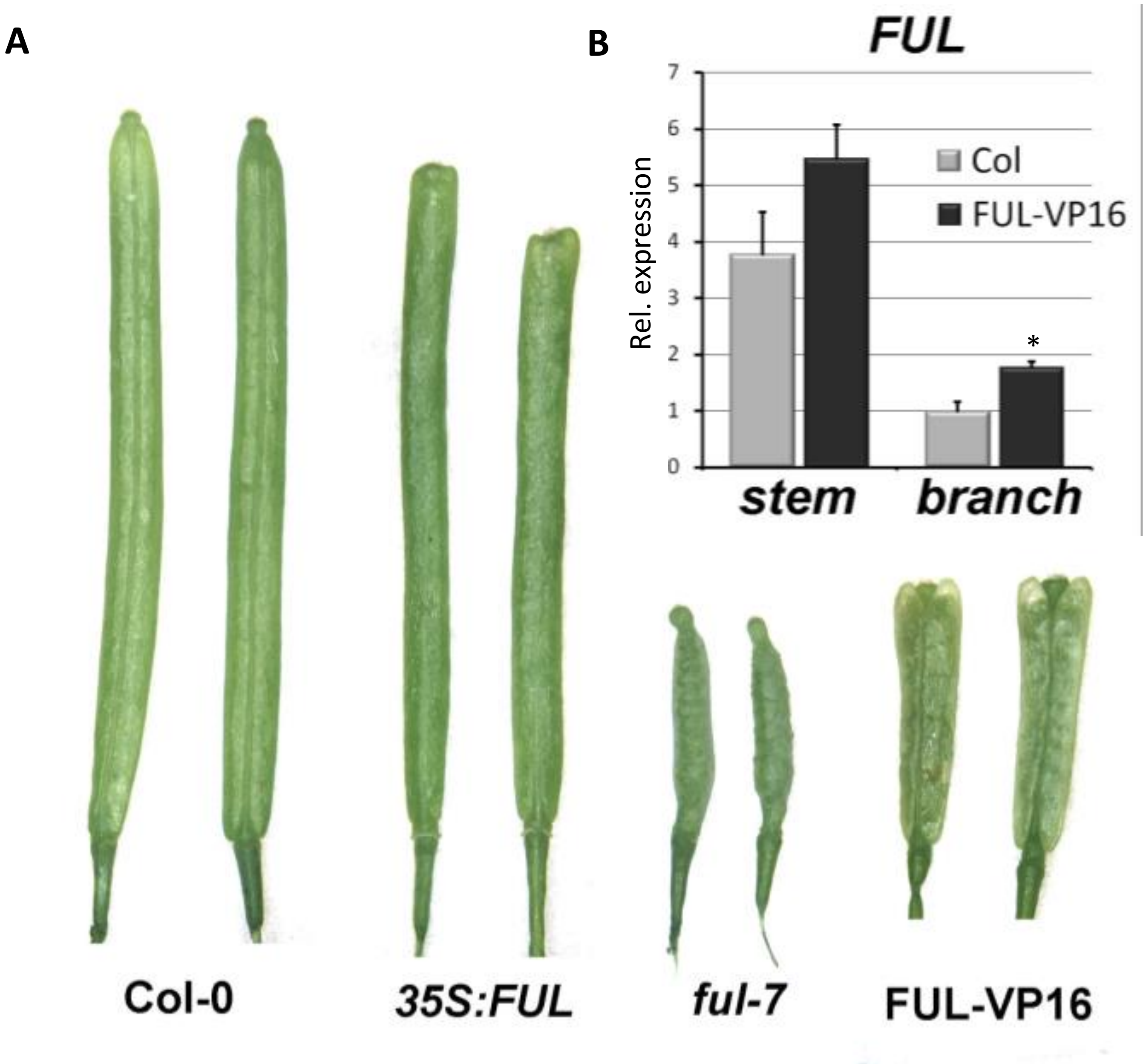

Figure S5. Characterization of the FUL-VP16 plants. A) Siliques stage 17 (Smyth et al., 1990). The apical phenotype of FUL-VP16 siliques resembles 35S:FUL siliques, with distinct 'shoulders' and a short style, but the valve tissues and the overall appearance are more similar to *ful-7* siliques **B)** FUL is 1.5-2 times higher expressed in the FUL-VP16 plants. Stem, 0.5 cm of the stem just below the inflorescence; branch, 0.5 cm of the proximal part of the branch. The expression is depicted relative to the lowest expression (Col-0 in branch). A significant difference from the corresponding Col-0 sample ($p < 0.05$) is indicated with an asterisk.

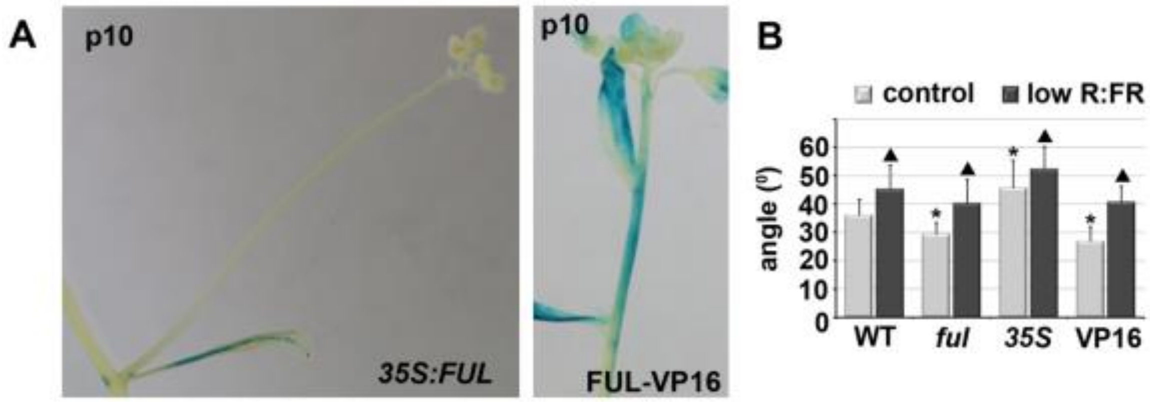

Figure S6. FUL represses *SAUR10* in branches, which can be correlated to branch angle. A) Left panel: in *35S:FUL* branches, *pSAUR10:GUS* signal is only weakly visible or completely absent. Right panel: the pattern in FUL-VP16 branches resembles the pattern in *ful-7* branches with de-repressed *pSAUR10:GUS* just below the inflorescence. **B)** Branch angles are larger in *35S:FUL*, and smaller in *ful-7* and FUL-VP16 lines. Simulated shade resulted in a more horizontal branch growth in all backgrounds. Significant differences from the wild type (t-test, $p<0.05$) are indicated with an asterisk; significant differences from control light conditions in the same background (t-test, $p<0.05$) are indicated with a triangle.

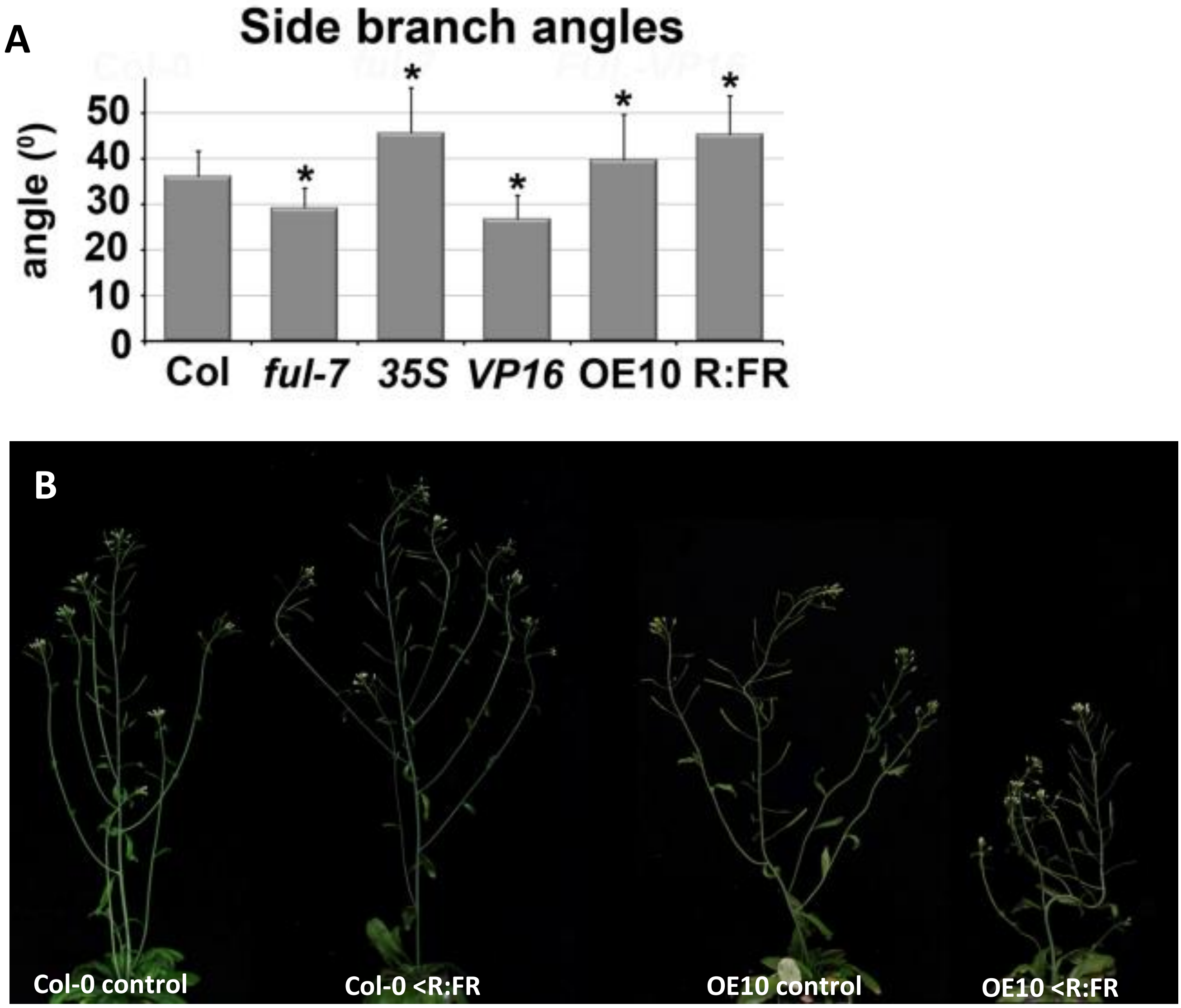

Figure S7. The architecture of Col-0 and *35S:SAUR10* plants changes under reduced R:FR conditions. A) Average branch angle measured in 35S:SAUR10 and under reduced R:FR conditions, compared with the angles in the different FUL backgrounds. B) Architecture phenotypes of Col-0 and 35S:SAUR10 under control conditions and reduced R:FR condtions. Each bar represents the average of at least 20 measured branches. The plants were transferred to reduced R:FR light upon bolting.
