# Supplementary material for: FRUITFULL controls SAUR10 expression and regulates Arabidopsis growth and architecture
Source: J Exp Bot. 2017 Jun 6;68(13):3391–403. doi: 10.1093/jxb/erx184 (PMC5853401; doi:10.1093/jxb/erx184)
Supplement: Supplementary Tables S1-S2 [file erx184_suppl_supplementary_tables_s1-s2.pdf]

Table S1. Identified loci with significant enrichment in the FUL ChIP-Seq

The data presented here are the average from two independent replicas, which were corrected for technical bias using the input sample. ORFs within 3kb downstream to 1 kb upstream of the peak are listed as putative targets, thus allowing multiple putative targets per peak. The genes closest to the peak are considered as most likely targets and were compared with the list of SEP3 targets (according to Kaufmann et al., 2009).

Peak	Locus	SEP3 target	max3kb1kb	u3000	u2000	u1000	d0	d1000	
1	AT1G01080	N	6.15	4.43	0	6.15	0	3.4	33 kDa ribonucleoprotein, chloroplast, putative / RNA-binding protein cp33, putative;
2	AT1G01300		8.14	5	0	0	8.14	5.57	aspartyl protease family protein; FUNCTIONS IN: aspartic-type endopeptidase activity; INVOLVED IN: proteolysis; LOCATED IN: m....
	AT1G01305	N	8.14	5	0	8.14	0	5.57	unknown protein; FUNCTIONS IN: molecular_function unknown; INVOLVED IN: biological_process unknown; LOCATED IN: endomembrane
	AT1G01310		8.14	5	8.14	0	5.57	0	allergen V5/Tpx-1-related family protein; FUNCTIONS IN: molecular_function unknown; INVOLVED IN: biological_process unknown
3	AT1G01580	N	12.59	0	12.59	0	0	0	Encodes the low-iron-inducible ferric chelate reductase responsible for reduction of iron at the root surface.
4	AT1G01760	N	13.12	0	0	13.12	0	5	RNA binding / adenosine deaminase; FUNCTIONS IN: RNA binding, adenosine deaminase activity; INVOLVED IN: RNA processing;
	AT1G01770		13.12	3.14	0	0	13.12	0	unknown protein; INVOLVED IN: biological_process unknown; LOCATED IN: cellular_component unknown; EXPRESSED IN: 22 plant stru....
5	AT1G02065	Y	9.55	0	0	6.15	9.55	4.5	Encodes an SBP-box gene, a member of the SPL gene family. Mutants are affected in micro- and megasporogenesis, trichome forma....

6	AT1G03040	N	11.03	0	0	11.03	0	3.4	basic helix-loop-helix (bHLH) family protein; FUNCTIONS IN: transcription factor activity
	AT1G03050		11.03	0	0	11.03	0	0	epsin N-terminal homology (ENTH) domain-containing protein / clathrin assembly protein-related; FUNCTIONS IN: phospholipid binding
7	AT1G03300	Y	44.28	0	0	44.28	0	0	agenet domain-containing protein; FUNCTIONS IN: RNA binding; INVOLVED IN: biological_process unknown;
	AT1G03310		44.28	0	0	44.28	0	0	DBE1 Debranching enzyme1. Chloroplast isoamylase complex
8	AT1G03830	N	5.85	0	5.85	0	0	4.43	guanylate-binding family protein; FUNCTIONS IN: GTP binding, GTPase activity; INVOLVED IN: immune response
	AT1G03840		5.85	3.4	0	0	5.85	4.43	MAGPIE, MGP is a nuclear-localized putative transcription factor with three zinc finger domains. MGP can interact with three proteins....
9	AT1G04000	N	11.67	0	0	0	11.6 7	0	unknown protein; FUNCTIONS IN: molecular_function unknown; INVOLVED IN: biological_process unknown; LOCATED IN: cellular_comp....
10	AT1G04150	N	9.55	0	0	9.55	0	4.18	C2 domain-containing protein; FUNCTIONS IN: molecular_function unknown
11	AT1G04270		13.64	0	0	13.64	0	0	Encodes cytosolic ribosomal protein S15.
	AT1G04280	Y	13.64	0	0	0	13.6 4	0	unknown protein; LOCATED IN: membrane; EXPRESSED IN: leaf; BEST Arabidopsis thaliana protein match is: unknown protein
12	AT1G04310	N	9.55	0	9.55	0	0	0	encodes an ethylene receptor related to bacterial two-component histidine kinases.
13	AT1G04420		6.81	3.4	0	0	6.81	3.4	aldo/keto reductase family protein; FUNCTIONS IN: oxidoreductase activity, aldo-keto reductase activity; INVOLVED IN: oxidati....

	AT1G04425	N	6.81	3.4	0	6.81	0	3.4	other RNA
14	AT1G04985	N	9.55	0	0	0	9.55	0	unknown protein; FUNCTIONS IN: molecular_function unknown; INVOLVED IN: biological_process unknown
15	AT1G05100	Y	23.21	0	0	23.21	0	3.33	member of MEKK subfamily,MAPKKK18, MITOGEN-ACTIVATED PROTEIN KINASE KINASE KINASE 18
16	AT1G05370	N	6.81	0	0	0	6.81	0	FUNCTIONS IN: molecular_function unknown; INVOLVED IN: biological_process unknown; LOCATED IN: cellular_component unknown
17	AT1G05460	N	5.86	0	0	0	5.86	0	Encodes a protein with similarity to RNA helicases. Mutants are defective in post-transcriptional gene silencing.
18	AT1G05710	Y	9.55	0	9.55	0	0	4.43	ethylene-responsive protein, putative; FUNCTIONS IN: transcription factor activity;
19	AT1G06170		13.15	0	13.15	3.4	0	3.14	basic helix-loop-helix (bHLH) family protein; FUNCTIONS IN: transcription factor activity, DNA binding; INVOLVED IN: regulati....
	AT1G06180	Y	13.15	3.4	13.15	0	0	3.4	member of MYB3R- and R2R3- type MYB-encoding genes
20	AT1G06400	N	6.43	0	0	6.43	0	3.4	ARA-2 Small GTPase binding protein
	AT1G06410		6.43	3.4	6.43	0	0	0	TREHALOSE -6-PHOSPHATASE SYNTHASE S7
21	AT1G07040	N	12.59	3.4	0	12.59	0	0	unknown protein
	AT1G07051		12.59	0	12.59	0	0	5.57	Encodes a microRNA of unknown function. MicroRNAs are regulatory RNAs with a mature length of ~21-nucleotides that are proces....
22	AT1G07090	Y	14.22	3.4	14.22	0	5.57	12.59	LIGHT SENSITIVE HYPOCOTYLS 6 (LSH6); FUNCTIONS IN: molecular_function unknown; INVOLVED IN: biological_process unknown; LOCAT....

	AT1G07100		14.22	5.57	14.22	0	0	3.4	pre-tRNA; tRNA-Ile (anticodon: TAT)
23	AT1G07150	Y	6.81	0	6.81	0	0	3.4	member of MEKK subfamily
24	AT1G07430	Y	8.14	0	6.81	4.43	8.14	0	protein phosphatase 2C, putative / PP2C, putative; FUNCTIONS IN: protein serine/threonine phosphatase activity, catalytic act....
25	AT1G07500	N	6.21	0	0	6.21	3.33	0	unknown protein
	AT1G07510		6.21	0	0	6.21	0	0	encodes an FtsH protease that is localized to the mitochondrion
26	AT1G07645	N	8.14	5.57	8.14	0	0	0	DESSICATION-INDUCED 1VOC SUPERFAMILY PROTEIN (ATDSI-1VOC); FUNCTIONS IN: catalytic activity; INVOLVED IN: response to abiotic....
27	AT1G08120	N	12.68	0	0	12.68	0	3.4	ATP binding / catalytic/ protein kinase
28	AT1G08680	N	9.55	9.55	0	0	0	0	AGD14, ARF GAP-LIKE ZINC FINGER-CONTAINING PROTEIN ZIGA4
29	AT1G08980		5.91	0	0	0	5.91	0	AMIDASE1, Encodes an enzyme with similarity to bacterial acylamidohydrolases and exhibits indole-3-acetamide amidohydrolase activity in....
	AT1G08985	N	5.91	0	5.91	0	0	0	DNA binding; FUNCTIONS IN: DNA binding; INVOLVED IN: regulation of transcription, DNA-dependent; LOCATED IN: cellular_compone....
30	AT1G09070	Y	19.95	0	0	0	19.9<br>5	6.81	SRC2, SRC2 specifically binds the peptide PIEPPPHH, and moves from ER to a vacuole fraction where it gets internalized. Involved in....
31	AT1G09520	Y	9.55	0	0	9.55	0	0	protein binding / zinc ion binding; FUNCTIONS IN: protein binding, zinc ion binding; LOCATED IN: chloroplast; EXPRESSED IN: 2....
32	AT1G09570	N	34.73	0	0	34.73	0	0	ELONGATED HYPOCOTYL8 Light-labile cytoplasmic red/far-red light photoreceptor involved in the regulation of photomorphogenesis.

33	AT1G09800	N	9.55	3.4	0	9.55	0	0	tRNA pseudouridine synthase family protein; FUNCTIONS IN: pseudouridine synthase activity; INVOLVED IN: tRNA processing, pseu
34	AT1G09840		6.7	0	0	6.7	0	0	SHAGGY-LIKE PROTEIN KINASE 41 (ATSK41); FUNCTIONS IN: protein serine/threonine kinase activity, protein kinase activity, ATP ....
	AT1G09850	Y	6.7	0	0	6.7	0	0	Arabidopsis thaliana papain-like cysteine peptidase
35	AT1G10160	N	11.03	0	0	0	11.0 3	0	transposable element gene; non-LTR retrotransposon family (LINE), has a 3.9e-39 P-value blast match to GB:AAA67727 reverse tr....
36	AT1G10360		8.55	6.81	8.55	5.57	0	0	Encodes glutathione transferase belonging to the tau class of GSTs. Naming convention according to Wagner et al. (2002).
	AT1G10370	Y	8.55	0	0	8.55	5.57	0	EARLY-RESPONSIVE TO DEHYDRATION 9 (ERD9); FUNCTIONS IN: glutathione transferase activity; INVOLVED IN: response to water depr....
37	AT1G10740		6.81	0	6.81	0	0	0	unknown protein; INVOLVED IN: glycerol biosynthetic process; LOCATED IN: endomembrane system; EXPRESSED IN: 24 plant structur....
	AT1G10745	Y	6.81	0	0	6.81	0	0	Encodes a Maternally expressed gene (MEG) family protein
38	AT1G10960	N	5.85	0	0	5.85	0	0	FERREDOXIN 1 (ATFD1); FUNCTIONS IN: electron carrier activity, iron-sulfur cluster binding, 2 iron, 2 sulfur cluster binding;....
39	AT1G11120	Y	21.31	0	0	0	21.3 1	0	similar to unknown protein [Arabidopsis thaliana] (TAIR:AT1G61170.1); similar to CK25 [Nicotiana tabacum] (GB:ABE01833.1)
40	AT1G11545		99.32	0	99.32	0	0	0	xyloglucan:xyloglucosyl transferase, putative / xyloglucan

	AT1G11550	Y	99.32	0	0	99.32	0	3.85	pre-tRNA; tRNA-Leu (anticodon: TAG)
	AT1G11560		99.32	0	0	99.32	3.85	0	OST3/OST6 family protein; similar to OST3/OST6 family protein [Arabidopsis thaliana]
41	AT1G11740		6.16	0	0	0	6.16	0	ankyrin repeat family protein; FUNCTIONS IN: protein binding; INVOLVED IN: biological_process unknown; LOCATED IN: cellular_c....
	AT1G11750	N	6.16	0	6.16	0	0	0	One of several nuclear-encoded ClpPs (caseinolytic protease). Contains a highly conserved catalytic triad of Ser-type proteas....
42	AT1G11850	Y	6.81	0	0	6.81	0	0	unknown protein; FUNCTIONS IN: molecular_function unknown; INVOLVED IN: biological_process unknown; LOCATED IN: endomembrane ....
43	AT1G12580	Y	6.81	0	0	5.57	0	6.81	Phosphoenolpyruvate carboxylase-related kinase 1 (PEPKR1); FUNCTIONS IN: protein serine/threonine kinase activity, protein ki....
42	AT1G12610	N	6.81	0	0	0	6.81	5.57	DWARF AND DELAYED FLOWERING1, DDF1, Encodes a member of the DREB subfamily A-1 of ERF/AP2 transcription factor family (DDF1). The protein contains one AP2 domain....
44	AT1G13020	N	12.68	0	0	0	12.68	0	Encodes eIF4B2, eukaryotic initiation factor 4B2.
45	AT1G13240	Y	6.81	0	6.81	0	0	0	pre-tRNA; tRNA-Ile (anticodon: AAT)
46	AT1G13260	Y	21.31	4.43	0	21.31	0	5.57	Encodes an AP2/B3 domain transcription factor which is upregulated in response to low temperature.
47	AT1G13350		6.81	0	6.81	0	0	3	protein kinase family protein; FUNCTIONS IN: protein serine/threonine kinase activity, protein kinase activity, ATP binding; ....

	AT1G13360	Y	6.81	0	0	0	6.81	0	unknown protein; FUNCTIONS IN: molecular_function unknown; INVOLVED IN: biological_process unknown; LOCATED IN: cellular_comp
48	AT1G14160	N	8.14	0	0	8.14	0	0	integral membrane family protein; FUNCTIONS IN: molecular_function unknown; INVOLVED IN: biological_process unknown; LOCATED ....
49	AT1G14270	N	18.89	18.89	0	0	0	0	CAAX amino terminal protease family protein;
50	AT1G14580	Y	6.47	0	0	0	6.47	0	zinc finger (C2H2 type) family protein; FUNCTIONS IN: transcription factor activity, zinc ion binding, nucleic acid binding; ....
51	AT1G14720	Y	11.03	10.26	0	0	11.0<br>3	0	XTR2, member of Glycoside Hydrolase Family 16
52	AT1G14920	Y	8.14	3.4	8.14	0	0	0	GIBBERELIC ACID INSENSITIVE, Similar to a putative transcription factor and transcriptional coactivators. Repressor of GA responses and involved in gibber....
53	AT1G15490	N	6.15	0	0	0	6.15	0	hydrolase, alpha/beta fold family protein; FUNCTIONS IN: hydrolase activity; EXPRESSED IN: 23 plant structures; EXPRESSED DUR....
54	AT1G15530	Y	6.81	0	0	0	6.81	4.43	receptor lectin kinase, putative; FUNCTIONS IN: carbohydrate binding, kinase activity; INVOLVED IN: protein amino acid phosph....
55	AT1G15670	Y	6.21	0	0	0	6.21	0	kelch repeat-containing F-box family protein; FUNCTIONS IN: molecular_function unknown; INVOLVED IN: biological_process unkno....
56	AT1G15800	Y	18.99	4.29	0	0	18.9<br>9	0	unknown protein; FUNCTIONS IN: molecular_function unknown; INVOLVED IN: biological_process unknown; LOCATED IN: cellular_comp....

57	AT1G16150	Y	11.03	0	0	11.03	0	4.18	Encodes a cell-wall associated kinase like protein of the receptor-like kinase (RLK) superfamily. Likely involved in Arabido....
58	AT1G16370	N	7.75	0	0	0	7.75	0	ORGANIC CATION/CARNITINE TRANSPORTER 6 (OCT6); FUNCTIONS IN: carbohydrate transmembrane transporter activity, sugar:hydrogen ....
59	AT1G16900	N	6.98	0	0	4.27	6.98	0	curculin-like (mannose-binding) lectin family protein, very low similarity to Ser Thr protein kinase GI:2598067 from (Zea may....
60	AT1G17170		6.14	4.18	0	0	6.14	0	Encodes glutathione transferase belonging to the tau class of GSTs. Naming convention according to Wagner et al. (2002
	AT1G17180	N	6.14	4.18	6.14	0	0	0	Encodes glutathione transferase belonging to the tau class of GSTs. Naming convention according to Wagner et al. (2002).
61	AT1G17230	N	8.71	3.85	0	8.71	0	0	ATP binding / protein binding / protein kinase/ protein serine/threonine kinase/ protein tyrosine kinase; FUNCTIONS IN: prote....
62	AT1G17400	Y	29.21	15.9	29.21	0	0	4.18	similar to unknown protein [Arabidopsis thaliana] (TAIR:AT1G72490.1); similar to Os07g0614400 [Oryza sativa (japonica cultiva....
63	AT1G17940	Y	38.98	0	38.98	0	0	0	similar to unknown protein [Arabidopsis thaliana] (TAIR:AT1G73390.3); similar to unknown protein [Oryza sativa (japonica
62	AT1G17950	N	38.98	38.98	0	0	0	0	putative transcription factor: R2R3-MYB transcription family
64	AT1G18200		6.81	0	0	6.81	0	0	Arabidopsis Rab GTPase homolog A6b (AtRABA6b); FUNCTIONS IN: protein binding, GTP binding, GTPase activity; INVOLVED IN: intr....

	AT1G18210	Y	6.81	4.43	0	0	6.81	0	calcium-binding protein, putative; FUNCTIONS IN: calcium ion binding; INVOLVED IN: biological_process unknown; LOCATED IN: pl....
65	AT1G18415		6.81	0	0	6.81	0	3.4	Potential natural antisense gene, locus overlaps with AT1G18420
	AT1G18440	Y	6.81	0	0	6.81	0	0	peptidyl-tRNA hydrolase family protein; FUNCTIONS IN: aminoacyl-tRNA hydrolase activity; INVOLVED IN: translation; LOCATED IN....
66	AT1G18720		19.45	9.48	0	19.45	0	0	unknown protein; FUNCTIONS IN: molecular_function unknown; INVOLVED IN: biological_process unknown; LOCATED IN: cellular_comp....
	AT1G18730	Y	19.45	0	0	19.45	0	9.48	likely a subunit of the chloroplast NAD(P)H dehydrogenase complex, involved in PSI cyclic electron transport. Located on the ....
	AT1G18740	Y	19.45	19.45	0	0	9.48	3.4	unknown protein; FUNCTIONS IN: molecular_function unknown; INVOLVED IN: biological_process unknown; EXPRESSED IN: 23 plant st....
	AT1G18750		19.45	19.45	9.48	0	0	3.4	AGL65, Encodes a member of the MIKC (MADS box, Keratin binding domain, and C terminal domain containing)family of transcriptional r....
67	AT1G19190	N	9.69	3	7	0	9.69	6.81	hydrolase; FUNCTIONS IN: hydrolase activity; INVOLVED IN: metabolic process; LOCATED IN: cellular_component unknown; EXPRESSE
	AT1G19210	N	9.69	0	0	0	6.81	9.69	encodes a member of the DREB subfamily A-5 of ERF/AP2 transcription factor family. The protein contains one AP2 domain. There....
68	AT1G19300	Y	6.42	0	0	0	6.42	0	The PARVUS/GLZ1 gene encodes a putative family 8 glycosyl transferase that contributes to xylan biosynthesis. Its gene expres....

69	AT1G19330	Y	19.45	14.22	0	0	19.45	6.42	unknown protein; FUNCTIONS IN: molecular_function unknown; INVOLVED IN: biological_process unknown; LOCATED IN: cellular_comp
	AT1G19340	Y	19.45	0	19.45	0	0	14.22	methyltransferase MT-A70 family protein; FUNCTIONS IN: S-adenosylmethionine-dependent methyltransferase activity, methyltrans....
	AT1G19350		19.45	19.45	0	0	14.22	0	Encodes brassinosteroid (BR) signalling protein that accumulates in the nucleus as dephosphorylated form in response to BRs. ....
70	AT1G19480		5.91	0	0	0	5.91	0	HhH-GPD base excision DNA repair family protein; FUNCTIONS IN: catalytic activity; INVOLVED IN: DNA repair, base-excision rep....
	AT1G19485	N	5.91	0	5.91	0	0	0	AT hook motif-containing protein; FUNCTIONS IN: DNA binding; INVOLVED IN: regulation of transcription, DNA-dependent; LOCATED....
71	AT1G19800	Y	7	0	7	0	0	0	Encodes a permease-Like protein involved in lipid transfer from the ER to the chloroplast, more specifically, transfer of pho
72	AT1G19920	Y	12.07	3.4	0	12.07	0	0	encodes a chloroplast form of ATP sulfurylase
	AT1G19930		12.07	0	0	12.07	0	3.4	kelch repeat-containing F-box family protein; FUNCTIONS IN: molecular_function unknown;
73	AT1G20450	N	15.25	3.45	0	0	15.25	3.19	Encodes a gene induced by low temperature and dehydration. Inhibits e.coli growth while overexpressed. Belongs to the dehydri....
74	AT1G20620	Y	8.14	0	0	3.4	6.81	8.14	SENESCENCE2, Catalase, catalyzes the breakdown of hydrogen peroxide (H2O2) into water and oxygen.
	AT1G20630		8.14	6.81	3.13	8.14	0	0	Catalyzes the reduction of hydrogen peroxide using heme group as cofactor. Protects cells from toxicity by H2O2.

75	AT1G20850	Y	8.14	0	0	8.14	0	0	xylem cysteine peptidase 2 (XCP2); FUNCTIONS IN: peptidase activity, cysteine-type peptidase activity; INVOLVED IN: proteolys....
76	AT1G20890	Y	21.31	0	0	21.31	0	0	similar to unknown protein [Arabidopsis thaliana] (TAIR:AT1G76480.1); similar to Fibronectin, type III-like fold [Medicago tr....
77	AT1G21380		11.12	8.14	0	11.12	0	0	VHS domain-containing protein / GAT domain-containing protein; FUNCTIONS IN: protein transporter activity;
	AT1G21400	Y	11.12	11.12	0	0	8.14	0	2-oxoisovalerate dehydrogenase, putative / 3-methyl-2-oxobutanoate dehydrogenase, putative / branched-chain alpha-keto acid d....
78	AT1G21670	Y	21.31	0	0	21.31	0	0	INVOLVED IN: proteolysis; LOCATED IN: cell wall, plant-type cell wall;
79	AT1G21835	Y	6.81	0	3.4	6.81	0	0	Encodes a Plant thionin family protein
80	AT1G22360	Y	5.85	3.47	0	0	5.85	0	UDP-glucosyl transferase 85A2 (AtUGT85A2); FUNCTIONS IN: UDP-glycosyltransferase activity, transferase activity, transferring....
81	AT1G22460	Y	28.5	5.57	0	0	28.5	0	similar to unknown protein [Arabidopsis thaliana] (TAIR:AT1G22460.1); similar to Plant protein family protein, expressed [Ory....
82	AT1G22600	N	6.81	0	0	6.81	0	0	FUNCTIONS IN: molecular_function unknown; INVOLVED IN: biological_process unknown; LOCATED IN: endomembrane system; EXPRESSED....
83	AT1G22770		12.59	0	0	6.81	0	12.59	GIGANTEA, Together with CONSTANTS (CO) and FLOWERING LOCUS T (FT), GIGANTEA promotes flowering under long days in a circadian clock-con....
	AT1G22810	Y	12.59	0	0	12.59	0	0	encodes a member of the DREB subfamily A-5 of ERF/AP2 transcription factor family. The protein contains one AP2 domain. There....

	AT1G22830		12.59	0	0	12.59	0	0	pentatricopeptide (PPR) repeat-containing protein; INVOLVED IN: biological_process unknown; LOCATED IN: cellular_component un....
84	AT1G23020	Y	6.81	0	0	4.43	6.81	0	Encodes a ferric chelate reductase whose transcription is regulated by FIT1. Expressed in the root, shoot, flower and cotyle....
85	AT1G23390	Y	12.59	0	0	0	9.55	12.59	kelch repeat-containing F-box family protein; FUNCTIONS IN: molecular_function unknown; INVOLVED IN: biological_process unkno....
86	AT1G24260	Y	59.09	59.09	0	29.21	0	0	Member of the MADs box transcription factor family. SEP3 is redundant with SEP1 and 2. Flowers of SEP1/2/3 triple mutants sho....
	AT1G24265	Y	59.09	29.21	59.09	0	0	0	similar to unknown protein [Arabidopsis thaliana] (TAIR:AT1G24267.1); similar to bZIP transcription factor bZIP56 [Glycine ma....
87	AT1G24625	Y	27.16	4.43	14.22	0	27.16	0	ZFP7, Encodes a zinc finger protein containing only a single zinc finger.
	AT1G24640		27.16	27.16	0	14.22	0	4.43	transposable element gene; non-LTR retrotransposon family (LINE), has a 4.3e-37 P-value blast match to GB:NP_038602 L1 repeat
88	AT1G25275	N	7	0	0	0	7	3.4	unknown protein; FUNCTIONS IN: molecular_function unknown; INVOLVED IN: biological_process unknown; LOCATED IN: endomembrane ....
89	AT1G25422	N	17.65	4.43	0	17.65	0	0	unknown protein; FUNCTIONS IN: molecular_function unknown; INVOLVED IN: biological_process unknown; LOCATED IN: cellular_comp....
90	AT1G25500		6.81	0	6.81	0	0	0	choline transporter-related; INVOLVED IN: biological_process unknown; LOCATED IN: chloroplast; EXPRESSED IN: 23 plant structu....

	AT1G25510	Y	6.81	0	0	0	6.81	0	aspartyl protease family protein; FUNCTIONS IN: aspartic-type endopeptidase activity; INVOLVED IN: proteolysis; LOCATED IN: e....
91	AT1G25560	Y	8.14	3.4	0	8.14	5.57	6.81	ETHYLENE RESPONSE DNA BINDING FACTOR1, EDF1, Encodes a member of the RAV transcription factor family that contains AP2 and B3 binding domains. Involved in the regulation
92	AT1G27360	Y	7.3	0	0	0	7.3	0	SQUAMOSA PROMOTER-LIKE, In conjunction with SPL10 and SPL2, SPL11 redundantly controls proper development of lateral organs in association with shoot....
93	AT1G27770	N	7.05	0	0	0	7.05	0	Encodes a chloroplast envelope Ca2+-ATPase with an N-terminal autoinhibitor.
94	AT1G27921		11.03	3.4	11.03	0	0	0	Potential natural antisense gene, locus overlaps with AT1G27920
	AT1G27930	Y	11.03	0	0	3.4	11.0<br>3	0	Function unknown. Interacts with eIF3.
95	AT1G28050	Y	15.9	0	0	15.9	0	0	zinc finger (B-box type) family protein;
	AT1G28060		15.9	0	15.9	0	0	0	small nuclear ribonucleoprotein family protein / snRNP family protein; FUNCTIONS IN: molecular_function unknown; INVOLVED IN:....
96	AT1G28370	N	6.75	3	0	0	6.75	3.4	encodes a member of the ERF (ethylene response factor) subfamily B-1 of ERF/AP2 transcription factor family. The protein cont..
97	AT1G29300	N	11.03	0	0	0	11.0<br>3	0	unfertilized embryo sac 1 (UNE1); INVOLVED IN: double fertilization forming a zygote and endosperm; CONTAINS InterPro DOMAIN/....
98	AT1G30825	Y	6.81	3.4	6.81	0	0	4.43	Involved in trichome maturation. mutant displays enlarged trichomes
99	AT1G31940	N	9.55	0	9.55	0	0	4.11	unknown protein; LOCATED IN: plasma membrane; EXPRESSED IN: 22 plant structures; EXPRESSED DURING: 13 growth stages; BEST

										Ara....
100	AT1G32290	N	9.69	0	0	9.69	0	0		unknown protein; FUNCTIONS IN: molecular_function unknown; INVOLVED IN: biological_process unknown; LOCATED IN: cellular_comp....
	AT1G32300		9.69	0	0	0	9.69	0		FAD-binding domain-containing protein; FUNCTIONS IN: oxidoreductase activity, D-arabinono-1,4-lactone oxidase activity, FAD b....
101	AT1G32640	N	7.05	0	0	0	7.05	3.4		JASMONATE INSENSITIVE 1, Encodes a MYC-related transcriptional activator with a typical DNA binding domain of a basic helix-loop-helix leucine zipper ....
102	AT1G33060	Y	40.02	0	0	40.02	0	0		ANAC014; transcription factor; similar to no apical meristem (NAM) family protein [Arabidopsis thaliana]
103	AT1G34420	N	6.81	0	6.81	0	0	4.27		leucine-rich repeat family protein / protein kinase family protein; FUNCTIONS IN: protein binding, protein tyrosine kinase ac....
104	AT1G35710		7.97	0	0	0	7.97	0		leucine-rich repeat transmembrane protein kinase, putative; FUNCTIONS IN: protein binding, protein serine/threonine kinase ac....
	AT1G35720	N	7.97	0	7.97	0	0	0		Encodes a member of the annexin gene family, a diverse, multigene family of calcium-dependent, membrane-binding proteins. The....
105	AT1G35770		35.58	4.43	35.58	0	0	0		transposable element gene; similar to Ulp1 protease family protein [Arabidopsis thaliana] (TAIR:AT2G06860.1); similar to Ulp1....
	AT1G35780	Y	35.58	0	0	0	35.5<br>8	0		unknown protein;

106	AT1G35870	N	7.05	0	0	0	7.05	0	transposable element gene; gypsy-like retrotransposon family, has a 1.4e-152 P-value blast match to GB:AAD22153 polyprotein (....
107	AT1G43750	N	6.98	0	0	6.98	0	0	transposable element gene; gypsy-like retrotransposon family (Athila)
108	AT1G44060	N	5.9	0	0	0	5.9	0	transposable element gene; CACTA-like transposase family (Ptta/En/Spm), has a 2.0e-22 P-value blast match to At5g29026.1/8-24....
109	AT1G46768		6.81	0	6.81	0	0	0	encodes a member of the DREB subfamily A-5 of ERF/AP2 transcription factor family (RAP2.1). The protein contains one AP2 doma....
	AT1G46840	Y	6.81	0	6.81	0	0	0	F-box family protein; FUNCTIONS IN: molecular_function unknown; INVOLVED IN: biological_process unknown; LOCATED IN: membrane....
110	AT1G47490	Y	7.05	3.52	0	0	7.05	0	ARABIDOPSIS THALIANA RNA-BINDING PROTEIN 47C (ATRBP47C); FUNCTIONS IN: RNA binding; EXPRESSED IN: stem, flower, cultured cell....
111	AT1G48370	N	14.68	0	0	0	14.68	0	Arabidopsis thaliana metal-nicotianamine transporter YSL4
112	AT1G49230	Y	9.55	0	0	9.55	0	0	zinc finger (C3HC4-type RING finger) family protein; FUNCTIONS IN: protein binding, zinc ion binding; EXPRESSED IN: 17 plant ....
113	AT1G49270	N	5.97	0	0	0	5.97	0	protein kinase family protein; FUNCTIONS IN: protein serine/threonine kinase activity, protein tyrosine kinase activity, prot....
114	AT1G50600		6.81	0	6.81	0	0	0	SCARECROW-LIKE SCL5, Encodes a scarecrow-like protein (SCL5). Member of GRAS gene family.
	AT1G50610	N	6.81	0	0	6.81	0	0	leucine-rich repeat transmembrane protein kinase, putative; FUNCTIONS IN: protein serine/threonine

									kinase activity, kinase ac....
115	AT1G51130	Y	22.42	3.4	0	22.42	0	3.4	similar to unknown protein [Arabidopsis thaliana] (TAIR:AT3G20760.1); similar to Os06g0618000 [Oryza sativa (japonica cultiva....
116	AT1G51170	N	11.03	0	0	0	11.0 3	3.4	protein kinase family protein; FUNCTIONS IN: kinase activity; INVOLVED IN: protein amino acid phosphorylation; EXPRESSED IN: ....
117	AT1G51745	N	9.55	0	0	4.43	9.55	0	FUNCTIONS IN: molecular_function unknown; INVOLVED IN: biological_process unknown; LOCATED IN: cellular_component unknown; EX....
118	AT1G52910	N	12.52	5.57	12.52	0	0	0	unknown protein; FUNCTIONS IN: molecular_function unknown; INVOLVED IN: biological_process unknown; LOCATED IN: endomembrane ....
119	AT1G53000	N	6.81	5.57	0	0	6.81	0	Encodes a putative CMP-KDO (3-deoxy-D-manno-octulosonate) synthetase.
	AT1G53010	N	6.81	0	0	6.81	0	0	zinc finger (C3HC4-type RING finger) family protein; FUNCTIONS IN: protein binding, zinc ion binding; LOCATED IN: endomembran....
120	AT1G53830	Y	7.3	0	0	0	7.3	0	encodes a pectin methylesterase
	AT1G53840		7.3	0	0	7.3	0	0	encodes a pectin methylesterase
121	AT1G54035	N	7.97	4.43	3.52	7.97	5.28	0	pseudogene of epithiospecifier protein
	AT1G54040		7.97	4.43	0	0	7.97	5.28	Epithiospecifier protein, interacts with WRKY53. Involved in pathogen resistance and leaf senescence.
122	AT1G55120	Y	8.2	0	0	8.2	0	0	Encodes a protein with fructan exohydrolase (FEH) activity acting on levan-type fructans (6-FEH, levanase). The enzyme does n....
123	AT1G55510	Y	6.81	0	0	6.81	0	0	branched-chain alpha-keto acid decarboxylase E1

									beta
	AT1G55570		6.81	0	0	0	6.81	0	SKU5 Similar 12 (sks12); FUNCTIONS IN: oxidoreductase activity, copper ion binding; LOCATED IN: endomembrane system; EXPRESSE....
124	AT1G55805	N	14.22	0	0	14.22	0	0	BolA-like family protein; FUNCTIONS IN: transcription regulator activity; INVOLVED IN: biological_process unknown; LOCATED IN
	AT1G55810		14.22	0	0	14.22	0	0	One of the homologous genes predicted to encode proteins with UPRT domains (Uracil phosphoribosyltransferase). Five of these ....
125	AT1G56230	N	21.31	0	0	0	21.3 1	6.81	unknown protein; FUNCTIONS IN: molecular_function unknown; INVOLVED IN: biological_process unknown; LOCATED IN: plasma membra....
126	AT1G56640	N	15.9	4.43	15.9	0	0	0	pseudogene, similar to An2 truncated protein, blastp match of 70% identity and 7.4e-11 P-value to GP|7673092|gb|AAF66731.1|AF....
	AT1G60150		15.9	0	15.9	0	0	0	transposable element gene; pseudogene, similar to putative AP endonuclease/reverse transcriptase, blastp match of 28% identit....
127	AT1G60270	N	17.65	0	0	17.65	0	0	pseudogene, glycosyl hydrolase family 1, contains Pfam PF00232 : Glycosyl hydrolase family 1 domain; TIGRFAM TIGR01233: 6-pho....
128	AT1G61260	N	6.43	0	0	0	6.43	0	unknown protein; FUNCTIONS IN: molecular_function unknown; INVOLVED IN: biological_process unknown; LOCATED IN: endomembrane
129	AT1G61890	Y	19.45	0	0	0	19.4 5	0	MATE efflux family protein; FUNCTIONS IN: antiporter activity, drug transporter activity, transporter activity; INVOLVED IN: ....

130	AT1G62045	N	19.45	4.43	19.45	0	0	0	FUNCTIONS IN: molecular_function unknown; INVOLVED IN: biological_process unknown; LOCATED IN: cellular_component unknown; EX....
	AT1G62050		19.45	0	0	0	19.45	0	FUNCTIONS IN: molecular_function unknown; INVOLVED IN: biological_process unknown; LOCATED IN: apoplast; EXPRESSED IN: 6 plan....
131	AT1G62830	Y	15.9	0	0	15.9	0	0	Encodes a homolog of human Lysine-Specific Demethylase1. Involved in H3K4 methylation of target genes including the flowerin....
132	AT1G62981	N	5.85	0	0	0	5.85	0	unknown protein; FUNCTIONS IN: molecular_function unknown; INVOLVED IN: biological_process unknown; LOCATED IN: endomembrane ....
133	AT1G63690	Y	9.55	0	0	0	9.55	0	protease-associated (PA) domain-containing protein; FUNCTIONS IN: peptidase activity, aspartic-type endopeptidase activity; I....
134	AT1G63850		9.69	4.05	0	0	6.81	9.69	PRLI-interacting factor-related; FUNCTIONS IN: protein binding; INVOLVED IN: biological_process unknown; EXPRESSED IN: 21 pla....
	AT1G63860	N	9.69	0	0	0	9.69	6.81	ATP binding / protein binding / transmembrane receptor; FUNCTIONS IN: protein binding, transmembrane receptor activity, ATP b....
135	AT1G64255	N	6.47	0	0	0	6.47	0	SWIM zinc finger family protein; FUNCTIONS IN: zinc ion binding; INVOLVED IN: biological_process unknown; LOCATED IN: cellula
	AT1G64260	N	6.47	0	6.47	0	0	0	zinc finger protein-related; FUNCTIONS IN: zinc ion binding; INVOLVED IN: biological_process unknown; LOCATED IN: cellular_co....

136	AT1G64390	N	9.55	9.55	0	0	6.5	3.4	Arabidopsis thaliana glycosyl hydrolase 9C2 (AtGH9C2); FUNCTIONS IN: carbohydrate binding, hydrolase activity, hydrolyzing O-....
137	AT1G65720		6.81	0	6.81	0	0	0	unknown protein; FUNCTIONS IN: molecular_function unknown; INVOLVED IN: biological_process unknown; LOCATED IN: endomembrane ....
	AT1G65730	Y	6.81	0	0	0	6.81	0	Arabidopsis thaliana metal-nicotianamine transporter YSL4
138	AT1G66350	N	23.21	23.21	0	0	3.45	0	Negative regulator of GA responses, member of GRAS family of transcription factors. Also belongs to the DELLA proteins that....
	AT1G66360		23.21	23.21	0	0	0	0	C2 domain-containing protein; FUNCTIONS IN: molecular_function unknown; INVOLVED IN: biological_process unknown; LOCATED IN: ....
	AT1G66370		23.21	23.21	0	0	0	0	Encodes a member of the MYB family of transcription factors. Involved in regulation of anthocyanin biosynthesis. Affects the ....
139	AT1G67590	Y	12.59	0	0	12.59	0	0	remorin family protein; FUNCTIONS IN: DNA binding; INVOLVED IN: biological_process unknown; LOCATED IN: nucleus; EXPRESSED IN..
140	AT1G67900	Y	7.97	0	0	7.97	0	0	phototropic-responsive NPH3 family protein; FUNCTIONS IN: protein binding, signal transducer activity; INVOLVED IN: response ....
141	AT1G68720	Y	6.81	0	0	6.81	0	0	Encodes the chloroplastic A-to-I tRNA editing enzyme.
	AT1G68790	Y	6.81	0	6.81	0	0	5.57	LITTLE NUCLEI3 (LINC3); INVOLVED IN: biological_process unknown; LOCATED IN: nucleolus; EXPRESSED IN: 23 plant structures; EX....
	AT1G68795		6.81	0	0	0	6.81	5.57	Member of a large family of putative ligands

									homologous to the Clavata3 gene. Consists of a single exon.
142	AT1G68825	Y	43.34	5	0	0	43.3 4	0	ROTUNDIFOLIA-LIKE15, ROTUNDIFOLIA LIKE 15 (RTFL15); INVOLVED IN: shoot development; EXPRESSED IN: stem, flower, root, leaf; CONTAINS InterPro DOMA....
143	AT1G69530		6.81	0	0	0	6.47	6.81	Member of Alpha-Expansin Gene Family. Naming convention from the Expansin Working Group (Kende et al, Plant Mol Bio). Involv....
	AT1G69540	N	6.81	0	6.81	0	0	6.47	AGL94, Encodes a member of the MIKC (MADS box, Keratin binding domain, and C terminal domain containing)family of transcriptional r....
	AT1G69545		6.81	0	0	0	6.81	6.47	leucine-rich repeat family protein; FUNCTIONS IN: protein binding; INVOLVED IN: biological_process unknown; LOCATED IN: cellu....
144	AT1G69600	N	7.05	0	0	0	7.05	0	Encodes ZFHD1, a member of the zinc finger homeodomain transcriptional factor family. Binds to the 62 bp promoter region of ....
145	AT1G70140	Y	6.81	0	0	6.81	0	0	Encodes a group I formin. Binds to F-actin barbed ends. Has severing actin filaments activity. Binds profilin. Involved in th....
	AT1G70150	Y	6.81	0	6.81	0	0	0	zinc ion binding; FUNCTIONS IN: zinc ion binding; INVOLVED IN: biological_process unknown; LOCATED IN: cellular_component unk....
146	AT1G70270	N	6.43	0	0	6.43	0	0	ATP binding / DNA binding; FUNCTIONS IN: DNA binding, ATP binding; INVOLVED IN: biological_process unknown; LOCATED IN: cellu
147	AT1G70280	Y	6.81	6.81	0	0	0	6.43	NHL repeat-containing protein; FUNCTIONS IN: molecular_function unknown; INVOLVED IN: biological_process unknown; EXPRESSED I

	AT1G70290	Y	6.81	0	6.81	0	0	6.43	Encodes an enzyme putatively involved in trehalose biosynthesis. Though the protein has both trehalose-6-phosphate synthase (....
148	AT1G70600		14.71	0	0	3.65	14.7 1	3.4	structural constituent of ribosome; FUNCTIONS IN: structural constituent of ribosome; INVOLVED IN: translation; LOCATED IN: c....
	AT1G70610	N	14.71	0	3.4	14.71	0	0	ARABIDOPSIS THALIANA TRANSPORTER ASSOCIATED WITH ANTIGEN PROCESSING PROTEIN 1
149	AT1G70730	Y	9.55	0	0	0	9.55	4.43	phosphoglucomutase, cytoplasmic, putative / glucose phosphomutase, putative; FUNCTIONS IN: intramolecular transferase activit....
150	AT1G71120		6.81	0	0	6.81	0	0	Contains lipase signature motif and GDSL domain.
	AT1G71130	Y	6.81	0	0	0	6.81	0	encodes a member of the ERF (ethylene response factor) subfamily B-5 of ERF/AP2 transcription factor family. The protein cont....
	AT1G71140		6.81	0	6.81	0	0	0	MATE efflux family protein; FUNCTIONS IN: drug transporter activity, antiporter activity, transporter activity; INVOLVED IN: ....
151	AT1G71960	Y	12.59	7.75	0	0	12.5 9	0	Encodes a plasma membrane localized ABC transporter involved in abscisic acid transport and responses
152	AT1G72210	Y	17.65	0	0	4.43	17.6 5	0	basic helix-loop-helix (bHLH) family protein (bHLH096); FUNCTIONS IN: transcription factor activity, DNA binding; INVOLVED IN....
153	AT1G72450	Y	11.67	0	0	11.67	0	5.57	JAZ6 transcript levels rise in response to a jasmonate stimulus and a GFP:JAZ6 fusion protein localizes to the nucleus. Appl....
154	AT1G72500	Y	16.77	0	0	3.4	16.7 7	14.22	inter-alpha-trypsin inhibitor heavy chain-related; LOCATED IN: plasma membrane; EXPRESSED IN: 23 plant structures; EXPRESSED ....

155	AT1G72520	Y	9.55	3.4	0	0	9.55	0	lipoxygenase, putative; FUNCTIONS IN: electron carrier activity, oxidoreductase activity, acting on single donors with incorp
	AT1G72710		9.55	5.57	0	0	9.55	0	Encodes a member of the casein kinase 1 protein family that is localized to the cytoplasm and nucleus.
156	AT1G72780		6.81	0	0	0	6.81	0	pre-tRNA; tRNA-Ser (anticodon: AGA)
	AT1G72790	N	6.81	0	6.81	0	0	4.81	hydroxyproline-rich glycoprotein family protein; FUNCTIONS IN: molecular_function unknown; INVOLVED IN: biological_process un....
157	AT1G73080	Y	19.45	0	0	19.45	0	3.4	Encodes a leucine-rich repeat receptor kinase. Functions as a receptor for AtPep1 to amplify innate immunity response to pat....
158	AT1G73370	Y	9.55	4.43	0	9.55	0	0	Encodes a protein with sucrose synthase activity (SUS6).
	AT1G73380		9.55	0	0	9.55	0	4.43	unknown protein; FUNCTIONS IN: molecular_function unknown; INVOLVED IN: biological_process unknown; LOCATED IN: cellular_comp....
159	AT1G73805	Y	14.22	0	14.22	0	0	0	calmodulin binding; FUNCTIONS IN: calmodulin binding; INVOLVED IN: biological_process unknown; LOCATED IN: cellular_component....
160	AT1G74020		21.31	0	0	21.31	0	0	Encodes AtSS-2 strictosidine synthase.
	AT1G74030	Y	21.31	0	0	0	0	21.31	enolase, putative; FUNCTIONS IN: phosphopyruvate hydratase activity;
161	AT1G74160	N	17.92	0	0	0	17.9 2	0	unknown protein; FUNCTIONS IN: molecular_function unknown; INVOLVED IN: biological_process unknown; LOCATED IN: cellular_comp....
162	AT1G74440	Y	8.14	5.57	0	0	8.14	0	unknown protein; FUNCTIONS IN: molecular_function unknown; INVOLVED IN:

									biological_process unknown; LOCATED IN: cellular_comp....
163	AT1G75240	N	11.03	0	6.81	0	0	11.03	ARABIDOPSIS THALIANA HOMEOBOX PROTEIN 33 (AtHB33); FUNCTIONS IN: transcription factor activity, DNA binding; INVOLVED IN: reg
164	AT1G75370		8.14	0	0	0	8.14	0	SEC14 cytosolic factor, putative / phosphatidylinositol transfer-like protein, putative; FUNCTIONS IN: transporter acti
	AT1G75450	Y	8.14	4.43	0	0	8.14	3.4	This gene used to be called AtCKX6. It encodes a protein whose sequence is similar to cytokinin oxidase/dehydrogenase, which ....
165	AT1G75510	Y	9.12	4.43	0	9.12	0	5.05	transcription initiation factor IIF beta subunit (TFIIF-beta) family protein; FUNCTIONS IN: RNA polymerase II transcription f....
166	AT1G75800		13.64	0	0	0	13.6 4	10.26	pathogenesis-related thaumatin family protein; FUNCTIONS IN: molecular_function unknown; INVOLVED IN: response to other organ....
	AT1G75810	Y	13.64	0	13.64	10.26	0	4.43	unknown protein; FUNCTIONS IN: molecular_function unknown; INVOLVED IN: biological_process unknown; LOCATED IN: endomembrane ....
167	AT1G75850	N	14.22	0	0	0	14.2 2	0	VPS35 HOMOLOG B (VPS35B); INVOLVED IN: intracellular protein transport, retrograde transport, endosome to Golgi; LOCATED IN: ....
168	AT1G76100	N	6.14	3.4	0	0	6.14	3.4	One of two Arabidopsis plastocyanin genes. Expressed at 1/10th level of PETE2. Does not respond to increased copper levels an....
169	AT1G76170	Y	11.03	0	8.14	11.03	0	5.3	ATP binding; FUNCTIONS IN: ATP binding; INVOLVED IN: tRNA processing; LOCATED IN: cellular_component unknown; EXPRESSED IN: 2....

170	AT1G76360	Y	8.14	0	3.4	0	8.14	0	protein kinase, putative; FUNCTIONS IN: protein serine/threonine kinase activity, protein kinase activity, kinase activity, A....
171	AT1G77480		9.55	9.55	0	5.91	0	0	nucellin protein, putative; FUNCTIONS IN: aspartic-type endopeptidase activity; INVOLVED IN: proteolysis; EXPRESSED IN: 22 pl....
	AT1G77490	Y	9.55	0	0	5.91	0	9.55	Encodes a chloroplastic thylakoid ascorbate peroxidase tAPX. Ascorbate peroxidases are enzymes that scavenge hydrogen peroxi....
172	AT1G78080	Y	6.81	3.4	5.57	0	6.81	0	Encodes a member of the DREB subfamily A-6 of ERF/AP2 transcription factor family (RAP2.4). The protein contains one AP2 doma....
173	AT1G78130	Y	11.03	0	0	11.03	0	0	unfertilized embryo sac 2 (UNE2); FUNCTIONS IN: carbohydrate transmembrane transporter activity, sugar:hydrogen symporter act....
174	AT1G78680	N	12.59	0	0	12.59	0	0	The Arabidopsis protein AtGGH2 is a gamma-glutamyl hydrolase acting specifically on monoglutamates. The enzyme is involved in....
175	AT1G78790	N	7.39	0	0	7.39	0	0	unknown protein; FUNCTIONS IN: molecular_function unknown; INVOLVED IN: biological_process unknown; LOCATED IN: cellular_comp....
176	AT1G79245	N	14.22	0	0	0	14.2 2	0	pseudogene of unknown protein
177	AT1G79700	Y	9.55	0	0	0	9.55	0	ovule development protein, putative; FUNCTIONS IN: transcription factor activity, DNA binding; INVOLVED IN: organ morphogenes....
178	AT1G80450	N	6.81	0	0	0	6.81	0	VQ motif-containing protein; FUNCTIONS IN: molecular_function unknown; INVOLVED IN: biological_process unknown; LOCATED IN: c....
179	AT1G80610	Y	7.88	0	0	0	7.88	3.4	unknown protein; FUNCTIONS IN: molecular_function unknown; INVOLVED IN:

									biological_process unknown; LOCATED IN: cellular_comp....
180	AT2G01008	N	20.28	0	0	7	19.5 9	20.28	other RNA
181	AT2G01020	N	20.28	19.59	0	20.28	0	0	rRNA; 5SrRNA
182	AT2G01420	Y	9.69	0	0	0	9.69	3.4	PIN4, Encodes a putative auxin efflux carrier that is localized in developing and mature root meristems. It is involved in the mai....
183	AT2G01440	N	6.81	0	0	0	6.81	0	ATP binding / ATP-dependent DNA helicase/ ATP-dependent helicase/ helicase/ nucleic acid binding; FUNCTIONS IN: ATP-dependent....
184	AT2G01660	N	5.6	0	0	0	5.6	0	Encodes a plasmodesmal protein that may be involved in the intercellular movement of molecules through the plasmodesmata. The....
185	AT2G01850	Y	22.04	0	22.04	0	14.2 2	0	XTH27, EXGT-A3 has homology to xyloglucan endotransglucosylases/hydrolases (XTHs). EXGT-A3 plays a role in xyloglucan degradation in the differentiating tracheary elements of rosette leaves.
186	AT2G02180	N	8.14	0	0	8.14	0	0	Necessary for the efficient multiplication of tobamoviruses.
187	AT2G02910	N	12.59	0	0	12.59	0	0	FUNCTIONS IN: molecular_function unknown; INVOLVED IN: biological_process unknown; LOCATED IN: cellular_component unknown; CO....
188	AT2G03040	Y	54.14	3.19	0	54.14	0	0	transmembrane protein-related; INVOLVED IN: transport; LOCATED IN: integral to membrane; EXPRESSED IN: pollen tube; CONTAINS ....
189	AT2G03280		55.11	0	55.11	0	0	0	similar to unknown protein [Arabidopsis thaliana] (TAIR:AT1G14020.1); similar to Os03g0169000 [Oryza sativa (japonica cultiva....

	AT2G03290	Y	55.11	0	0	55.11	0	0		protein transmembrane transporter; similar to transmembrane protein-related [Arabidopsis thaliana] (TAIR:AT2G03040.1); simila....
190	AT2G05280		9.55	0	9.55	0	0	0		transposable element gene; non-LTR retrotransposon family (LINE), has a 9.4e-30 P-value blast match to GB:AAA67727 reverse tr....
	AT2G05290	Y	9.55	0	0	9.55	0	0		transposable element gene; similar to unknown protein [Arabidopsis thaliana] (TAIR:AT2G41570.1); similar to 80C09_29 [Brassic....
191	AT2G05632	N	9.55	5.6	0	0	9.55	0		unknown protein; FUNCTIONS IN: molecular_function unknown; INVOLVED IN: biological_process unknown; LOCATED IN: cellular_comp....
	AT2G05640		9.55	0	0	0	5.6	9.55		transposable element gene; pseudogene, similar to putative helicase, low similarity to SP|Q9UUA2 DNA repair and recombination....
	AT2G05845		9.55	0	0	0	9.55	0		transposable element gene; copia-like retrotransposon family, has a 4.3e-45 P-value blast match to GB:CAA32025 ORF (Ty1_Copia....
192	AT2G13570	Y	12.59	0	0	0	12.5 9	0		NUCLEAR FACTOR Y, SUBUNIT B7 (NF-YB7); FUNCTIONS IN: transcription factor activity; INVOLVED IN: regulation of transcription,....
193	AT2G14210	Y	8.71	3.4	0	8.71	0	0		ANR1, AGL44, MADS box gene, transcription factor
194	AT2G14740		8.14	0	0	0	8.14	0		ARABIDOPSIS THALIANA VACULOLAR SORTING RECEPTOR 3 (ATVSR3); FUNCTIONS IN: calcium ion binding; INVOLVED IN: intracellular pro....
	AT2G14750	Y	8.14	0	8.14	0	0	0		Encodes adenosine-5'-phosphosulfate kinase. Provides activated sulfate for sulfation of secondary metabolites, including the....

195	AT2G15000	N	5.85	0	0	0	5.85	0	unknown protein; FUNCTIONS IN: molecular_function unknown; INVOLVED IN: biological_process unknown; LOCATED IN: chloroplast; ....
196	AT2G16018	N	6.81	0	0	0	6.81	0	unknown protein; FUNCTIONS IN: molecular_function unknown; INVOLVED IN: biological_process unknown; LOCATED IN: cellular_comp....
	AT2G16020		6.81	0	0	0	6.81	0	unknown protein; FUNCTIONS IN: molecular_function unknown; INVOLVED IN: biological_process unknown; LOCATED IN: cellular_comp....
197	AT2G16690	Y	8.14	3.4	0	8.14	0	0	transposable element gene; similar to unknown protein [Arabidopsis thaliana] (TAIR:AT2G41570.1); similar to 80C09_29 [Brassic....
198	AT2G17270	N	8.33	0	0	8.33	0	0	mitochondrial substrate carrier family protein; FUNCTIONS IN: binding; INVOLVED IN: transport; LOCATED IN: mitochondrial inne....
199	AT2G17480	Y	5.97	0	0	0	5.97	0	A member of a large family of seven-transmembrane domain proteins specific to plants, homologs of the barley mildew resistanc....
200	AT2G17787		5.6	0	0	0	5.6	0	unknown protein; FUNCTIONS IN: molecular_function unknown; INVOLVED IN: biological_process unknown; LOCATED IN: cellular_comp
	AT2G17790	N	5.6	0	0	5.6	0	0	VPS35 HOMOLOG A (VPS35A); FUNCTIONS IN: molecular_function unknown; INVOLVED IN: intracellular protein transport, retrograde ....
201	AT2G17840		20.07	20.07	0	0	7.83	3.33	EARLY RESPONSIVE TO DEHYDRATION, Identified as drought-inducible gene by differential hybridization. Upregulated by high light, drought,

									cold and salt stress ....
	AT2G17870	Y	20.07	5.57	20.07	0	0	7.83	Encodes COLD SHOCK DOMAIN PROTEIN 3 (CSP3), involved in the acquisition of freezing tolerance.
	AT2G17880	Y	20.07	0	0	0	20.0 7	7.83	DNAJ heat shock protein, putative; FUNCTIONS IN: heat shock protein binding; INVOLVED IN: protein folding; LOCATED IN: chloro....
202	AT2G18010	Y	25.16	17.65	25.16	0	0	3.4	auxin-responsive family protein; FUNCTIONS IN: molecular_function unknown; INVOLVED IN: response to auxin stimulus; EXPRESSED
203	AT2G18440		6.15	0	0	6.15	4.69	0	Encodes a noncoding RNA, a member of an emerging class of transcripts that lack significant open reading frames and encode RN....
	AT2G18450	Y	6.15	3.14	0	0	6.15	4.69	Nuclear encoded mitochondrial flavoprotein subunit of succinate dehydrogenase complex
204	AT2G18700	N	8.14	0	8.14	0	0	4.43	Encodes an enzyme putatively involved in trehalose biosynthesis. The protein has a trehalose synthase (TPS)-like domain that....
205	AT2G19460	N	17.65	0	0	17.65	0	0	similar to unknown protein [Arabidopsis thaliana] (TAIR:AT5G11970.1); similar to hypothetical protein MtrDRAFT_AC152551g19v1 ....
206	AT2G19806	N	6.81	0	6.81	0	0	0	transposable element gene; Mariner-like transposase family, has a 1.2e-19 P-value blast match to GB:AAC28384 mariner transpos....
207	AT2G21045		61.6	0	0	0	9.55	61.6	INVOLVED IN: aging; EXPRESSED IN: hypocotyl, root; CONTAINS InterPro DOMAIN/s: Rhodanese-like (InterPro:IPR001763); BEST Arab....
	AT2G21060	Y	61.6	5.12	0	61.6	0	9.55	COLD SHOCK DOMAIN PROTEIN 4, glycine-rich protein (AtGRP2b)

208	AT2G21210	Y	6.81	0	6.81	0	0	0	Putative auxin-regulated protein whose expression is downregulated in response to chitin oligomers.
209	AT2G21660	Y	14.71	0	0	0	14.7 1	3.4	Encodes a small glycine-rich RNA binding protein that is part of a negative-feedback loop through which AtGRP7 regulates the ....
	AT2G21680		14.71	3.4	14.71	0	0	0	FUNCTIONS IN: molecular_function unknown; INVOLVED IN: biological_process unknown; LOCATED IN: cellular_component unknown; CO....
210	AT2G22240	N	23.21	23.21	0	0	0	0	Myo-inositol-1-phosphate synthase isoform 2.
211	AT2G22330	Y	12.59	0	12.59	0	6.81	8.14	Encodes a cytochrome P450. Involved in tryptophan metabolism. Converts Trp to indole-3-acetaldoxime (IAOx), a precursor to IA....
	AT2G22340		12.59	0	8.14	0	0	12.59	unknown protein; FUNCTIONS IN: molecular_function unknown; INVOLVED IN: biological_process unknown; LOCATED IN: cellular_comp....
	AT2G22345		12.59	12.59	0	8.14	0	0	Encodes a defensin-like (DEFL) family protein.
	AT2G22350		12.59	12.59	0	8.14	0	0	transposable element gene; similar to RNase H domain-containing protein [Arabidopsis thaliana] (TAIR:AT5G36905.1); similar to....
212	AT2G22450	Y	8.14	0	0	8.14	0	3.4	riboflavin biosynthesis protein, putative; FUNCTIONS IN: 3,4-dihydroxy-2-butanone-4-phosphate synthase activity, GTP cyclohyd....
213	AT2G22500	N	10.68	0	0	0	10.6 8	8.14	Encodes one of the mitochondrial dicarboxylate carriers (DIC): DIC1 (AT2G22500), DIC2 (AT4G24570), DIC3 (AT5G09470).
214	AT2G22530	Y	22.42	5.57	0	22.42	0	10.68	transferase; similar to phosphatidylinositolglycan class O (PIG-O) family protein [Arabidopsis thaliana] (TAIR:AT5G17250.1);

	AT2G22540	Y	22.42	22.42	0	0	5.57	0	SVP (SHORT VEGETATIVE PHASE), Encodes a nuclear protein that acts as a floral repressor and that functions within the thermosensory pathway. SVP represses....
215	AT2G22668	Y	6.81	0	0	6.81	0	0	Encodes a microRNA. MicroRNAs are regulatory RNAs with a mature length of ~21-nucleotides that are processed from hairpin pre....
216	AT2G22670	N	14.22	14.22	6.81	0	0	0	IAA8 (IAA8) gene is auxin inducible.
217	AT2G22990	Y	6.81	0	6.81	0	0	5.57	sinapoylglucose:malate sinapoyltransferase. Catalyzes the formation of sinapoylmalate from sinapoylglucose. Mutants accumulat....
218	AT2G23030	N	7	0	7	0	0	0	encodes a member of SNF1-related protein kinases (SnRK2)
219	AT2G23120	Y	8.14	0	0	0	8.14	4.81	FUNCTIONS IN: molecular_function unknown; INVOLVED IN: biological_process unknown; LOCATED IN: plasma membrane; EXPRESSED IN
220	AT2G23170	Y	68.54	0	0	68.54	3.65	0	encodes an IAA-amido synthase that conjugates Asp and other amino acids to auxin in vitro.
	AT2G23180	Y	68.54	68.54	0	0	0	0	CYP96A, Cytochrome P450
221	AT2G23790	Y	9.55	9.12	0	9.55	0	0	unknown protein; FUNCTIONS IN: molecular_function unknown; INVOLVED IN: biological_process unknown; CONTAINS InterPro DOMAIN/....
	AT2G23808		9.55	9.55	0	0	9.12	3.46	pseudogene of transcriptional factor B3 family protein
222	AT2G24240	Y	12.59	0	0	12.59	0	0	potassium channel tetramerisation domain-containing protein; FUNCTIONS IN: protein binding, voltage-gated potassium channel a....
223	AT2G24360	Y	11.03	0	0	11.03	0	0	serine/threonine/tyrosine kinase, putative; FUNCTIONS IN: protein serine/threonine/tyrosine

									kinase activity, protein tyrosine....
224	AT2G24545		15.9	0	15.9	0	0	0	Potential natural antisense gene, locus overlaps with AT2G24540
	AT2G24550	Y	15.9	3.4	0	15.9	0	0	similar to unknown protein [Arabidopsis thaliana] (TAIR:AT4G31510.1); similar to putative KID-containing protein [Brassica na....
225	AT2G24850	N	6.81	0	0	0	6.81	3.14	Encodes a tyrosine aminotransferase that is responsive to treatment with jasmonic acid.
226	AT2G25697	N	6.81	0	6.81	0	0	0	unknown protein; LOCATED IN: endomembrane system; Has 0 Blast hits to 0 proteins in 0 species (source: NCBI BLink).
227	AT2G26040	Y	6.81	0	6.81	0	0	0	Bet v I allergen family protein; FUNCTIONS IN: molecular_function unknown; INVOLVED IN: biological_process unknown; LOCATED I....
228	AT2G26710	Y	24.24	0	0	0	24.2 4	12.59	Encodes a member of the cytochrome p450 family that serves as a control point between multiple photoreceptor systems and bras....
229	AT2G27390		12.59	0	12.59	0	0	0	proline-rich family protein; FUNCTIONS IN: structural constituent of cell wall; INVOLVED IN: biological_process unknown; LOCA....
	AT2G27395		12.59	0	12.59	0	0	0	pseudogene of cysteine protease-related
	AT2G27400	Y	12.59	0	0	12.59	0	0	Trans-acting siRNA1a primary transcript (TAS1a). Regulated by miR173.
	AT2G27402		12.59	0	0	12.59	0	0	FUNCTIONS IN: molecular_function unknown; INVOLVED IN: biological_process unknown; LOCATED IN: cellular_component unknown; BE...
230	AT2G27550	Y	23.21	0	23.21	0	0	0	ATC (ARABIDOPSIS THALIANA CENTRORADIALIS), encodes a protein similar to TFL1. overexpression leads to similar phenotype as

									TFL1 overexpression. expressed specifically i....
231	AT2G28320	Y	285.25	0	0	285.25	0	31.29	pleckstrin homology (PH) domain-containing protein / lipid-binding START domain-containing protein;
232	AT2G28550	Y	9.55	8.14	0	9.55	0	0	RELATED TO AP2.7 (RAP2.7); FUNCTIONS IN: transcription factor activity, DNA binding; INVOLVED IN: organ morphogenesis, regula....
233	AT2G29090	Y	12.59	0	0	0	12.5 9	0	Encodes a protein with ABA 8'-hydroxylase activity, involved in ABA catabolism. Member of the CYP707A gene family. This gene ....
234	AT2G30368		7.3	0	0	7.3	0	5.57	unknown protein; LOCATED IN: endomembrane system; Has 0 Blast hits to 0 proteins in 0 species (source: NCBI BLink).
	AT2G30370	Y	7.3	0	0	0	7.3	5.57	Encodes a small, potentially secreted protein that acts as an inhibitor of stomatal production though likely not through dire....
235	AT2G30424		9.55	0	9.55	0	0	0	TRICHOMELESS2, MYB-transcription factor, In a tandem repeat with AT2g30432 (TCL1) and AT2g30420 (ETC2)
	AT2G30430	Y	9.55	0	9.55	0	0	0	unknown protein; FUNCTIONS IN: molecular_function unknown; INVOLVED IN: biological_process unknown; LOCATED IN: cellular_comp
236	AT2G30490	Y	6.81	3.4	0	6.81	0	3.4	Encodes a cinnamate-4-hydroxylase. Mutations in this gene impact phenylpropanoid metabolism, growth and development.
237	AT2G30530	Y	14.22	4.18	14.22	0	0	0	similar to unknown protein [Arabidopsis thaliana] (TAIR:AT5G01970.1); similar to Os05g0315200 [Oryza sativa (japonica cultiva....

238	AT2G31230	Y	8.14	0	0	8.14	0	3.4	encodes a member of the ERF (ethylene response factor) subfamily B-3 of ERF/AP2 transcription factor family. The protein cont....
239	AT2G32160	N	6.81	0	6.81	0	0	0	FUNCTIONS IN: molecular_function unknown; INVOLVED IN: biological_process unknown; LOCATED IN: cellular_component unknown; EX....
240	AT2G33040	N	14.71	0	0	14.71	0	0	ATP synthase gamma chain, mitochondrial (ATPC); FUNCTIONS IN: hydrogen ion transporting ATP synthase activity, rotational mec....
	AT2G33050		14.71	0	0	14.71	0	0	Receptor Like Protein 26 (AtRLP26); FUNCTIONS IN: protein binding, kinase activity; INVOLVED IN: signal transduction, defense....
242	AT2G33730		11.24	11.24	0	0	6.71	0	DEAD box RNA helicase, putative; FUNCTIONS IN: helicase activity, nucleic acid binding, ATP binding, ATP-dependent helicase a....
	AT2G33760	N	11.24	6.71	0	0	11.2 4	0	pentatricopeptide (PPR) repeat-containing protein; LOCATED IN: cellular_component unknown; EXPRESSED IN: 23 plant structures;....
241	AT2G33860	Y	25.16	25.16	0	0	0	0	ettin (ett) mutations have pleiotropic effects on Arabidopsis flower development, causing increases in perianth organ number,....
243	AT2G34050		10.26	0	0	10.26	0	0	INVOLVED IN: protein complex assembly; LOCATED IN: mitochondrion, chloroplast; EXPRESSED IN: 19 plant structures; EXPRESSED D....
	AT2G34060	Y	10.26	0	0	0	10.2 6	0	peroxidase, putative; FUNCTIONS IN: electron carrier activity, peroxidase activity, heme binding; INVOLVED IN: response to ox....
244	AT2G34250	N	11.03	0	0	0	11.0 3	0	protein transport protein sec61, putative; FUNCTIONS IN: P-P-bond-hydrolysis-driven

									protein transmembrane transporter activit....
245	AT2G34420		9.55	0	0	0	9.55	0	Photosystem II type I chlorophyll a/b-binding protein
	AT2G34430	N	9.55	0	9.55	0	0	0	Photosystem II type I chlorophyll a/b-binding protein
246	AT2G34655	Y	6.81	0	0	0	6.81	0	unknown protein; FUNCTIONS IN: molecular_function unknown; INVOLVED IN: biological_process unknown; LOCATED IN: endomembrane ....
247	AT2G35850	Y	9.12	0	0	0	9.12	0	unknown protein; FUNCTIONS IN: molecular_function unknown; INVOLVED IN: biological_process unknown; LOCATED IN: endomembrane ....
248	AT2G36270	Y	9.55	3.65	0	0	9.55	0	ABA INSENSITIVE 5, Encodes a member of the basic leucine zipper transcription factor family, involved in ABA signalling during seed maturation a....
249	AT2G37170	Y	12.59	0	12.59	0	0	0	member of the plasma membrane intrinsic protein subfamily PIP2. localizes to the plasma membrane and exhibits water transpo....
	AT2G37180	N	12.59	0	12.59	0	0	3.4	a member of the plasma membrane intrinsic protein PIP2. functions as aquaporin and is involved in dessication.
250	AT2G37940	Y	6.81	0	0	6.81	0	6.81	FUNCTIONS IN: molecular_function unknown; INVOLVED IN: biological_process unknown; EXPRESSED IN: 22 plant structures; EXPRESS....
	AT2G37950	Y	6.81	4.43	0	6.81	0	6.81	zinc finger (C3HC4-type RING finger) family protein; FUNCTIONS IN: protein binding, zinc ion binding; EXPRESSED IN: 10 plant ....

251	AT2G38080	N	15.9	3.4	0	0	7.39	15.9	Encodes a protein with similarity to putative laccase, a member of laccase family (17 members in Arabidopsis). Might be invol....
252	AT2G38330		11.12	0	0	0	11.12	0	MATE efflux family protein; FUNCTIONS IN: drug transporter activity, antiporter activity, transporter activity; INVOLVED IN: ....
	AT2G38350	N	11.12	0	11.12	0	0	0	unknown protein; FUNCTIONS IN: molecular_function unknown; INVOLVED IN: biological_process unknown; LOCATED IN: mitochondrion....
253	AT2G38470	N	8.39	0	0	0	8.39	0	WRKY33, Member of the plant WRKY transcription factor family. Regulates the antagonistic relationship between defense pathways media....
254	AT2G39010	Y	11.12	3.4	0	0	11.12	3.3	PLASMA MEMBRANE INTRINSIC PROTEIN 2E (PIP2E); FUNCTIONS IN: water channel activity; INVOLVED IN: transport, response to nemat....
255	AT2G39250	N	9.55	0	0	9.55	0	0	SCHNARCHZAPFEN, Encodes a AP2 domain transcription factor that can repress flowering. SNZ and its paralogous gene, SCHLAFMUTZE (SMZ), share ....
256	AT2G39990		15.9	0	0	15.9	0	0	translation initiation factor eIF2 p47 subunit homolog
	AT2G40000	Y	15.9	0	0	0	15.9	0	ARABIDOPSIS ORTHOLOG OF SUGAR BEET HS1 PRO-1 2 (HSPRO2); FUNCTIONS IN: molecular_function unknown; INVOLVED IN: response to o....
257	AT2G40250	N	6.81	0	0	0	6.81	0	GDSL-motif lipase/hydrolase family protein; FUNCTIONS IN: hydrolase activity, acting on ester bonds, carboxylesterase activit....

258	AT2G40320	N	8.14	0	0	0	8.14	0	unknown protein; INVOLVED IN: biological_process unknown; CONTAINS InterPro DOMAIN/s: Protein of unknown function DUF231, pla....
259	AT2G40600		9.55	0	0	9.55	0	0	appr-1-p processing enzyme family protein; FUNCTIONS IN: molecular_function unknown; INVOLVED IN: biological_process unknown;....
	AT2G40610	Y	9.55	0	0	0	9.55	0	member of Alpha-Expansin Gene Family. Naming convention from the Expansin Working Group (Kende et al, 2004. Plant Mol Bio).
260	AT2G40810	Y	107.94	0	0	107.94	0	0	AtATG18c (Arabidopsis thaliana homolog of yeast autophagy 18 (ATG18) c); similar to AtATG18d
261	AT2G40970	Y	21.31	0	0	21.31	3.44	0	myb family transcription factor; FUNCTIONS IN: transcription factor activity, DNA binding; INVOLVED IN: regulation of transcr....
262	AT2G41070	Y	9.55	0	0	9.55	0	4.69	Transcription factor homologous to ABI5. Regulates AtEm1 expression by binding directly at the AtEm1 promoter. Located in the
263	AT2G41430	Y	10.68	0	0	3.4	9.55	10.68	EARLY RESPONSIVE TO DEHYDRATION, Encodes hydrophilic protein lacking Cys residues that is expressed in response to drought stress, light stress and treatment ....
	AT2G41440		10.68	3.4	9.55	10.68	0	0	unknown protein; EXPRESSED IN: sperm cell, male gametophyte, pollen tube; EXPRESSED DURING: L mature pollen stage, M germinat....
264	AT2G41630	Y	19.45	0	0	19.45	0	3.33	Encodes the transcription factor TFIIB.
265	AT2G41820	Y	21.31	0	21.31	0	0	0	leucine-rich repeat transmembrane protein kinase, putative; similar to leucine-rich repeat transmembrane protein kinase, puta....
266	AT2G41900	Y	5.85	0	0	5.85	4.42	0	zinc finger (CCCH-type) family protein; FUNCTIONS IN: transcription factor activity, nucleic acid binding; INVOLVED IN: regul....

267	AT2G41940	Y	21.31	21.31	0	7	0	9.55	ZFP8, Encodes a zinc finger protein containing only a single zinc finger.
268	AT2G42730		6.47	0	6.47	0	0	0	F-box family protein; FUNCTIONS IN: zinc ion binding; INVOLVED IN: transcription; LOCATED IN: nucleus; CONTAINS InterPro DOMA....
	AT2G42750	N	6.47	0	0	6.47	0	0	DNAJ heat shock N-terminal domain-containing protein; FUNCTIONS IN: unfolded protein binding, heat shock protein binding; INV
269	AT2G42820		29.21	0	29.21	0	0	4.43	HVA22-LIKE PROTEIN F (HVA22F); FUNCTIONS IN: molecular_function unknown; INVOLVED IN: biological_process unknown; LOCATED IN:....
	AT2G42830	Y	29.21	0	0	29.21	0	8.71	AGAMOUS [AG]-like MADS box protein (AGL5) involved in fruit development (valve margin and dehiscence zone differentiation). A....
270	AT2G42850		12.59	0	8.71	0	0	12.59	member of CYP718
	AT2G42870	Y	12.59	0	12.59	0	3.4	8.71	Encodes PHYTOCHROME RAPIDLY REGULATED1 (PAR1), an atypical basic helix-loop-helix (bHLP) protein. Closely related to PAR2 (A....
	AT2G43010	Y	8.14	0	0	0	8.14	0	PIF4, Isolated as a semidominant mutation defective in red -light responses. Encodes a nuclear localized bHLH protein that interac....
271	AT2G43060	Y	7.05	0	0	0	7.05	0	BHLH1, transcription factor; FUNCTIONS IN: transcription factor activity; INVOLVED IN: regulation of transcription; LOCATED IN: nucl....
272	AT2G43410	N	9.55	0	0	0	9.55	4.43	FPA is a gene that regulates flowering time in Arabidopsis via a pathway that is independent of daylength (the autonomous p....
273	AT2G44070	N	6.81	3.4	6.81	0	0	3.91	GTP binding / translation initiation factor; FUNCTIONS IN: GTP binding, translation initiation factor activity; INVOLVED IN:

274	AT2G44130	Y	10.43	0	3	0	10.43	3.76	kelch repeat-containing F-box family protein; FUNCTIONS IN: molecular_function unknown; INVOLVED IN: biological_process unkno....
	AT2G44140		10.43	0	0	0	10.43	3	autophagy 4a (APG4a); FUNCTIONS IN: peptidase activity; INVOLVED IN: autophagy; LOCATED IN: chloroplast; CONTAINS InterPro DO....
275	AT2G45120	Y	8.55	3.4	0	6.81	0	8.55	zinc finger (C2H2 type) family protein; FUNCTIONS IN: transcription factor activity, zinc ion binding, nucleic acid binding;
	AT2G45135	Y	8.55	6.81	0	0	8.55	0	protein binding / zinc ion binding; FUNCTIONS IN: protein binding, zinc ion binding; INVOLVED IN: biological_process unknown;....
	AT2G45140		8.55	6.81	8.55	0	0	5.57	Encodes a Plant VAMP-Associated protein that localizes to the ER and binds to the sitosterol-binding protein ORP3a. The WFDE
276	AT2G45660	Y	22.77	22.77	0	12.59	3.4	0	SOC1, Controls flowering and is required for CO to promote flowering. It acts downstream of FT. Overexpression of (SOC1) AGL20 supp....
	AT2G45670	Y	22.77	6.73	22.77	0	0	12.59	calcineurin B subunit-related; FUNCTIONS IN: calcium ion binding, acyltransferase activity; INVOLVED IN: metabolic process; E....
	AT2G45680	Y	22.77	0	3.85	6.73	3	22.77	TCP family transcription factor, putative; FUNCTIONS IN: transcription factor activity; INVOLVED IN: regulation of transcript....
	AT2G45685		22.77	12.59	0	22.77	3.4	6.73	Potential natural antisense gene, locus overlaps with AT2G45680
277	AT2G45950		28.28	3.4	28.28	0	0	0	ASK20 (ARABIDOPSIS SKP1-LIKE 20); ubiquitin-protein ligase; similar to ASK21 (ARABIDOPSIS SKP1-LIKE 21), ubiquitin-protein li....

	AT2G45960	Y	28.28	0	0	28.28	0	3.4	PIP1, a member of the plasma membrane intrinsic protein subfamily PIP1. localizes to the plasma membrane and exhibits water transpo....
278	AT2G46000		9.55	3.19	0	9.55	0	3.4	unknown protein; FUNCTIONS IN: molecular_function unknown; INVOLVED IN: biological_process unknown; LOCATED IN: endomembrane ....
	AT2G46020	N	9.55	3.4	0	9.55	3.19	3.14	Encodes a SWI/SNF chromatin remodeling ATPase that upregulates transcription of all three CUC genes and is involved in the fo....
279	AT2G46225		6.81	0	0	6.81	0	0	Encodes a subunit of the WAVE complex. The WAVE complex is required for activation of ARP2/3 complex which functions in actin....
280	AT2G46270	Y	17.65	0	0	17.65	0	0	encodes a bZIP G-box binding protein whose expression is induced by ABA. It has been shown to bind to Adh that contains the G....
281	AT2G46420	Y	19.45	0	4.43	19.45	0	0	similar to unknown protein [Arabidopsis thaliana] (TAIR:AT3G61700.1); similar to uncharacterized plant-specific domain TIGR01....
282	AT2G46510	Y	6.81	0	6.81	0	0	4.74	Encodes a nuclear localized BLH domain containing transcriptional activator involved in response to ABA. Overexpression confe....
	AT2G46520	Y	6.81	4.74	6.81	0	0	6.81	cellular apoptosis susceptibility protein, putative / importin-alpha re-exporter, putative; FUNCTIONS IN: protein transporter....
	AT2G46530	Y	6.81	6.81	0	6.81	0	0	AUXIN RESPONSE FACTOR 11 (ARF11); FUNCTIONS IN: transcription factor activity; INVOLVED IN: response to hormone stimulus, reg....
283	AT2G46660	N	7.29	0	0	0	7.29	0	member of CYP78A

284	AT2G46720	Y	17.65	0	0	0	17.6<br>5	0	Encodes KCS13, a member of the 3-ketoacyl-CoA synthase family involved in the biosynthesis of VLCFA (very long chain fatty ac....
285	AT2G46970	N	33.42	0	33.42	0	0	0	PIL1, encodes a novel Myc-related bHLH transcription factor, which physically associated with APRR1/TOC1 and is a member of PIF3 tr....
286	AT2G47050	N	23.21	3.4	0	0	23.2<br>1	0	invertase/pectin methylesterase inhibitor family protein; FUNCTIONS IN: enzyme inhibitor activity, pectinesterase inhibitor a....
287	AT2G47480		9.55	0	0	9.55	0	5.57	unknown protein; FUNCTIONS IN: molecular_function unknown; INVOLVED IN: biological_process unknown; LOCATED IN: cellular_comp....
	AT2G47485	Y	9.55	5.57	0	9.55	0	0	unknown protein; BEST Arabidopsis thaliana protein match is: unknown protein (TAIR:AT3G62650.2); Has 21 Blast hits to 21 prot....
288	AT2G47760		6.81	0	0	0	6.81	3	ALG3; FUNCTIONS IN: alpha-1,3-mannosyltransferase activity, catalytic activity; INVOLVED IN: protein amino acid glycosylation
	AT2G47770		6.81	3	0	6.81	0	0	Encodes a membrane-bound protein designated AtTSPO (Arabidopsis thaliana TSPO-related). AtTSPO is related to the bacterial ou....
	AT2G47780	Y	6.81	0	6.81	0	0	0	rubber elongation factor (REF) protein-related; FUNCTIONS IN: molecular_function unknown; INVOLVED IN: biological_process unk
289	AT3G02140	Y	8.14	0	0	8.14	0	0	AFP4, Encodes a protein that acts in the nucleus and is an important negative regulator of ABA and salt stress responses, and could....
290	AT3G03030		11	0	0	0	11	0	F-box family protein; FUNCTIONS IN: molecular_function unknown; INVOLVED IN: biological_process unknown; LOCATED IN:

									cellular....
	AT3G03040	N	11	0	11	0	0	0	F-box family protein; FUNCTIONS IN: molecular_function unknown; INVOLVED IN: biological_process unknown; LOCATED IN: cellular....
291	AT3G03150	Y	11.03	11.03	0	9.55	0	0	unknown protein; FUNCTIONS IN: molecular_function unknown; INVOLVED IN: biological_process unknown; LOCATED IN: mitochondrion....
	AT3G03160		9.55	0	0	9.55	0	0	unknown protein; FUNCTIONS IN: molecular_function unknown; INVOLVED IN: biological_process unknown; LOCATED IN: endomembrane ....
292	AT3G03170	N	11.03	9.55	0	11.03	0	0	unknown protein; LOCATED IN: cellular_component unknown; EXPRESSED IN: 11 plant structures; EXPRESSED DURING: 4 anthesis, F m....
	AT3G03180		11.03	9.55	11.03	0	0	0	Got1-like family protein; FUNCTIONS IN: molecular_function unknown; INVOLVED IN: vesicle-mediated transport; LOCATED IN: cell....
293	AT3G03450	Y	12.59	0	12.59	0	0	0	Encodes a DELLA protein, a member of the GRAS superfamily of putative transcription factors. DELLA proteins restrain the cell
	AT3G03456		12.59	0	0	12.59	0	0	unknown protein; LOCATED IN: endomembrane system; Has 0 Blast hits to 0 proteins in 0 species (source: NCBI BLink).
294	AT3G03620	N	41.18	0	0	41.18	0	0	MATE efflux family protein; similar to MATE efflux family protein [Arabidopsis thaliana] (TAIR:AT5G17700.1); similar to unnam....

295	AT3G03680	N	8.14	0	0	0	8.14	0	C2 domain-containing protein; FUNCTIONS IN: molecular_function unknown; INVOLVED IN: biological_process unknown; LOCATED IN: ....
296	AT3G03810		25.16	8.14	25.16	0	0	0	embryo sac development arrest 30 (EDA30); FUNCTIONS IN: molecular_function unknown; INVOLVED IN: N-terminal protein myristoyl....
	AT3G03820	Y	25.16	8.14	0	25.16	0	0	auxin-responsive protein, putative; similar to auxin-responsive protein, putative [Arabidopsis thaliana] (TAIR:AT3G03840.1); ....
297	AT3G03830		8.14	7.63	0	8.14	0	0	auxin-responsive protein, putative; similar to auxin-responsive protein, putative [Arabidopsis thaliana] (TAIR:AT3G03820.1); ....
298	AT3G03840	Y	25.16	25.16	0	8.14	0	7.63	auxin-responsive protein, putative; similar to auxin-responsive protein, putative [Arabidopsis thaliana] (TAIR:AT3G03820.1); ....
	AT3G03845		25.16	25.16	8.14	0	0	7.63	pre-tRNA; tRNA-Trp (anticodon: CCA)
	AT3G03847		25.16	0	0	7.63	0	25.16	auxin-responsive family protein; FUNCTIONS IN: molecular_function unknown; INVOLVED IN: response to auxin stimulus; LOCATED I....
	AT3G03850	Y	25.16	25.16	0	7.63	0	0	auxin-responsive protein, putative; similar to auxin-responsive protein, putative [Arabidopsis thaliana] (TAIR:AT3G03830.1); ....
	AT3G03852		25.16	25.16	7.63	0	0	0	pre-tRNA; tRNA-Trp (anticodon: CCA)
299	AT3G04120		23.21	0	0	3.95	15.9	23.21	encodes cytosolic GADPH (C subunit) involved in the glycolytic pathway but also interacts with H2O2 potentially placing it in....
	AT3G04130		23.21	3.95	0	15.9	0	23.21	pentatricopeptide (PPR) repeat-containing protein; FUNCTIONS IN: molecular_function unknown; INVOLVED IN: biological_process ....
	AT3G04140	Y	23.21	15.9	0	23.21	0	22.77	ankyrin repeat family protein; FUNCTIONS IN: protein binding;

300	AT3G04180		22.77	0	0	0	22.7<br>7	0	germin-like protein, putative; FUNCTIONS IN: manganese ion binding, nutrient reservoir activity; INVOLVED IN: biological_proc....
	AT3G04181	N	22.77	0	0	22.77	0	0	unknown protein; FUNCTIONS IN: molecular_function unknown; INVOLVED IN: biological_process unknown; LOCATED IN: cellular_comp....
301	AT3G04730	Y	8.14	0	0	8.14	0	0	early auxin-induced (IAA16)
	AT3G04920	Y	8.14	0	8.14	0	0	3.29	40S ribosomal protein S24 (RPS24A); FUNCTIONS IN: structural constituent of ribosome, nucleotide binding; INVOLVED IN: transl....
302	AT3G05640	Y	29.21	0	29.21	0	0	0	protein phosphatase 2C, putative / PP2C, putative; FUNCTIONS IN: protein serine/threonine phosphatase activity, catalytic act....
303	AT3G06260		6.81	4.59	6.81	0	0	0	Encodes a protein with putative galacturonosyltransferase activity.
	AT3G06270	N	6.81	0	0	6.81	0	4.59	protein phosphatase 2C, putative / PP2C, putative; FUNCTIONS IN: protein serine/threonine phosphatase activity, catalytic act....
304	AT3G06880		11.03	0	0	11.03	0	0	nucleotide binding; FUNCTIONS IN: nucleotide binding; LOCATED IN: cellular_component unknown; CONTAINS InterPro DOMAIN/s: WD4
	AT3G06890	Y	11.03	0	0	11.03	0	0	unknown protein; FUNCTIONS IN: molecular_function unknown; INVOLVED IN: biological_process unknown; LOCATED IN: cellular_comp....
	AT3G06895		11.03	0	11.03	0	0	0	unknown protein; FUNCTIONS IN: molecular_function unknown; INVOLVED IN: biological_process unknown; Has 2 Blast hits to 2 pro....

305	AT3G07350	Y	9.55	0	9.55	0	0	0	unknown protein; FUNCTIONS IN: molecular_function unknown; INVOLVED IN: biological_process unknown; LOCATED IN: cellular_comp
306	AT3G07740		6.81	0	0	0	6.81	0	encodes a transcriptional adaptor ADA2a that interacts with histone acetyltransferase GCN5 homolog and CBF1
	AT3G08720	Y	6.81	5.57	0	6.81	0	0	Encodes a ribosomal-protein S6 kinase. Gene expression is induced by cold and salt (NaCl). Activation of AtS6k is regulated ....
307	AT3G08940	N	7.39	0	0	7.39	0	0	Lhcb4.2 protein (Lhcb4.2, protein involved in the light harvesting complex of photosystem II
308	AT3G10015	Y	102.17	0	0	6.81	0	102.17	pre-tRNA; tRNA-Leu (anticodon: TAA)
	AT3G10040	Y	102.17	0	0	102.17	0	6.81	transcription factor; similar to transcription factor [Arabidopsis thaliana] (TAIR:AT1G21200.1); similar to unnamed protein p....
309	AT3G10720	Y	6.15	3.4	0	0	6.15	0	pectinesterase, putative; FUNCTIONS IN: pectinesterase activity; INVOLVED IN: cell wall modification; LOCATED IN: cell wall, ...
310	AT3G10985	Y	6.81	0	0	0	6.81	6.81	A senescence-associated gene whose expression is induced in response to treatment with Nep1, a fungal protein that causes nec....
	AT3G11000		6.81	6.81	0	0	6.81	0	LOCATED IN: endomembrane system; EXPRESSED IN: 9 plant structures; EXPRESSED DURING: 6 growth stages; CONTAINS InterPro DOMAI....
311	AT3G11410	Y	19.45	19.45	0	3.4	8.2	0	Encodes protein phosphatase 2C. Negative regulator of ABA signalling. Expressed in seeds during germination. mRNA up-
	AT3G11420	Y	19.45	8.2	0	0	19.45	0	fringe-related protein; FUNCTIONS IN: transferase activity, transferring glycosyl groups; INVOLVED IN: biological_process unk....

312	AT3G11673	N	14.22	0	14.22	5.57	0	0	pseudogene of F-box family protein
	AT3G11680		14.22	0	0	0	14.2 2	5.57	unknown protein; CONTAINS InterPro DOMAIN/s: Uncharacterised protein family UPF0005 (InterPro:IPR006214); BEST Arabidopsis th
313	AT3G11770	N	8.14	7	0	8.14	0	0	nucleic acid binding; FUNCTIONS IN: nucleic acid binding; INVOLVED IN: biological_process unknown; LOCATED IN: plasma membran....
	AT3G12190		8.14	0	8.14	0	0	0	FUNCTIONS IN: molecular_function unknown; INVOLVED IN: biological_process unknown; BEST Arabidopsis thaliana protein match is....
314	AT3G12280	Y	6.7	0	0	6.7	0	0	Encodes a retinoblastoma homologue RETINOBLASTOMA-RELATED protein (RBR or RBR1). RBR controls nuclear proliferation in the fe....
	AT3G12290		6.7	0	0	6.7	0	0	tetrahydrofolate dehydrogenase/cyclohydrolase, putative; FUNCTIONS IN: binding, catalytic activity; INVOLVED IN: folic acid a....
315	AT3G13800	Y	12.59	3.4	0	12.59	0	4.43	metallo-beta-lactamase family protein; FUNCTIONS IN: hydrolase activity, catalytic activity; INVOLVED IN: metabolic process;
316	AT3G14220	Y	8.14	0	0	0	8.14	0	GDSL-motif lipase/hydrolase family protein; FUNCTIONS IN: hydrolase activity, acting on ester bonds, carboxylesterase activit....
318	AT3G14960	Y	6.15	0	0	0	6.15	0	galactosyltransferase family protein; FUNCTIONS IN: transferase activity, transferring hexosyl groups, transferase activity, ...
319	AT3G15200	Y	31.29	31.29	0	9.55	0	0	pentatricopeptide (PPR) repeat-containing protein; INVOLVED IN: biological_process unknown; LOCATED IN: cytosolic ribosome; C....

	AT3G15210	Y	31.29	0	9.55	0	23.6<br>2	31.29	Encodes a member of the ERF (ethylene response factor) subfamily B-1 of ERF/AP2 transcription factor family (ATERF-4). The pr....
320	AT3G15290		6.81	0	6.81	3.19	0	0	3-hydroxybutyryl-CoA dehydrogenase, putative; FUNCTIONS IN: coenzyme binding, oxidoreductase activity, 3-hydroxybutyryl-CoA d....
	AT3G16240	Y	6.81	0	0	0	6.81	0	Delta tonoplast intrinsic protein, functions as a water channel and ammonium (NH3) transporter. Highly expressed in flower, s....
321	AT3G16720	Y	6.16	0	0	0	6.16	0	RING-H2 protein induced after exposure to chitin or inactivated crude cellulase preparations.
322	AT3G17330	Y	46.94	0	0	46.94	0	0	ECT7 (evolutionarily conserved C-terminal region 7); similar to ECT6 (evolutionarily conserved C-terminal region 6) [Arabidop....
323	AT3G17390		16.27	0	0	16.27	0	0	S-adenosylmethionine synthetase
	AT3G17400	Y	16.27	0	0	16.27	0	0	F-box family protein; Identical to Putative F-box protein At3g17400 [Arabidopsis Thaliana] (GB:Q9LUT1); similar to F-box fami....
	AT3G17410		16.27	0	16.27	0	0	0	serine/threonine protein kinase, putative; FUNCTIONS IN: protein serine/threonine kinase activity, protein tyrosine kinase ac....
324	AT3G18217		6.81	0	6.81	0	0	0	Encodes a microRNA that targets several SPL family members, including SPL3,4, and 5. By regulating the expression of SPL3 (an....
	AT3G18220	Y	6.81	0	6.81	0	0	0	phosphatidic acid phosphatase family protein / PAP2 family protein; FUNCTIONS IN: catalytic activity, phosphatidate phosphata...
325	AT3G18420		8.14	0	0	8.14	0	0	tetratricopeptide repeat (TPR)-containing protein; FUNCTIONS IN: binding; INVOLVED IN: biological_process unknown; LOCATED IN....

	AT3G18430	N	8.14	0	0	0	8.14	0	calcium-binding EF hand family protein; FUNCTIONS IN: calcium ion binding; INVOLVED IN: N-terminal protein myristoylation; LO....
326	AT3G18535		18.08	18.08	0	12.59	0	0	Potential natural antisense gene, locus overlaps with AT3G18540
	AT3G18540	Y	18.08	0	0	0	12.5 9	18.08	unknown protein; FUNCTIONS IN: molecular_function unknown; INVOLVED IN: biological_process unknown; LOCATED IN: cellular_comp....
327	AT3G18950		12.59	0	0	12.59	0	3.4	transducin family protein / WD-40 repeat family protein; FUNCTIONS IN: nucleotide binding; INVOLVED IN: biological_process un....
328	AT3G19150	Y	6.15	0	0	0	6.15	0	Kip-related protein (KRP) gene, encodes CDK (cyclin-dependent kinase) inhibitor (CKI), negative regulator of cell division. ....
329	AT3G19290	Y	11.03	4.27	11.03	0	0	0	bZIP transcription factor with specificity for abscisic acid-responsive elements (ABRE). Mediate ABA-dependent stress respons
330	AT3G19540	Y	6.81	0	0	6.81	0	0	unknown protein; FUNCTIONS IN: molecular_function unknown; INVOLVED IN: biological_process unknown; LOCATED IN: plasma membra....
	AT3G19980		6.81	0	0	4.43	6.81	0	Encodes catalytic subunit of serine/threonine protein phosphatase 2A. It can associate with phytochromes A and B in vitro. ....
331	AT3G22275	Y	11.03	6.47	0	11.03	0	0	unknown protein
	AT3G22290		11.03	0	11.03	0	0	6.47	unknown protein; FUNCTIONS IN: molecular_function unknown; INVOLVED IN: biological_process unknown; LOCATED IN: cellular_comp....

	AT3G22310		11.03	11.03	0	0	6.47	0	Sequence similarity ot DEAD-box RNA helicases. Binds RNA and DNA. Involved in drought, salt and cold stress responses.
332	AT3G22436		8.14	0	8.14	0	0	0	unknown protein; Has 0 Blast hits to 0 proteins in 0 species (source: NCBI BLink).
	AT3G22440	Y	8.14	0	0	0	8.14	0	hydroxyproline-rich glycoprotein family protein; FUNCTIONS IN: molecular_function unknown; INVOLVED IN: biological_process un....
333	AT3G24240	N	6.16	0	0	0	6.16	0	leucine-rich repeat transmembrane protein kinase, putative; FUNCTIONS IN: protein binding, protein serine/threonine kinase ac....
334	AT3G24600	N	7.05	0	7.05	0	0	0	INVOLVED IN: antigen processing and presentation; LOCATED IN: MHC class I protein complex, membrane; CONTAINS InterPro DOMAIN....
	AT3G24610		7.05	0	0	0	7.05	0	kelch repeat-containing F-box family protein; FUNCTIONS IN: molecular_function unknown; INVOLVED IN: biological_process unkno....
335	AT3G25715	Y	12.59	0	0	12.59	0	0	pre-tRNA; tRNA-Ile (anticodon: AAT)
	AT3G25716		12.59	0	0	0	12.59	0	unknown protein; FUNCTIONS IN: molecular_function unknown; INVOLVED IN: biological_process unknown; LOCATED IN: cellular_comp....
336	AT3G25727	Y	11.03	0	0	11.03	0	0	unknown protein; FUNCTIONS IN: molecular_function unknown; INVOLVED IN: biological_process unknown; LOCATED IN: cellular_comp....
	AT3G25730	N	11.03	0	11.03	0	0	0	ETHYLENE RESPONSE DNA BINDING FACTOR3, EDF3, AP2 domain-containing transcription factor, putative; FUNCTIONS IN: transcription factor activity; INVOLVED IN: regulation of....

337	AT3G26190	Y	6.81	0	0	0	6.81	0	putative cytochrome P450
338	AT3G26420		7.63	0	0	0	7.63	0	Zinc finger-containing glycine-rich RNA-binding protein. Cold-inducible. Contributes to the enhancement of freezing toleranc....
	AT3G26430		7.63	0	0	0	7.63	0	GDSL-motif lipase/hydrolase family protein; FUNCTIONS IN: hydrolase activity, acting on ester bonds, carboxylesterase activit....
	AT3G26440	Y	7.63	0	0	7.63	0	0	unknown protein; FUNCTIONS IN: molecular_function unknown; INVOLVED IN: biological_process unknown; LOCATED IN: cellular_comp....
339	AT3G27010	Y	15.9	0	15.9	0	0	0	At-TCP20, Belongs to a TCP protein transcription factor family. Members of this family contain a predicted basic-helix-loop-helix domai....
340	AT3G27800	Y	14.22	14.22	9.55	0	0	0	similar to zinc knuckle (CCHC-type) family protein [Arabidopsis thaliana] (TAIR:AT4G05360.1); contains domain SCC1 / RAD21 FA....
341	AT3G30170	N	6.81	0	6.81	0	0	0	transposable element gene; Mutator-like transposase family, has a 3.2e-51 P-value blast match to GB:AAA21566 mudrA of transpo....
342	AT3G41761	N	1071.13	0	6.81	0	259.34	1071.13	other RNA
	AT3G41768		1071.13	6.81	259.34	291.7	347.22	1071.13	rRNA; 18SrRNA
343	AT3G41979	Y	649.94	291.7	347.22	246.19	0	649.94	rRNA; 5SrRNA
344	AT3G44260	N	29.21	0	0	0	29.21	0	CCR4-NOT transcription complex protein, putative; FUNCTIONS IN: ribonuclease activity, nucleic acid binding; INVOLVED IN: res....
345	AT3G44716	N	9.55	0	0	9.55	0	5	unknown protein; FUNCTIONS IN: molecular_function unknown; INVOLVED IN: biological_process unknown; LOCATED IN:

									endomembrane ....
346	AT3G44930	N	6.81	0	0	0	6.81	0	member of Putative Na+/H+ antiporter family
347	AT3G45610	Y	9.55	0	0	9.55	0	0	Dof-type zinc finger domain-containing protein; FUNCTIONS IN: transcription factor activity,
	AT3G45630		9.55	0	9.55	0	0	9.55	RNA recognition motif (RRM)-containing protein; FUNCTIONS IN: protein binding, RNA binding, zinc ion binding, nucleotide bind....
	AT3G45638		9.55	0	0	0	9.55	0	Unknown gene
	AT3G45640	Y	9.55	0	9.55	0	0	0	Encodes a mitogen-activated kinase whose mRNA levels increase in response to touch, cold, salinity stress and chitin oligomer....
348	AT3G46620	Y	7.34	0	0	6.81	7.34	0	zinc finger (C3HC4-type RING finger) family protein; FUNCTIONS IN: protein binding, zinc ion binding; INVOLVED IN: response t....
349	AT3G48515		15.73	0	15.73	0	0	3.4	pre-tRNA; tRNA-Tyr (anticodon: GTA)
	AT3G48520	Y	15.73	0	0	0	15.7<br>3	3.4	member of CYP94B
350	AT3G48690	Y	33.42	0	0	33.42	0	0	Encodes a protein with carboxylesterase whose activity was tested using both pNA and 2,4-D-methyl.
351	AT3G48980	Y	25.16	0	0	0	6.21	25.16	FUNCTIONS IN: molecular_function unknown; INVOLVED IN: biological_process unknown; LOCATED IN: cellular_component unknown; CO....
	AT3G48990		25.16	5.05	0	0	25.1<br>6	6.21	AMP-dependent synthetase and ligase family protein; FUNCTIONS IN: catalytic activity, AMP binding; INVOLVED IN: response to c....
	AT3G49000		25.16	6.21	25.16	0	0	5.05	RNA polymerase III subunit RPC82 family protein; FUNCTIONS IN: DNA-directed RNA polymerase activity, DNA binding; INVOLVED IN....

352	AT3G49790	Y	15.9	0	0	15.9	0	0	ATP binding; similar to ATPP2-A10 (Phloem protein 2-A10), carbohydrate binding [Arabidopsis thaliana] (TAIR:AT1G10150.1); sim....
	AT3G49796		15.9	0	0	15.9	0	0	unknown protein; Has 0 Blast hits to 0 proteins in 0 species (source: NCBI BLink).
	AT3G49950	Y	15.9	0	0	15.9	0	0	scarecrow transcription factor family protein; FUNCTIONS IN: transcription factor activity; INVOLVED IN: regulation of transc....
353	AT3G50260		18.99	0	0	0	18.99	5.57	Encodes a member of the DREB subfamily A-5 of ERF/AP2 transcription factor family. The protein contains one AP2 domain. Invol
	AT3G50270	Y	18.99	0	0	18.99	0	5.57	transferase family protein; FUNCTIONS IN: transferase activity, transferring acyl groups other than amino-acyl groups, transf....
354	AT3G50340	Y	7.75	3.4	0	0	7.75	3.4	unknown protein; FUNCTIONS IN: molecular_function unknown; INVOLVED IN: biological_process unknown; EXPRESSED IN: 22 plant st....
355	AT3G50650	Y	12.59	0	12.59	0	0	0	scarecrow-like transcription factor 7 (SCL7); similar to scarecrow transcription factor family protein [Arabidopsis thaliana]....
356	AT3G50740	Y	17.65	0	0	0	17.65	0	UGT72E1 is an UDPG:coniferyl alcohol glucosyltransferase which specifically glucosylates sinapyl- and coniferyl aldehydes. Th....
357	AT3G50760	Y	6.81	0	0	0	6.81	0	Encodes a protein with putative galacturonosyltransferase activity
358	AT3G51460	N	7.39	0	0	0	7.39	0	Encodes RHD4 (ROOT HAIR DEFECTIVE4), a phosphatidylinositol-4-phosphate phosphatase required for root hair development.
359	AT3G51550	Y	31.29	0	3.45	31.29	0	0	Encodes a synergid-expressed, plasma-membrane localized receptor-like kinase that accumulates asymetrically in the synergid m....

360	AT3G52105	Y	17.65	3.4	0	17.65	0	3	similar to putative protein [Medicago truncatula] (GB:ABE93778.1)
361	AT3G52525	N	11.39	3.4	4.43	0	11.3 9	0	OVATE FAMILY PROTEIN 6 (OFP6); INVOLVED IN: biological_process unknown; CONTAINS InterPro DOMAIN/s: Protein of unknown functi
362	AT3G53130	Y	6.81	3.85	0	0	6.81	0	Lutein-deficient 1 (LUT1) required for lutein biosynthesis, member of the xanthophyll class of carotenoids. Involved in epsil....
	AT3G53140	Y	6.81	3.85	0	0	6.81	0	O-diphenol-O-methyl transferase, putative; FUNCTIONS IN: methyltransferase activity, protein dimerization activity, O-methylt....
317	AT3G53350	Y	6.81	0	0	6.81	0	0	myosin heavy chain-related; INVOLVED IN: biological_process unknown; LOCATED IN: nucleus; EXPRESSED IN: 22 plant structures; ....
	AT3G53360		6.81	0	6.81	0	0	0	pentatricopeptide (PPR) repeat-containing protein; FUNCTIONS IN: molecular_function unknown; INVOLVED IN: biological_process ....
363	AT3G53430		8.14	0	0	8.14	0	5.57	60S ribosomal protein L12 (RPL12B); FUNCTIONS IN: structural constituent of ribosome; INVOLVED IN: translation, ribosome biog....
	AT3G53440	Y	8.14	5.57	0	8.14	0	0	DNA binding; FUNCTIONS IN: DNA binding; INVOLVED IN: biological_process unknown; LOCATED IN: cellular_component unknown; EXPR....
364	AT3G53610	N	6.81	0	0	6.81	0	0	GTPase AtRAB8 (atrab8)
365	AT3G54190		8.14	0	8.14	0	0	3.4	FUNCTIONS IN: molecular_function unknown; INVOLVED IN: biological_process unknown; EXPRESSED IN: 22 plant structures; EXPRESS....

	AT3G54200	N	8.14	3.4	0	0	8.14	0	FUNCTIONS IN: molecular_function unknown; INVOLVED IN: biological_process unknown; LOCATED IN: anchored to plasma membrane, p....
366	AT3G54470		11.03	0	0	11.03	0	0	encodes the bi-functional orotate phosphoribosyltransferase/orotidine-5'-phosphate decarboxylase catalyzing the fifth and six....
	AT3G54480	Y	11.03	4.74	0	0	11.0 3	0	Encodes an SKP1 interacting partner (SKIP5).
367	AT3G55550	N	12.61	4.43	12.61	0	0	0	lectin protein kinase, putative; similar to RLK (RECEPTOR LECTIN KINASE), kinase [Arabidopsis thaliana] (TAIR:AT2G37710.1); s....
368	AT3G55710	Y	10.56	0	0	10.56	0	0	UDP-glucoronosyl/UDP-glucosyl transferase family protein; FUNCTIONS IN: UDP-glycosyltransferase activity, transferase activit....
369	AT3G55970		14.71	14.71	0	0	6.47	0	oxidoreductase, 2OG-Fe(II) oxygenase family protein; FUNCTIONS IN: oxidoreductase activity, iron ion binding; LOCATED IN: cel....
	AT3G55980	Y	14.71	6.47	0	6.81	14.7 1	3.33	SALT-INDUCIBLE ZINC FINGER 1 (SZF1); FUNCTIONS IN: transcription factor activity; INVOLVED IN: response to chitin, regulation....
370	AT3G56190		15.9	4.43	15.9	0	0	0	Encodes one of two alpha-SNAPs (soluble NSF attachment protein) in Arabidopsis
	AT3G56200	Y	15.9	0	0	0	15.9	4.43	Encodes a putative amino acid transporter.
371	AT3G56880	Y	8.14	0	0	0	8.14	0	VQ motif-containing protein; FUNCTIONS IN: molecular_function unknown; INVOLVED IN: biological_process unknown; LOCATED IN: c....
372	AT3G57140	N	32.37	0	0	5	32.3 7	0	SUGAR-DEPENDENT 1-LIKE (SDP1-LIKE); FUNCTIONS IN: GTP binding; INVOLVED IN: metabolic process, lipid metabolic process; LOCAT
373	AT3G57250	N	11.03	0	11.03	0	0	0	emsy N terminus domain-containing protein / ENT domain-containing protein; FUNCTIONS IN: molecular_function unknown; INVOLVED....

374	AT3G57390	Y	15.9	0	0	15.9	0	3.4	AGL18, encodes a MADS-box containing protein likely to be a transcription factor that is expressed in endosperm and developing gamet....
375	AT3G57450	N	6.81	0	0	0	6.81	0	unknown protein; FUNCTIONS IN: molecular_function unknown; INVOLVED IN: biological_process unknown; EXPRESSED IN: 23 plant st....
376	AT3G58080	Y	8.14	6.81	0	0	8.14	5.57	unknown protein; FUNCTIONS IN: molecular_function unknown; INVOLVED IN: biological_process unknown; LOCATED IN: cellular_comp....
377	AT3G58170	N	11.03	0	0	11.03	0	0	Encodes a Bet1/Sft1-like SNARE protein which fully suppresses the temperature-sensitive growth defect in sft1-1 yeas
	AT3G58180		11.03	0	0	0	11.0 3	0	PBS lyase HEAT-like repeat-containing protein; FUNCTIONS IN: lyase activity, binding; INVOLVED IN: biological_process unknown....
378	AT3G58850	Y	6.81	0	6.81	0	0	0	Encodes PHYTOCHROME RAPIDLY REGULATED2 (PAR2), an atypical basic helix-loop-helix (bHLP) protein. Closely related to PAR1 (A....
379	AT3G60110	N	12.59	0	0	12.59	0	3.4	DNA binding; FUNCTIONS IN: DNA binding; EXPRESSED IN: 15 plant structures; EXPRESSED DURING: 8 growth stages; CONTAINS InterP....
380	AT3G60400	Y	16.99	16.99	0	12.59	0	3.4	mitochondrial transcription termination factor-related / mTERF-related; similar to mitochondrial transcription termination fa....
381	AT3G61150	Y	8.14	0	0	0	8.14	0	Encodes a homeobox-leucine zipper family protein belonging to the HD-ZIP IV family.
	AT3G61160		8.14	3.4	8.14	0	0	0	shaggy-related protein kinase beta / ASK-beta (ASK2); FUNCTIONS IN: protein serine/threonine

									kinase activity, protein tyrosin....
382	AT3G61190		9.55	0	0	0	9.55	0	Encodes a protein with a C2 domain that binds to BON1 in yeast two hybrid analyses. Its ability to bind to phospholipids is e....
	AT3G61450	N	9.55	0	0	9.55	0	4.43	syntaxin of plants 73 (SYP73)
383	AT3G61560		15.9	0	0	8.33	0	15.9	reticulon family protein (RTNLB6); INVOLVED IN: biological_process unknown; LOCATED IN: endoplasmic reticulum, plasma membran
	AT3G61570		15.9	0	15.9	0	0	8.33	This gene is predicted to encode a protein that functions as a Golgi apparatus structural component known as a golgin in mamm....
	AT3G61580	Y	15.9	8.33	0	15.9	0	5.43	delta-8 sphingolipid desaturase (SLD1); FUNCTIONS IN: oxidoreductase activity, sphingolipid delta-4 desaturase activity; INVO....
384	AT3G61750	Y	6.81	3.4	0	6.81	0	0	auxin-responsive protein -related; FUNCTIONS IN: dopamine beta-monooxygenase activity; INVOLVED IN: histidine catabolic proce....
	AT3G61755		6.81	0	0	6.81	0	3.4	pre-tRNA; tRNA-Ala (anticodon: CGC)
385	AT3G61820	Y	17.65	0	17.65	0	0	0	aspartyl protease family protein; FUNCTIONS IN: aspartic-type endopeptidase activity; INVOLVED IN: proteolysis; LOCATED IN: p....
386	AT3G61880	Y	14.22	3.4	0	0	14.2 2	0	Encodes a cytochrome p450 monooxygenase. Overexpression of this gene allows fruit growth independently of fertilization. Th....
387	AT3G62090	Y	8.71	0	0	8.71	0	0	encodes a novel Myc-related bHLH transcription factor, which physically associated with APRR1/TOC1 and is a member of PIF3 tr....
388	AT3G62270	Y	8.14	0	0	8.14	0	3.4	anion exchange family protein; FUNCTIONS IN: anion exchanger activity; INVOLVED IN: anion transport; LOCATED IN: integral to ....

389	AT3G62710	Y	6.15	0	6.15	0	0	0	glycosyl hydrolase family 3 protein; FUNCTIONS IN: xylan 1,4-beta-xylosidase activity, hydrolase activity, hydrolyzing O-glyc....
390	AT3G62720		15.9	0	0	0	6.15	15.9	Encodes a protein with xylosyltransferase activity, which is specific for UDP-xylose as donor substrate and for oligosacchari....
	AT3G62730		15.9	0	15.9	0	0	6.15	unknown protein; FUNCTIONS IN: molecular_function unknown; INVOLVED IN: biological_process unknown; LOCATED IN: endomembrane ....
	AT3G62735	N	15.9	6.15	0	15.9	0	0	pre-tRNA; tRNA-Gly (anticodon: CCC)
	AT3G62740		15.9	6.15	0	15.9	0	0	BETA GLUCOSIDASE 7 (BGLU7); FUNCTIONS IN: cation binding, hydrolase activity, hydrolyzing O-glycosyl compounds, catalytic act....
391	AT3G63060		20.56	0	0	20.56	0	4.43	EID1-like 3 (EDL3); EXPRESSED IN: stamen;
	AT3G63070	Y	20.56	0	0	20.56	0	3.19	PWWP domain-containing protein; FUNCTIONS IN: molecular_function unknown;
392	AT3G63120	N	6.81	0	0	6.81	0	3.19	cyclin p1;1 (CYCP1;1); FUNCTIONS IN: cyclin-dependent protein kinase activity; INVOLVED IN: regulation of cell cycle; LOCATED....
	AT3G63130		6.81	3.19	0	6.81	0	5	Encodes a RAN GTPase activating protein involved in nuclear import, cell plate formation and mitotic spindle formation.
393	AT3G63440	N	35.6	0	35.6	0	0	4.43	AtCKX6, This gene used to be called AtCKX7. It encodes a protein whose sequence is similar to cytokinin oxidase/dehydrogenase, which ....
	AT3G63445	Y	35.6	4.43	0	0	35.6	0	Potential natural antisense gene, locus overlaps with AT3G63440
394	AT4G00050	Y	6.81	3.4	0	0	6.81	0	unfertilized embryo sac 10 (UNE10); FUNCTIONS IN: transcription factor activity, DNA binding; INVOLVED IN: double fertilizati....

395	AT4G00360	Y	5.85	0	0	0	5.85	0	Encodes a member of the CYP86A subfamily of cytochrome p450 genes. Expressed at moderate levels in flowers, leaves, roots and....
396	AT4G01060	Y	37.78	0	0	37.78	0	3.4	CPL3, Encodes a Myb-related protein similar to CPC. Involved in epidermal cell differentiation. Mutants have reduced numbers of roo....
397	AT4G01940	Y	17.65	0	3.4	17.65	3.19	0	Encodes a protein containing the NFU domain that may be involved in iron-sulfur cluster assembly.
398	AT4G02320	N	17.45	0	0	0	17.4 5	0	pectinesterase family protein; FUNCTIONS IN: enzyme inhibitor activity, pectinesterase activity; INVOLVED IN: cell wall modif....
399	AT4G02420	N	15.9	5.57	0	15.9	0	0	lectin protein kinase, putative; similar to lectin protein kinase, putative [Arabidopsis thaliana] (TAIR:AT3G53810.1); simila....
	AT4G02430		15.9	0	15.9	0	5.57	0	pre-mRNA splicing factor, putative / SR1 protein, putative; similar to SR1 (splicing factor 2), RNA binding [Arabidopsis thal....
400	AT4G02760	N	6.81	0	0	6.81	5.6	0	FUNCTIONS IN: molecular_function unknown; INVOLVED IN: biological_process unknown; LOCATED IN: cellular_component unknown; CO....
401	AT4G02940	N	8.14	0	0	0	8.14	0	oxidoreductase, 2OG-Fe(II) oxygenase family protein; FUNCTIONS IN: oxidoreductase activity; INVOLVED IN: biological_process u
402	AT4G03070	Y	6.14	0	0	6.14	0	0	Encodes a possible 2-oxoglutarate-dependent dioxygenase that is involved in glucosinolate biosynthesis. The gene is expressed....
403	AT4G03320		5.86	0	0	0	5.86	0	TRANSLOCON AT THE INNER ENVELOPE MEMBRANE OF CHLOROPLASTS 20-IV (tic20-IV); EXPRESSED IN: 17 plant structures; EXPRESSED DURI....
	AT4G03330	N	5.86	0	0	5.86	0	0	member of SYP12 Gene Family

404	AT4G03400	Y	11.19	0	0	11.19	0	0	Encodes a GH3-related gene involved in red light-specific hypocotyl elongation. Analysis of sense and antisense transgenic p....
	AT4G03405		11.19	0	11.19	0	0	0	pre-tRNA; tRNA-Gln (anticodon: CTG)
	AT4G03410		11.19	0	11.19	0	0	0	peroxisomal membrane protein-related; FUNCTIONS IN: molecular_function unknown; INVOLVED IN: biological_process unknown; LOCA....
405	AT4G04925	N	6.81	0	0	0	6.81	0	unknown protein; FUNCTIONS IN: molecular_function unknown; INVOLVED IN: biological_process unknown; LOCATED IN: mitochondrion....
406	AT4G05048	N	23.21	3.4	8.2	23.21	0	0	Encodes a C/D box snoRNA (U49.1). Gb: AJ300655
	AT4G05049		23.21	0	0	0	23.21	8.2	unknown pseudogene
	AT4G05053		23.21	0	23.21	0	0	3.4	pseudogene of ATRCY1 (arginine-rich cyclin)
407	AT4G05320	N	6.81	0	0	0	6.81	0	One of five polyubiquitin genes in A. thaliana. These genes encode the highly conserved 76-amino acid protein ubiquitin that....
408	AT4G06474	N	14.9	12.52	14.9	0	0	0	transposable element gene; retroelement pol polyprotein
	AT4G06477		14.9	0	0	0	10.48	14.9	transposable element gene; gypsy-like retrotransposon family, has a 2.1e-112 P-value blast match to GB:AAD19359 polyprotein (....
409	AT4G06708	N	7.55	0	0	0	7.55	4.1	transposable element gene; gypsy-like retrotransposon family (Athila), has a 3.9e-289 P-value blast match to GB:CAA57397 Athi....
410	AT4G08860	N	19.85	19.85	0	0	0	0	transposable element gene; similar to nucleic acid binding / ribonuclease H [Arabidopsis thaliana] (TAIR:AT2G27870.1); simila....

411	AT4G08949		9.12	3.4	9.12	0	0	0	unknown protein; Has 0 Blast hits to 0 proteins in 0 species (source: NCBI BLink).
	AT4G08950	Y	9.12	0	0	0	9.12	3.4	EXORDIUM (EXO); FUNCTIONS IN: molecular_function unknown; INVOLVED IN: response to brassinosteroid stimulus; LOCATED IN: cell....
412	AT4G10070	N	9.55	0	9.55	3.4	0	0	KH domain-containing protein; FUNCTIONS IN: RNA binding, nucleic acid binding; INVOLVED IN: biological_process unknown; LOCAT....
	AT4G10080		9.55	0	0	0	9.55	0	unknown protein; LOCATED IN: cellular_component unknown; BEST Arabidopsis thaliana protein match is: unknown protein (TAIR:AT....
413	AT4G10200	N	6.81	0	0	6.81	0	0	hAT dimerisation domain-containing protein / transposase-related; FUNCTIONS IN: protein dimerization activity; INVOLVED IN: b....
414	AT4G10507	N	19.45	0	19.45	0	0	0	other RNA
415	AT4G11280	Y	7.58	0	0	0	7.58	0	encodes a a member of the 1-aminocyclopropane-1-carboxylate (ACC) synthase (S-adenosyl-L-methionine methylthioadenosine-lyase....
	AT4G11290		7.58	7.58	0	0	0	0	peroxidase, putative; FUNCTIONS IN: electron carrier activity, peroxidase activity, heme binding; INVOLVED IN: response to ox....
416	AT4G11820	Y	8.91	0	8.91	0	0	0	Embryo defective 2778, Encodes a protein with hydroxymethylglutaryl-CoA synthase activity which was characterized by phenotypical complementation of....
417	AT4G12310	Y	21.31	0	0	0	21.31	3.4	member of CYP706A
418	AT4G12380		6.15	0	0	0	6.15	0	unknown protein; FUNCTIONS IN: molecular_function unknown; INVOLVED IN: biological_process unknown; LOCATED IN:

									cellular_comp....
	AT4G12382	N	6.15	0	0	6.15	0	0	FUNCTIONS IN: molecular_function unknown; INVOLVED IN: biological_process unknown; LOCATED IN: cellular_component unknown; CO....
419	AT4G12730	Y	11.03	0	0	0	11.03	0	AF333971 Arabidopsis thaliana fasciclin-like arabinogalactan-protein 2 (Fla2) mRNA, complete cds
420	AT4G12950	N	14.22	0	0	0	14.22	0	FUNCTIONS IN: molecular_function unknown; LOCATED IN: anchored to membrane; CONTAINS InterPro DOMAIN/s: FAS1 domain (InterPro....
421	AT4G13395	Y	17.65	0	0	0	17.65	0	ROTUNDIFOLIA LIKE 12 (RTFL12); FUNCTIONS IN: molecular_function unknown; INVOLVED IN: shoot development; LOCATED IN: cellular....
422	AT4G13955	N	6.81	3.19	6.81	0	0	0	Encodes a defensin-like (DEFL) family protein
423	AT4G14430	Y	5.91	0	5.91	0	0	0	Encodes a peroxisomal delta3, delta2-enoyl CoA isomerase, involved in unsaturated fatty acid degradation. This enzyme might a....
	AT4G14440		5.91	0	0	0	5.91	0	encodes a cytosolic delta3, delta2-enoyl CoA isomerase, involved in unsaturated fatty acid degradation
	AT4G14450		5.91	0	5.91	0	0	0	unknown protein; LOCATED IN: chloroplast; BEST Arabidopsis thaliana protein match is: unknown protein (TAIR:AT3G23170.1); Has....
424	AT4G14590	Y	8.14	0	0	0	8.14	0	embryo defective 2739 (emb2739); FUNCTIONS IN: molecular_function unknown; INVOLVED IN: embryonic development ending in seed ....

	AT4G14600		8.14	0	8.14	0	0	0	FUNCTIONS IN: molecular_function unknown; INVOLVED IN: biological_process unknown; LOCATED IN: plasma membrane; EXPRESSED IN:....
425	AT4G14730	N	19.45	0	0	19.45	0	3.4	transmembrane protein-related; FUNCTIONS IN: glutamate binding; CONTAINS InterPro DOMAIN/s: Uncharacterised protein family
426	AT4G14900	Y	8.33	0	0	0	8.33	0	hydroxyproline-rich glycoprotein family protein; FUNCTIONS IN: molecular_function unknown; INVOLVED IN: biological_process un....
427	AT4G16650	Y	7.75	0	0	7.75	0	0	unknown protein; FUNCTIONS IN: molecular_function unknown; INVOLVED IN: biological_process unknown; LOCATED IN: Golgi apparat....
428	AT4G16750	Y	11.03	0	0	11.03	0	0	encodes a member of the DREB subfamily A-4 of ERF/AP2 transcription factor family. The protein contains one AP2 domain. There....
429	AT4G17090	N	18.95	6.81	0	0	18.9<br>5	0	Encodes a beta-amylase targeted to the chloroplast. Transgenic BMY8 RNAi lines fail to accumulate maltose during cold shock s....
	AT4G17100		18.95	0	0	0	6.81	18.95	senescence-associated protein-related; FUNCTIONS IN: molecular_function unknown; INVOLVED IN: biological_process unknown; LOC....
430	AT4G17670	Y	6.81	6.81	0	6.81	0	0	unknown protein; EXPRESSED IN: 23 plant structures; EXPRESSED DURING: 13 growth stages.
	AT4G17680	Y	6.81	5.36	0	0	6.81	6.81	protein binding / zinc ion binding; FUNCTIONS IN: protein binding, zinc ion binding; INVOLVED IN: biological_process unknown;....
431	AT4G18160	N	14.71	0	0	14.71	0	0	Encodes AtTPK3 (KCO6), a member of the Arabidopsis thaliana K+ channel family of

									AtTPK/KCO proteins.AtTPK3 is targeted to the....
432	AT4G18280	N	7.3	0	0	0	7.3	0	glycine-rich cell wall protein-related; FUNCTIONS IN: molecular_function unknown; INVOLVED IN: biological_process unknown; LO....
433	AT4G19420	Y	8.78	0	0	8.78	0	0	pectinacetylesterase family protein; FUNCTIONS IN: carboxylesterase activity; INVOLVED IN: biological_process unknown; LOCATE....
434	AT4G22000		6.81	4.5	6.81	0	0	0	unknown protein; FUNCTIONS IN: molecular_function unknown; INVOLVED IN: biological_process unknown; LOCATED IN: cellular_comp....
	AT4G22010	Y	6.81	0	0	6.81	4.5	3.73	SKU5 Similar 4 (sks4); FUNCTIONS IN: oxidoreductase activity, copper ion binding; LOCATED IN: membrane, plant-type cell wall
435	AT4G23090	N	6.43	0	6.43	0	0	0	unknown protein; FUNCTIONS IN: molecular_function unknown; LOCATED IN: cellular_component unknown; Has 5 Blast hits to 5 prot....
436	AT4G23180	N	6.81	0	0	0	6.81	0	Encodes a receptor-like protein kinase. Naming convention from Chen et al 2003 (PMID 14756307)
437	AT4G23290	N	6.81	0	0	0	6.81	0	Encodes a cysteine-rich receptor-like protein kinase.
438	AT4G24370	Y	5.91	5	0	5.91	0	0	unknown protein; FUNCTIONS IN: molecular_function unknown; INVOLVED IN: biological_process unknown; LOCATED IN: cellular_comp....
	AT4G24380		5.91	0	0	5.91	0	5	unknown protein; INVOLVED IN: 10-formyltetrahydrofolate biosynthetic process, folic acid and derivative biosynthetic process;....

439	AT4G24570	N	10.82	0	0	0	10.82	0	Encodes one of the mitochondrial dicarboxylate carriers (DIC): DIC1 (AT2G22500), DIC2 (AT4G24570), DIC3 (AT5G09470).
440	AT4G24910	N	6.14	0	6.14	0	0	0	unknown protein; FUNCTIONS IN: molecular_function unknown; INVOLVED IN: biological_process unknown; LOCATED IN: chloroplast; ....
	AT4G24920		6.14	0	0	0	6.14	0	protein transport protein SEC61 gamma subunit, putative; FUNCTIONS IN: P-P-bond-hydrolysis-driven protein transmembrane trans....
441	AT4G25280	Y	8.55	0	0	8.55	0	0	adenylate kinase family protein; FUNCTIONS IN: nucleobase, nucleoside, nucleotide kinase activity, nucleotide kinase activity....
442	AT4G25470	Y	44.6	33.42	6.81	11.03	44.6	0	Encodes a member of the DREB subfamily A-1 of ERF/AP2 transcription factor family (CBF2). The protein contains one AP2 domain....
	AT4G25490	Y	44.6	0	33.42	4.43	7.88	44.6	Transcriptional activator that binds to the DRE/CRT regulatory element and induces COR (cold-regulated) gene expression incre....
	AT4G25500		44.6	44.6	4.43	33.42	0	0	encodes an arginine/serine-rich splicing factor. transcript is alternatively spliced and is differentially expressed in diffe....
443	AT4G25610	Y	13.65	0	0	0	13.65	0	zinc finger (C2H2 type) family protein; FUNCTIONS IN: zinc ion binding, nucleic acid binding; INVOLVED IN: biological_process....
	AT4G25640		13.65	0	9.55	0	0	13.65	MATE efflux family protein; similar to MATE efflux family protein [Arabidopsis thaliana] (TAIR:AT4G00350.1); similar to unnam....
444	AT4G26690	Y	8.14	4.43	0	8.14	0	0	Glycerophosphoryl diester phosphodiesterase-like protein involved in cell wall cellulose accumulation and pectin linking. Imp....
445	AT4G26780		6.47	0	0	6.47	0	0	unknown function

	AT4G26790		6.47	0	0	0	6.47	0	GDSL-motif lipase/hydrolase family protein; FUNCTIONS IN: hydrolase activity, acting on ester bonds, carboxylesterase activit
	AT4G26795	Y	6.47	0	0	0	6.47	0	Potential natural antisense gene, locus overlaps with AT4G26790
446	AT4G27260	Y	8.14	4.43	8.14	0	0	4.43	encodes an IAA-amido synthase that conjugates Asp and other amino acids to auxin in vitro. Lines carrying insertions in this ....
447	AT4G27520	Y	6.81	0	0	0	6.81	0	plastocyanin-like domain-containing protein; FUNCTIONS IN: electron carrier activity, copper ion binding; LOCATED
	AT4G27530		6.81	3.45	6.81	0	0	0	unknown protein; FUNCTIONS IN: molecular_function unknown; INVOLVED IN: biological_process unknown; LOCATED IN: cellular_comp....
448	AT4G27800	Y	44.6	0	3.52	44.6	0	0	protein phosphatase 2C PPH1 / PP2C PPH1 (PPH1); Identical to Protein phosphatase 2C PPH1 (PPH1) [Arabidopsis Thaliana] (GB:P....
449	AT4G27860		9.55	0	0	0	9.55	8.14	integral membrane family protein; FUNCTIONS IN: molecular_function unknown; INVOLVED IN: biological_process unknown
	AT4G27870	Y	9.55	9.55	0	0	8.14	0	integral membrane family protein; FUNCTIONS IN: molecular_function unknown; INVOLVED IN: biological_process unknown; LOCATED ....
450	AT4G28330	Y	7.05	0	0	7.05	0	0	unknown protein; FUNCTIONS IN: molecular_function unknown; INVOLVED IN: biological_process unknown; LOCATED IN: cellular_comp
451	AT4G28560	Y	35.58	0	0	35.58	0	0	encodes a member of a novel protein family that contains contain a CRIB (for Cdc42/Rac-interactive binding) motif required fo....

	AT4G28570		35.58	0	35.58	0	0	0	alcohol oxidase-related; FUNCTIONS IN: electron carrier activity, oxidoreductase activity, oxidoreductase activity, acting on....
452	AT4G29190	Y	8.14	4.43	8.14	0	0	0	zinc finger (CCCH-type) family protein; FUNCTIONS IN: transcription factor activity; INVOLVED IN: regulation of transcription....
453	AT4G29780		42.15	0	0	0	31.3 1	42.15	unknown protein; LOCATED IN: cellular_component unknown; EXPRESSED IN: 22 plant structures; EXPRESSED DURING: 13 growth stage....
	AT4G29790	N	42.15	31.31	0	42.15	0	0	unknown protein; FUNCTIONS IN: molecular_function unknown; EXPRESSED IN: 24 plant structures; EXPRESSED DURING: 15 growth sta....
454	AT4G29860		9.12	0	0	9.12	0	0	Encodes a WD repeat protein with seven WD repeat motifs, predicted to function in protein-protein interaction. Mutations caus
	AT4G29870	N	9.12	0	0	9.12	0	0	FUNCTIONS IN: molecular_function unknown; INVOLVED IN: biological_process unknown; LOCATED IN: plasma membrane; EXPRESSED IN:....
455	AT4G30190	N	7.75	0	0	0	7.75	3.4	belongs to the P-type ATPase superfamily of cation-transporting ATPases, pumps protons out of the cell, generating a proton g..
456	AT4G30440	N	10.68	0	3.4	0	10.6 8	4.43	UDP-D-glucuronate 4-epimerase
457	AT4G30490	Y	11.95	0	0	11.95	0	0	AFG1-like ATPase family protein; similar to AFG1-like ATPase family protein [Arabidopsis thaliana] (TAIR:AT4G28070.2); simila....
	AT4G30500		11.95	0	0	11.95	0	0	similar to unknown protein [Arabidopsis thaliana] (TAIR:AT2G23940.1); similar to Protein of unknown function DUF788 [Medicago....

458	AT4G31390	N	20.26	0	20.26	0	0	0	ABC1 family protein; Identical to Uncharacterized aarF domain-containing protein kinase At4g31390, chloroplast precursor [Ar....
459	AT4G31520	N	6.81	4.43	0	0	6.81	0	SDA1 family protein; FUNCTIONS IN: binding; INVOLVED IN: biological_process unknown; LOCATED IN: cellular_component unknown; ....
	AT4G31530		6.81	0	6.81	0	0	4.43	binding / catalytic; FUNCTIONS IN: binding, catalytic activity; INVOLVED IN: metabolic process; LOCATED IN: chloroplast strom.
460	AT4G32020	Y	15.9	0	0	0	15.9	12.59	unknown protein; FUNCTIONS IN: molecular_function unknown; INVOLVED IN: biological_process unknown; LOCATED IN: chloroplast; ....
	AT4G32030	Y	15.9	15.9	12.59	0	4.43	0	unknown protein; FUNCTIONS IN: molecular_function unknown; INVOLVED IN: biological_process unknown; LOCATED IN: cellular_comp....
461	AT4G32290	Y	8.33	4.81	0	8.33	0	0	unknown protein; FUNCTIONS IN: molecular_function unknown; INVOLVED IN: biological_process unknown; LOCATED IN: endomembrane ....
	AT4G32295		8.33	0	8.33	0	0	4.81	unknown protein; FUNCTIONS IN: molecular_function unknown; INVOLVED IN: biological_process unknown; LOCATED IN: cellular_comp....
462	AT4G32400	Y	5.85	0	0	0	5.85	0	Encodes a plastidial nucleotide uniport carrier protein required to export newly synthesized adenylates into the cytosol.
463	AT4G33170		8.14	0	8.14	3.4	0	0	pentatricopeptide (PPR) repeat-containing protein; LOCATED IN: chloroplast; EXPRESSED IN: 9 plant structures; EXPRESSED DURIN....

	AT4G33180	Y	8.14	0	3.4	0	8.14	0	hydrolase, alpha/beta fold family protein; FUNCTIONS IN: catalytic activity; EXPRESSED IN: 24 plant structures; EXPRESSED DUR....
464	AT4G33580	Y	6.81	0	3.4	0	6.81	0	BETA CARBONIC ANHYDRASE 5 (BCA5); FUNCTIONS IN: carbonate dehydratase activity, zinc ion binding; INVOLVED IN: carbon utiliza....
465	AT4G34000	Y	12.59	0	0	12.59	0	0	ABF3, ABSCISIC ACID RESPONSIVE ELEMENTS-BINDING FACTOR 3
466	AT4G34131	N	21.31	0	0	21.31	0	0	UGT73B3 (UDP-GLUCOSYL TRANSFERASE 73B3); UDP-glycosyltransferase/ abscisic acid glucosyltransferase/ transferase, transferrin....
467	AT4G34215	N	6.04	0	6.04	0	0	0	Encodes a member of the SGNH-hydrolase superfamily of enzymes. The enzymes of the SGNH-hydrolase superfamily facilitate the
	AT4G34220		6.04	0	0	0	6.04	0	leucine-rich repeat transmembrane protein kinase, putative; FUNCTIONS IN: protein binding, protein serine/threonine kinase ac....
468	AT4G34320	N	6.81	0	0	0	6.81	0	unknown protein; FUNCTIONS IN: molecular_function unknown; INVOLVED IN: N-terminal protein myristoylation; LOCATED IN: cellul....
469	AT4G34710	Y	14.22	0	0	14.22	5.43	3.73	SPE2, encodes a arginine decarboxylase (ADC), a rate-limiting enzyme that catalyzes the first step of polyamine (PA) biosynthesis v....
470	AT4G34800	Y	9.55	4.43	0	9.55	0	0	auxin-responsive family protein; FUNCTIONS IN: molecular_function unknown; INVOLVED IN: response to auxin stimulus; LOCATED I....
471	AT4G35590	N	6.81	0	3.4	0	6.81	6.15	RWP-RK domain-containing protein; FUNCTIONS IN: transcription factor activity; INVOLVED IN: regulation of transcription; LOCA....
	AT4G35610	N	6.81	3.4	0	0	6.15	6.81	zinc finger (C2H2 type) family protein; FUNCTIONS IN: transcription factor activity;

									INVOLVED IN: regulation of transcription....
	AT4G35620	Y	6.81	6.81	6.15	0	0	3.4	Cyclin B2;2 (CYCB2;2); FUNCTIONS IN: cyclin-dependent protein kinase regulator activity; INVOLVED IN: regulation of cell cycl....
472	AT4G35930	N	6.81	0	0	6.81	0	0	FUNCTIONS IN: molecular_function unknown; INVOLVED IN: biological_process unknown; LOCATED IN: cellular_component unknown; EX....
473	AT4G36250	Y	6.81	0	0	0	6.81	0	Encodes a putative aldehyde dehydrogenase. The gene is not responsive to osmotic stress and is expressed constitutively at a ....
474	AT4G36640	N	6.81	6.81	6.47	0	0	0	SEC14 cytosolic factor family protein / phosphoglyceride transfer family protein; FUNCTIONS IN: transporter activity; INVOLVE....
	AT4G36648		6.81	0	0	0	6.81	0	Unknown gene
475	AT4G36710	N	13.65	0	0	3.95	13.65	0	transcription factor; FUNCTIONS IN: transcription factor activity; INVOLVED IN: regulation of transcription; LOCATED IN: ce
	AT4G36720		13.65	3.95	13.65	0	0	0	HVA22-LIKE PROTEIN K (HVA22K); FUNCTIONS IN: molecular_function unknown; INVOLVED IN: biological_process unknown; LOCATED
476	AT4G36920	Y	85.35	85.35	61.6	0	0	17.65	AP2, Encodes a floral homeotic gene, a member of the AP2/EREBP (ethylene responsive element binding protein) class of transcriptio....
477	AT4G36960		8.14	4.43	0	0	8.14	0	RNA recognition motif (RRM)-containing protein; FUNCTIONS IN: RNA binding, nucleotide binding, nucleic acid binding; INVOLVED....
478	AT4G37260	Y	6.15	0	0	0	6.15	3.4	MYB73, Member of the R2R3 factor gene family

479	AT4G37410	N	6.81	0	6.81	0	0	0	CYTOCHROME P450, FAMILY 81, SUBFAMILY F, POLYPEPTIDE 4
	AT4G37730		6.81	0	0	0	6.81	0	Arabidopsis thaliana basic leucine-zipper 7 (AtbZIP7); FUNCTIONS IN: transcription factor activity, DNA binding; INVOLVED IN:....
480	AT4G38430	N	9.48	3.4	0	9.48	5.83	0	Member of the RopGEF (guanine nucleotide exchange factor) family, containing the novel PRONE domain (plant-specific Rop nucle....
481	AT4G38440	Y	11.03	9.48	5.83	0	0	11.03	LOCATED IN: chloroplast; EXPRESSED IN: 21 plant structures; EXPRESSED DURING: 12 growth stages; CONTAINS InterPro DOMAIN/s: R....
	AT4G38470	Y	11.03	0	0	11.03	0	5.57	protein kinase family protein; FUNCTIONS IN: protein serine/threonine/tyrosine kinase activity, protein kinase activity; INVO....
482	AT4G38740	N	8.14	0	8.14	0	0	4.43	Encodes cytosolic cyclophilin ROC1.
483	AT4G38860	Y	125.66	3.4	125.66	0	0	0	auxin-responsive protein, putative; similar to auxin-responsive protein, putative [Arabidopsis thaliana] (TAIR:AT2G21220.1); ....
484	AT4G39040	Y	6.81	0	0	6.81	0	0	RNA binding; FUNCTIONS IN: RNA binding; INVOLVED IN: biological_process unknown; LOCATED IN: chloroplast; EXPRESSED IN: 22 pl....
	AT4G39070	Y	6.81	3.79	0	6.81	0	0	zinc finger (B-box type) family protein; FUNCTIONS IN: transcription factor activity, zinc ion binding; INVOLVED IN: response....
485	AT4G39400	Y	33.42	0	33.42	0	0	0	BIN1, Brassinosteroid insensitive, EEncodes a plasma membrane localized leucine-rich repeat receptor kinase involved in brassinosteroid signal transduction. BRI....

486	AT5G01200	N	6.81	4.18	6.81	0	0	0	myb family transcription factor; FUNCTIONS IN: transcription factor activity, DNA binding; INVOLVED IN: regulation of transcr....
487	AT5G01520	Y	15.9	0	0	15.9	0	4.43	zinc finger (C3HC4-type RING finger) family protein; FUNCTIONS IN: protein binding, zinc ion binding;
488	AT5G01595	Y	9.55	0	0	0	9.55	0	Potential natural antisense gene, locus overlaps with AT5G01600
	AT5G01600	Y	9.55	0	0	0	9.55	0	Encodes a ferretin protein that is targeted to the chloroplast. Member of a Ferritin gene family. Gene expression is induced....
	AT5G01610		9.55	0	9.55	0	0	6.81	unknown protein; INVOLVED IN: biological_process unknown; LOCATED IN: cellular_component unknown; EXPRESSED IN: 20 plant stru....
	AT5G01630		9.55	0	0	6.81	0	9.55	Ortholog of breast cancer susceptibility protein 2. Essential at meiosis. Interacts with with both Rad51 and Dss1(I) or both....
489	AT5G01710	N	8.71	4.43	0	8.71	0	0	LOCATED IN: endomembrane system; EXPRESSED IN: sperm cell, male gametophyte, pollen tube; EXPRESSED DURING: L mature pollen s....
	AT5G01712		8.71	4.43	0	8.71	0	0	Upstream open reading frames (uORFs) are small open reading frames found in the 5' UTR of a mature mRNA, and can potentially ....
	AT5G01715		8.71	0	0	8.71	4.17	4.43	pseudogene, antisense mRNA to gene At5g01720, blastp match of 29% identity and 3.2e-13 P-value to GP|12848653|dbj|BAB28039.1|....
490	AT5G01850	N	12.59	4.43	0	12.59	0	4.43	protein kinase, putative; FUNCTIONS IN: protein serine/threonine/tyrosine kinase activity, kinase activity; INVOLVED IN: prot....

491	AT5G02120	N	8.91	0	0	0	8.91	6.15	Encodes a one helix protein homologous to cyanobacterial high-light inducible proteins. The protein is localized to the thyl....
492	AT5G02150	N	8.91	0	0	6.15	0	8.91	binding; FUNCTIONS IN: binding; LOCATED IN: cellular_component unknown; EXPRESSED IN: 22 plant structures; EXPRESSED DURING: ....
	AT5G02160		8.91	8.91	0	0	6.15	0	unknown protein; FUNCTIONS IN: molecular_function unknown; INVOLVED IN: biological_process unknown; LOCATED IN: chloroplast t....
	AT5G02170		8.91	8.91	6.15	0	0	0	amino acid transporter family protein; FUNCTIONS IN: amino acid transmembrane transporter activity; INVOLVED
493	AT5G02440	Y	9.69	5.57	0	0	9.69	0	unknown protein; FUNCTIONS IN: molecular_function unknown; INVOLVED IN: biological_process unknown; LOCATED IN: cellular_comp
	AT5G02450	Y	9.69	5.57	0	9.69	0	0	60S ribosomal protein L36 (RPL36C); FUNCTIONS IN: structural constituent of ribosome; INVOLVED IN: translation; LOCATED IN: c....
494	AT5G03350		9.55	0	9.55	0	0	0	legume lectin family protein; FUNCTIONS IN: carbohydrate binding, sugar binding; INVOLVED IN: biological_process unknown; LOC
	AT5G03360	N	9.55	0	0	9.55	0	8.14	DC1 domain-containing protein; LOCATED IN: cellular_component unknown; EXPRESSED IN: inflorescence meristem, hypocotyl, root,....
495	AT5G03380	Y	8.14	0	0	0	8.14	0	heavy-metal-associated domain-containing protein; FUNCTIONS IN: metal ion binding; INVOLVED IN: metal ion transport; EXPRESSE
496	AT5G03700	Y	14.22	4.43	0	0	14.22	0	PAN domain-containing protein; FUNCTIONS IN: sugar binding; LOCATED IN: plasma membrane;

									EXPRESSED IN: stem, sperm cell, hypo....
497	AT5G03740		8.14	4.43	0	0	6.81	8.14	HD2-type histone deacetylase HDAC. Involved in the ABA and stress responses. Mediates transcriptional repression
	AT5G03745		8.14	0	0	6.81	0	8.14	pre-tRNA; tRNA-Leu (anticodon: TAA)
	AT5G03750		8.14	0	0	6.81	0	8.14	FUNCTIONS IN: molecular_function unknown; INVOLVED IN: biological_process unknown; LOCATED IN: cellular_component unknown; BE....
	AT5G03760	Y	8.14	0	0	0	8.14	6.81	encodes a beta-mannan synthase that is required for agrobacterium-mediated plant genetic transformation involves
498	AT5G05430		5.85	0	5.85	0	0	0	FUNCTIONS IN: molecular_function unknown; INVOLVED IN: biological_process unknown; LOCATED IN: cellular_component unknown; EX
	AT5G05435	Y	5.85	0	0	0	0	5.85	Potential natural antisense gene, locus overlaps with AT5G05430
	AT5G05440		5.85	0	0	5.85	0	0	unknown protein; FUNCTIONS IN: molecular_function unknown; LOCATED IN: cellular_component unknown; EXPRESSED IN: 22 plant str....
499	AT5G05800	Y	12.59	0	0	12.59	0	0	unknown protein; FUNCTIONS IN: molecular_function unknown; INVOLVED IN: biological_process unknown; LOCATED
	AT5G05810		12.59	0	12.59	0	0	0	ATL43; FUNCTIONS IN: protein binding, zinc ion binding; LOCATED IN: endomembrane system; CONTAINS InterPro DOMAIN/s: Zinc fin....
500	AT5G06120		44.39	0	0	5.57	0	44.39	Ran-binding protein, putative; FUNCTIONS IN: protein transporter activity, binding; INVOLVED IN: intracellular protein transp....

	AT5G06130	Y	44.39	0	0	0	44.3 9	5.57	chaperone protein dnaJ-related; EXPRESSED IN: 22 plant structures;
501	AT5G06320	N	15.98	0	0	0	15.9 8	4.43	encodes a protein whose sequence is similar to tobacco hairpin-induced gene (HIN1) and Arabidopsis non-race specific disease ....
502	AT5G06970	N	7.3	3.4	0	7.3	0	0	FUNCTIONS IN: molecular_function unknown; INVOLVED IN: biological_process unknown; LOCATED IN: cellular_component unknown
503	AT5G06980	N	14.22	0	0	7.3	0	14.22	unknown protein; FUNCTIONS IN: molecular_function unknown; INVOLVED IN: biological_process unknown; LOCATED IN: cellular_comp....
504	AT5G07200	Y	6.47	0	0	0	6.47	4.43	encodes a gibberellin 20-oxidase
505	AT5G07280	N	9.55	0	0	9.55	0	3.4	EXTRA MICROSPOROCYTES, Basic leucine zipper (bZIP) transcription factor. Nuclear localization. Involved in light-regulated transcriptional activatio....
506	AT5G08075	N	21.36	21.36	0	9.12	0	0	pre-tRNA; tRNA-Ala (anticodon: CGC)
	AT5G08080	Y	21.36	0	0	9.12	0	21.36	member of SYP13 Gene Family
	AT5G08090		21.36	9.12	0	0	21.3 6	0	unknown protein; FUNCTIONS IN: molecular_function unknown; INVOLVED IN: biological_process unknown; LOCATED IN: cellular_comp....
507	AT5G08250	N	6.81	0	6.81	0	0	0	cytochrome P450 family protein; FUNCTIONS IN: electron carrier activity, monooxygenase activity, iron ion binding, oxygen bin....
	AT5G08260		6.81	0	0	0	6.81	0	serine carboxypeptidase-like 35 (scpl35); FUNCTIONS IN: serine-type carboxypeptidase activity; INVOLVED IN: proteolysis; LOCA....
508	AT5G08790	N	7.39	0	0	0	7.39	4.43	induced by wounding, belongs to a large family of putative transcriptional activators with NAC

									domain.
509	AT5G10090	Y	19.45	19.45	0	0	0	0	tetratricopeptide repeat (TPR)-containing protein; FUNCTIONS IN: binding; INVOLVED IN: biological_process unknown; LOCATED IN....
	AT5G10120	N	19.45	19.45	0	0	0	4.43	ethylene insensitive 3 family protein; FUNCTIONS IN: transcription factor activity; INVOLVED IN: regulation of transcription;....
510	AT5G10340	N	5.8	0	0	0	5.8	0	F-box protein-related / SLF-related; FUNCTIONS IN: molecular_function unknown; INVOLVED IN: biological_process unknown; LOCAT
511	AT5G10690		6.81	0	0	0	6.81	0	pentatricopeptide (PPR) repeat-containing protein / CBS domain-containing protein; INVOLVED IN: biological_process unknown; L....
	AT5G10695	N	6.81	0	0	6.81	0	0	unknown protein; INVOLVED IN: biological_process unknown; LOCATED IN: cellular_component unknown; EXPRESSED IN: 23 plant stru....
512	AT5G10840		6.7	6.7	0	0	5.57	0	endomembrane protein 70, putative; LOCATED IN: integral to membrane, Golgi apparatus, plasma membrane, plant-type cell wall; ....
	AT5G10850		6.7	0	0	5.57	0	6.7	transposable element gene; similar to nucleic acid binding / zinc ion binding [Arabidopsis thaliana] (TAIR:AT2G01050.1); simi....
	AT5G10860		6.7	0	6.7	0	0	0	CBS domain-containing protein; INVOLVED IN: response to salt stress; LOCATED IN: mitochondrion; EXPRESSED IN: 24 plant struct....
	AT5G10870	Y	6.7	0	0	0	6.7	5.57	Encodes chorismate mutase AtCM2.
	AT5G10880		6.7	5.57	0	6.7	0	0	tRNA synthetase-related / tRNA ligase-related; FUNCTIONS IN: proline-tRNA ligase activity, aminoacyl-tRNA ligase activity, nu....

513	AT5G10980	Y	15.78	15.78	0	6.15	0	0	histone H3; FUNCTIONS IN: DNA binding; INVOLVED IN: nucleosome assembly; LOCATED IN: nucleus, nucleosome; EXPRESSED IN: 25 pl....
514	AT5G11420	Y	10.56	0	0	10.56	0	0	FUNCTIONS IN: molecular_function unknown; INVOLVED IN: biological_process unknown; LOCATED IN: cell wall, plant-type cell wal....
515	AT5G11740	N	7.75	0	6.81	0	5.57	7.75	Encodes arabinogalactan protein (AGP15).
516	AT5G11750		9.55	6.81	7.75	0	0	9.55	ribosomal protein L19 family protein; FUNCTIONS IN: structural constituent of ribosome; INVOLVED IN: translation, ribosome bi....
	AT5G11790	N	9.55	0	0	9.55	0	0	Ndr family protein; FUNCTIONS IN: molecular_function unknown; INVOLVED IN: cell differentiation; LOCATED IN: cellular_compone
517	AT5G11900	N	6.81	0	0	6.81	0	0	eukaryotic translation initiation factor SUI1 family protein; FUNCTIONS IN: translation initiation factor activity; INVOLVED ....
518	AT5G11970		19.45	0	12.59	0	0	19.45	unknown protein; FUNCTIONS IN: molecular_function unknown; INVOLVED IN: biological_process unknown; LOCATED IN: plasma membra....
	AT5G11977	Y	19.45	19.45	12.59	0	0	0	Encodes a microRNA that targets several SPL family members, including SPL3,4, and 5. By regulating the expression of SPL3 (an....
519	AT5G12330	Y	6.81	0	6.81	0	0	0	LATERAL ROOT PRIMORDIUM 1, A member of SHI gene family. Arabidopsis thaliana has ten members that encode proteins with a RING finger-like zinc finger mo....
520	AT5G12980	N	8.71	0	0	8.71	0	0	rcd1-like cell differentiation protein, putative; FUNCTIONS IN: molecular_function unknown; INVOLVED IN: multicellular organi....

521	AT5G13080		15.29	15.29	0	0	0	0	WRKY75 is one of several transcription factors induced during Pi deprivation. It is nuclear localized and regulated different....
	AT5G13090	Y	15.29	3.52	15.29	0	0	0	unknown protein; FUNCTIONS IN: molecular_function unknown; INVOLVED IN: biological_process unknown; EXPRESSED IN: 22 plant st....
	AT5G13100	Y	15.29	0	0	15.29	3.52	0	unknown protein;
522	AT5G13200	N	17.65	17.65	0	0	0	0	GRAM domain-containing protein / ABA-responsive protein-related; FUNCTIONS IN: molecular_function unknown; INVOLVED IN: biolo....
523	AT5G13230	N	9.55	0	9.55	0	0	0	pentatricopeptide (PPR) repeat-containing protein; INVOLVED IN: biological_process unknown; CONTAINS InterPro DOMAIN/s: Penta....
524	AT5G13340		11.03	0	0	11.03	0	0	unknown protein; FUNCTIONS IN: molecular_function unknown; INVOLVED IN: biological_process unknown; LOCATED IN: chloroplast; ....
	AT5G13350		11.03	0	0	11.03	0	0	auxin-responsive GH3 family protein; INVOLVED IN: response to auxin stimulus; LOCATED IN: cellular_component unknown; EXPRESS....
	AT5G13730		11.03	0	11.03	0	0	5.57	Encodes sigma 4 factor, involved in regulating the activity of the plastid-encoded RNA polymerase PEP. Regulates the overall....
	AT5G13740	Y	11.03	5.57	0	0	11.03	3.4	Encodes ZIF1 (ZINC-INDUCED FACILITATOR1), a member of the Major Facilitator Superfamily (MFS) of membrane proteins which are ....
525	AT5G13790	Y	12.59	0	0	12.59	0	3.4	AGL15 (AGAMOUS-Like 15) is a member of the MADS domain family of regulatory factors. Although AGL15 is preferentially express....

526	AT5G14260	N	6.98	0	0	0	6.98	0	SET domain-containing protein; FUNCTIONS IN: molecular_function unknown; INVOLVED IN: biological_process unknown; LOCATED
527	AT5G14640	Y	6.43	0	0	6.43	0	0	SHAGGY-LIKE KINASE 13 (SK13); FUNCTIONS IN: protein serine/threonine kinase activity, protein kinase activity, ATP binding; I....
528	AT5G15340	N	14.22	0	0	14.22	0	0	pentatricopeptide (PPR) repeat-containing protein; INVOLVED IN: biological_process unknown; LOCATED IN: mitochondrion; CONTAI....
529	AT5G15845		8.14	0	0	4.43	8.14	0	Potential natural antisense gene, locus overlaps with AT5G15850
	AT5G15850	Y	8.14	0	0	0	8.14	4.43	Homologous to the flowering-time gene CONSTANS
	AT5G15853		8.14	4.43	0	8.14	0	0	unknown protein; LOCATED IN: endomembrane system; Has 0 Blast hits to 0 proteins in 0 species (source: NCBI BLink).
530	AT5G16010	N	9.55	0	0	9.55	0	0	3-oxo-5-alpha-steroid 4-dehydrogenase family protein / steroid 5-alpha-reductase family protein; FUNCTIONS IN: oxidoreductase....
	AT5G16020		9.55	0	9.55	0	0	0	Encodes GEX3, a plasma membrane localized protein expressed in the male gametophyte. Required for micropylar pollen tube gui....
531	AT5G17230	Y	17.65	0	17.65	0	0	0	Encodes phytoene synthase.
	AT5G17233		17.65	0	0	17.65	0	0	unknown protein; Has 0 Blast hits to 0 proteins in 0 species (source: NCBI BLink).
532	AT5G18050		11.24	11.24	0	0	0	0	auxin-responsive protein, putative; FUNCTIONS IN: molecular_function unknown; INVOLVED IN: response to auxin stimulus; CONTAI
	AT5G18065		11.24	0	0	8.14	0	11.24	unknown protein; FUNCTIONS IN: molecular_function unknown; INVOLVED IN: biological_process unknown; LOCATED IN: cellular_comp....

	AT5G18080	N	11.24	8.14	0	11.24	3.4	0	auxin-responsive protein, putative; FUNCTIONS IN: molecular_function unknown; INVOLVED IN: response to auxin stimulus; CONTAI....
	AT5G18085		11.24	8.14	11.24	3.4	0	0	pre-tRNA; tRNA-Trp (anticodon: CCA)
533	AT5G19090	Y	11.03	0	0	0	11.0 3	9.55	heavy-metal-associated domain-containing protein; FUNCTIONS IN: metal ion binding; INVOLVED IN: metal ion transport; LOCATED ..
534	AT5G19340	Y	5.85	0	0	0	5.85	0	unknown protein; FUNCTIONS IN: molecular_function unknown; INVOLVED IN: biological_process unknown; LOCATED IN: cellular_comp
535	AT5G19890	N	8.1	0	0	0	8.1	0	peroxidase, putative; FUNCTIONS IN: electron carrier activity, peroxidase activity, heme binding; INVOLVED IN: response to ox....
536	AT5G20170	N	6.35	0	6.35	0	0	0	unknown protein; FUNCTIONS IN: molecular_function unknown; INVOLVED IN: biological_process unknown; LOCATED
	AT5G20180	Y	6.35	0	0	0	0	6.35	ribosomal protein L36 family protein; FUNCTIONS IN: structural constituent of ribosome; INVOLVED IN: translation, ribosome bi....
	AT5G20190	Y	6.35	0	6.35	0	0	0	binding; FUNCTIONS IN: binding; INVOLVED IN: biological_process unknown; LOCATED IN: chloroplast; CONTAINS InterPro DOMAIN/s:....
537	AT5G20225		8.78	0	8.78	0	0	0	Potential natural antisense gene, locus overlaps with AT5G20220
	AT5G20230		8.78	0	0	0	8.78	3.65	SENESCENCE-ASSOCIATED GENE 14, Al-stress-induced gene
	AT5G20240	N	8.78	0	8.78	0	0	3.65	PISTILLATA, Floral homeotic gene encoding a MADS domain transcription factor. Required for the specification of petal and stamen identiti....

538	AT5G20280	Y	6.81	0	6.81	0	0	3.65	Encodes a protein with putative sucrose-phosphate synthase activity. When the gene was expressed in transgenic tobacco plants....
539	AT5G20850	N	14.22	0	0	0	14.22	0	Encodes a homolog of yeast RAD51. Its mRNA is most abundant in early flower buds and is expressed at high levels in exponent....
540	AT5G21160	N	8.14	0	8.14	0	0	0	La domain-containing protein / proline-rich family protein; FUNCTIONS IN: molecular_function unknown; INVOLVED IN: biological....
	AT5G21170		8.14	0	0	0	8.14	0	Encodes AKINbeta1, a subunit of the SnRK1 kinase (Sucrose non-fermenting-1-related protein kinase). Involved in regulation o....
541	AT5G21280	Y	82.62	0	0	82.62	0	7.97	hydroxyproline-rich glycoprotein family protein; similar to unnamed protein product [Vitis vinifera] (GB:CAO43916.1)
542	AT5G21482	Y	50.92	5.85	0	0	50.92	0	CKX7, This gene used to be called AtCKX5. It encodes a protein whose sequence is similar to cytokinin oxidase/dehydrogenase, which ....
543	AT5G21940	Y	11.03	7.97	11.03	0	6.81	0	unknown protein; FUNCTIONS IN: molecular_function unknown; INVOLVED IN: biological_process unknown; BEST Arabidopsis thaliana....
544	AT5G21960	N	82.62	82.62	0	0	7.97	11.03	encodes a member of the DREB subfamily A-5 of ERF/AP2 transcription factor family. The protein contains one AP2 domain. There
545	AT5G22880	N	8.14	0	0	8.14	0	0	Encodes a histone 2B (H2B) protein. This protein can be ubiquitinated in planta, and this modification depends on the HUB1 an....
546	AT5G23020	Y	40.02	0	0	0	6.81	40.02	methylthioalkymalate synthase-like. Also known as 2-isopropylmalate synthase (IMS2). encodes a methylthioalkylmalate synthas....
	AT5G23030		40.02	40.02	0	0	0	0	Member of TETRASPANIN family

547	AT5G23260	N	27.16	27.16	0	0	0	0	AGL32, Arabidopsis Bsister, TT16
548	AT5G23730	Y	6.81	0	0	6.81	0	0	nucleotide binding; FUNCTIONS IN: nucleotide binding; LOCATED IN: CUL4 RING ubiquitin ligase complex, heterotrimeric G-protei....
549	AT5G23810	N	6.15	0	0	0	6.15	4.43	Encodes nonfunctional amino acid transporter. AAP7 is the most distantly related member of the AAP family, a group of well c....
550	AT5G24030	N	6.81	0	0	0	6.81	0	Encodes a protein with ten predicted transmembrane helices. The SLAH3 protein has similarity to the SLAC1 protein involved in....
551	AT5G24780	Y	12.13	0	0	0	12.13	5.6	VEGETATIVE STORAGE PROTEIN 1, encodes an acid phosphatase similar to soybean vegetative storage proteins. Gene expression is induced by wounding and jasmon....
552	AT5G25290	Y	12.59	0	0	12.59	0	0	F-box family protein; FUNCTIONS IN: molecular_function unknown; INVOLVED IN: biological_process unknown; LOCATED IN: cellular....
554	AT5G25610	Y	32.39	32.39	0	0	3.4	0	responsive to dehydration 22 (RD22) mediated by ABA
553	AT5G25830	Y	6.81	0	0	6.81	0	0	Encodes a member of the GATA factor family of zinc finger transcription factors
555	AT5G26940	Y	7.3	0	0	7.3	0	0	exonuclease family protein; FUNCTIONS IN: exonuclease activity, nucleic acid binding; LOCATED IN: intracellular, chloroplast;....
556	AT5G28770	Y	12.59	0	0	0	12.59	0	bZIP protein BZO2H3 mRNA, partial cds
557	AT5G29602	N	8.71	0	0	0	8.71	0	transposable element gene; similar to unknown protein [Arabidopsis thaliana] (TAIR:AT2G07090.1)

558	AT5G37050		5.62	0	5.62	0	0	0	FUNCTIONS IN: molecular_function unknown; INVOLVED IN: biological_process unknown; LOCATED IN: chloroplast; BEST Arabidopsis ....
	AT5G37055	N	5.62	0	0	5.62	0	0	Encodes SERRATED LEAVES AND EARLY FLOWERING (SEF), an Arabidopsis homolog of the yeast SWC6 protein, a conserved subunit of t....
	AT5G37060		5.62	0	0	0	5.62	0	member of Putative Na+/H+ antiporter family
559	AT5G38700	N	9.55	0	9.55	0	0	0	unknown protein; FUNCTIONS IN: molecular_function unknown; INVOLVED IN: biological_process unknown; LOCATED IN: mitochondrion....
560	AT5G40340	Y	7	0	0	0	7	0	PWWP domain-containing protein; FUNCTIONS IN: molecular_function unknown; LOCATED IN: nucleolus; EXPRESSED IN: 24 plant struc....
561	AT5G40720	Y	6.81	0	0	0	6.81	0	FUNCTIONS IN: molecular_function unknown; INVOLVED IN: biological_process unknown; LOCATED IN: cellular_component unknown; CO....
	AT5G40730		6.81	0	6.81	0	0	0	Encodes an arabinogalactan-protein (AGP24).
562	AT5G42650	Y	8.71	0	0	0	8.71	0	DELAYED DEHISCENCE 2, Encodes a member of the cytochrome p450 CYP74 gene family that functions as an allene oxide synthase. This enzyme catalyzes
563	AT5G43270	Y	12.59	0	0	0	12.5 9	0	SPL2, SQUAMOSA PROMOTER BINDING PROTEIN-LIKE2
564	AT5G43700	Y	105.57	0	0	0	105. 57	0	IAA4, Auxin inducible protein similar to transcription factors.
565	AT5G44590	Y	42.29	0	0	42.29	0	0	similar to unknown protein [Arabidopsis thaliana] (TAIR:AT5G44600.1); similar to expressed protein [Oryza sativa (japonica cu....

566	AT5G45050	N	6.81	0	0	6.81	0	0	Encodes a member of WRKY Transcription Factor (Group II-e) that confers resistance to tobacco ringspot nepovirus.
	AT5G45060		6.81	0	0	6.81	0	0	disease resistance protein (TIR-NBS-LRR class), putative; FUNCTIONS IN: transmembrane receptor activity, protein binding, nuc....
567	AT5G45980	N	6.47	0	0	0	6.47	0	WOX8, Arabidopsis thaliana WOX8 protein. Contains similarity to homeodomain transcription factor. Positively regulates early embry....
	AT5G45990		6.47	0	6.47	0	0	0	crooked neck protein, putative / cell cycle protein, putative; FUNCTIONS IN: binding; INVOLVED IN: RNA processing; LOCATED IN....
568	AT5G47180	Y	6.81	5.57	0	6.81	0	0	vesicle-associated membrane family protein / VAMP family protein; FUNCTIONS IN: structural molecule activity; LOCATED IN: pla....
569	AT5G47430	N	8.14	3.97	0	0	8.14	0	zinc ion binding; FUNCTIONS IN: zinc ion binding; INVOLVED IN: biological_process unknown; LOCATED IN: nucleus; EXPRESSED IN:....
570	AT5G47635		7.83	0	0	7.83	0	0	unknown protein
	AT5G47640	Y	7.83	0	0	7.83	0	0	NUCLEAR FACTOR Y, SUBUNIT B2 (NF-YB2); FUNCTIONS IN: transcription factor activity;
571	AT5G49760	N	6.81	0	0	0	6.81	0	leucine-rich repeat family protein / protein kinase family protein; FUNCTIONS IN: kinase activity; INVOLVED IN: protein amino....
572	AT5G51110	N	9.55	0	0	9.55	0	3.19	4-alpha-hydroxytetrahydrobiopterin dehydratase; FUNCTIONS IN: 4-alpha-hydroxytetrahydrobiopterin dehydratase activity; INVOLV....
573	AT5G51120	Y	9.55	3.19	0	9.55	0	0	Encodes a homolog of the protein PABN1, a polyadenylation factor subunit.

574	AT5G51170		8.14	0	0	0	8.14	0	unknown protein; FUNCTIONS IN: molecular_function unknown; INVOLVED IN: biological_process unknown; LOCATED IN: cellular_comp....
	AT5G51174	N	8.14	0	0	8.14	0	0	Encodes a C/D box snoRNA (snoR30). Gb: AJ505639
	AT5G51180		8.14	0	8.14	0	0	0	unknown protein; INVOLVED IN: biological_process unknown; LOCATED IN: cellular_component unknown; EXPRESSED
575	AT5G51900		9.12	0	0	9.12	0	0	cytochrome P450 family; FUNCTIONS IN: electron carrier activity, monooxygenase activity, iron ion binding, oxygen binding, he....
	AT5G51910	Y	9.12	0	0	0	9.12	0	TCP family transcription factor, putative; FUNCTIONS IN: transcription factor activity; INVOLVED IN: regulation of transcript....
576	AT5G52050	N	8.78	0	0	0	8.78	0	MATE efflux protein-related; FUNCTIONS IN: drug transporter activity, antiporter activity; INVOLVED IN: multidrug transport; ....
577	AT5G52660	Y	19.45	0	0	19.45	0	5.52	myb family transcription factor; similar to myb family transcription factor [Arabidopsis thaliana] (TAIR:AT4G01280.1); simila....
	AT5G52670		19.45	0	19.45	0	0	0	heavy-metal-associated domain-containing protein; FUNCTIONS IN: metal ion binding; INVOLVED IN: metal ion transport; LOCATED ....
578	AT5G52950		9.12	0	0	9.12	0	0	unknown protein; FUNCTIONS IN: molecular_function unknown; INVOLVED IN: biological_process unknown; LOCATED IN: cellular_comp....
	AT5G52960	Y	9.12	0	0	9.12	0	0	unknown protein; FUNCTIONS IN: molecular_function unknown; INVOLVED IN: biological_process unknown; LOCATED IN: chloroplast; ....

	AT5G52965		9.12	0	9.12	0	0	0	unknown protein; FUNCTIONS IN: molecular_function unknown; INVOLVED IN: biological_process unknown; LOCATED IN: endomembrane ....
579	AT5G53370	Y	8.14	0	0	0	8.14	0	PECTIN METHYLESTERASE PCR FRAGMENT F (PMEPCRF); FUNCTIONS IN: pectinesterase activity; INVOLVED IN: cell wall modification; L....
580	AT5G53460	N	8.14	3.4	0	8.14	3.95	0	NADH-dependent glutamate synthase
581	AT5G53905		8.71	0	0	0	8.71	0	unknown protein; FUNCTIONS IN: molecular_function unknown; INVOLVED IN: biological_process unknown; LOCATED IN: cellular_comp....
	AT5G53910		8.71	0	8.71	0	0	0	(S)-2-hydroxy-acid oxidase, peroxisomal, putative / glycolate oxidase, putative / short chain alpha-hydroxy acid oxidase, put....
582	AT5G55120	N	6.81	0	0	6.81	0	0	Encodes a GDP-L-galactose phosphorylase, with similar biochemical properties as VTC2
583	AT5G55180	N	14.22	0	0	14.22	4.69	0	glycosyl hydrolase family 17 protein; similar to hydrolase, hydrolyzing O-glycosyl compounds [Arabidopsis thaliana] (TAIR:AT4....
584	AT5G56980	Y	59.09	0	0	0	59.0 9	0	unknown protein
585	AT5G57330	Y	8.14	0	0	0	8.14	0	aldose 1-epimerase family protein; FUNCTIONS IN: isomerase activity, carbohydrate binding, aldose 1-epimerase activity, catal....
586	AT5G57550	Y	10.68	10.68	0	7.75	0	3.4	xyloglucan endotransglycosylase-related protein (XTR3)
	AT5G57560		10.68	10.68	0	0	7.75	0	Encodes a cell wall-modifying enzyme, rapidly upregulated in response to environmental stimuli

	AT5G57567		10.68	3.4	10.68	5.12	0	7.75		unknown protein; FUNCTIONS IN: molecular_function unknown; INVOLVED IN: biological_process unknown; LOCATED IN: endomembrane ....
	AT5G57570	Y	10.68	3.4	0	0	10.6<br>8	7.75		FUNCTIONS IN: molecular_function unknown; INVOLVED IN: N-terminal protein myristoylation; LOCATED IN: cellular_component unkn....
587	AT5G58412	Y	33.42	0	0	0	33.4<br>2	3.45		Encodes a Plant thionin family protein
	AT5G58420		33.42	0	33.42	0	0	3.45		40S ribosomal protein S4 (RPS4D); FUNCTIONS IN: structural constituent of ribosome; INVOLVED IN: translation; LOCATED IN: cyt....
588	AT5G58550		5.85	3.4	5.85	0	0	0		Encodes a paralog of ETO1, which is a negative regulator of ACS5 (a key enzyme in ethylene biosynthesis pathway). EOL2 also i....
	AT5G58560	Y	5.85	0	0	0	5.85	3.4		phosphatidate cytidylyltransferase family protein; FUNCTIONS IN: phosphatidate cytidylyltransferase activity, transferase act....
589	AT5G58784		6.81	0	6.81	0	0	0		dehydrodolichyl diphosphate synthase; FUNCTIONS IN: dehydrodolichyl diphosphate synthase activity; INVOLVED IN: dolichol bios....
	AT5G58787	N	6.81	0	0	6.81	0	0		zinc finger (C3HC4-type RING finger) family protein; FUNCTIONS IN: protein binding, zinc ion binding; INVOLVED IN: biological....
590	AT5G58900	Y	33.42	0	0	0	33.4<br>2	0		myb family transcription factor; FUNCTIONS IN: transcription factor activity, DNA binding; INVOLVED IN: regulation of transcr....
591	AT5G59130		8.14	0	0	0	8.14	0		subtilase family protein; FUNCTIONS IN: identical protein binding, serine-type endopeptidase activity; INVOLVED IN: proteolys....
592	AT5G59530		17.83	3.97	17.83	0	0	4.43		2-oxoglutarate-dependent dioxygenase, putative; FUNCTIONS IN: transferase activity, transferring

										glycosyl groups; EXPRESSED I....
	AT5G59540	Y	17.83	0	3.97	17.83	0	4.43		oxidoreductase, 2OG-Fe(II) oxygenase family protein; similar to 2-oxoglutarate-dependent dioxygenase, putative [Arabidopsis t....
593	AT5G59730	Y	8.14	7.39	8.14	0	6.47	0		A member of EXO70 gene family, putative exocyst subunits, conserved in land plants. Arabidopsis thaliana contains 23 putative....
	AT5G59732		8.14	0	0	6.47	0	8.14		Potential natural antisense gene, locus overlaps with AT5G59730
	AT5G59740	Y	8.14	0	0	7.39	0	8.14		UDP-galactose/UDP-glucose transporter-related; FUNCTIONS IN: galactose transmembrane transporter activity; EXPRESSED IN: 23 p
	AT5G59750		8.14	8.14	0	7.39	0	0		riboflavin biosynthesis protein, putative; FUNCTIONS IN: 3,4-dihydroxy-2-butanone-4-phosphate synthase activity, GTP cyclohyd....
594	AT5G60580	Y	12.59	0	0	0	12.5 9	0		zinc finger (C3HC4-type RING finger) family protein; FUNCTIONS IN: protein binding, zinc ion binding; INVOLVED IN: biological....
	AT5G60590		12.59	0	0	12.59	0	0		yrdC protein-related; FUNCTIONS IN: molecular_function unknown; INVOLVED IN: biological_process unknown; LOCATED IN: chloropl....
595	AT5G60910	Y	16.36	4.43	14.2	16.36	6.47	5.57		FRUITFULL
596	AT5G61970	Y	9.55	0	9.55	0	0	0		signal recognition particle-related / SRP-related; INVOLVED IN: biological_process unknown; LOCATED IN: cellular_component un....
597	AT5G62000	Y	5.85	4.43	0	5.85	3.4	0		ARF2, Encodes an auxin response factor. Mutants have many defects including enlarged rosette leaves, reduced fertility, later senes....

598	AT5G62480	N	7.75	3.4	7.75	0	0	0	Encodes glutathione transferase belonging to the tau class of GSTs. Naming convention according to Wagner et al. (2002).
599	AT5G62490	N	15.32	15.32	0	0	7.75	3.4	Part of the AtHVA22 family. Protein expression is ABA- and stress-inducible.
	AT5G62500		15.32	15.32	7.75	3.4	0	3.4	encodes a homolog of animal microtubule-end-binding protein. There are two other members of this family. EB1 forms foci at re....
600	AT5G62880	Y	11.03	0	11.03	0	0	0	A member of ROP GTPase gene family
601	AT5G64310		14.16	0	0	0	14.16	0	Encodes arabinogalactan-protein (AGP1).
	AT5G64330	Y	14.16	14.16	0	0	0	0	Involved in blue light response signaling pathway; interacts with the blue light photoreceptor NPH1.Null mutations abolish ph....
602	AT5G64410	Y	6.81	0	0	0	6.81	0	oligopeptide transporter
	AT5G64420		6.81	6.81	0	0	0	0	DNA polymerase V family; FUNCTIONS IN: DNA-directed DNA polymerase activity, DNA binding; INVOLVED IN: DNA replication, trans....
	AT5G64430		6.81	0	0	0	0	6.81	octicosapeptide/Phox/Bem1p (PB1) domain-containing protein; FUNCTIONS IN: molecular_function unknown; INVOLVED IN: biological....
603	AT5G64850	Y	20.07	0	0	0	0	20.07	unknown protein; FUNCTIONS IN: molecular_function unknown; INVOLVED IN: biological_process unknown; LOCATED IN: plasma membra....
	AT5G64855		20.07	0	0	0	0	20.07	pre-tRNA; tRNA-Leu (anticodon: AAG)
	AT5G64860		20.07	0	20.07	0	0	0	Encodes a maltotriose-metabolizing enzyme with chloroplastic a-1,4-glucanotransferase activity. Mutant has altered starc....

	AT5G64870	Y	20.07	0	0	0	20.0<br>7	0	FUNCTIONS IN: molecular_function unknown; INVOLVED IN: biological_process unknown; LOCATED IN: plasma membrane, vacuole; EXPR....
604	AT5G65160		8.14	6.81	0	0	8.14	0	tetratricopeptide repeat (TPR)-containing protein; FUNCTIONS IN: binding; INVOLVED IN: biological_process unknown; LOCATED IN....
	AT5G65165	N	8.14	0	0	6.81	0	8.14	One of three isoforms of the iron-sulfur component of the succinate dehydrogenase complex, a component of the mitochondrial r
605	AT5G65630	Y	8.14	0	3.4	8.14	3.52	0	This gene is predicted to encode a bromodomain-containing protein. Plant lines expressing RNAi constructs targeted against GT....
606	AT5G66270	Y	8.14	0	0	0	8.14	0	zinc finger (CCCH-type) family protein; FUNCTIONS IN: zinc ion binding, nucleic acid binding; INVOLVED IN: biological_process....
	AT5G66280		8.14	0	8.14	0	0	0	GDP-D-mannose 4,6-dehydratase
607	AT5G66380		14.22	14.22	0	6.81	0	0	Encodes a folate transporter that is located in the chloroplast envelope and is able to mediate exogenous folate uptake when ....
	AT5G66390		14.22	14.22	0	0	6.81	0	peroxidase 72 (PER72) (P72) (PRXR8); FUNCTIONS IN: electron carrier activity, peroxidase activity, heme binding; INVOLVED IN:....
	AT5G66400	N	14.22	4.43	0	14.22	0	6.81	RESPONSIVE TO ABA18, Belongs to the dehydrin protein family, which contains highly conserved stretches of 7-17 residues that are repetitively scat....
	AT5G66410		14.22	6.81	0	0	14.2<br>2	4.43	Encodes a protein that functions in microtubule assembly. Plants with reduced levels of both PLP3a (At3g50960) and PLP3b show....

608	AT5G66460	N	6.81	0	0	6.81	0	0	(1-4)-beta-mannan endohydrolase, putative; FUNCTIONS IN: cation binding, hydrolase activity, hydrolyzing O-glycosyl compounds....
609	AT5G66558		6.75	0	0	0	6.75	0	Potential natural antisense gene, locus overlaps with AT5G66560
	AT5G66560	Y	6.75	0	0	0	6.75	0	phototropic-responsive NPH3 family protein; FUNCTIONS IN: protein binding, signal transducer activity; INVOLVED IN: response ....
610	AT5G66610	Y	6.81	0	0	6.81	0	0	DA1-RELATED PROTEIN 7 (DAR7); FUNCTIONS IN: zinc ion binding; LOCATED IN: cellular_component unknown; EXPRESSED IN: 16 plant ....
611	AT5G66658		8.14	0	0	0	0	8.14	unknown protein; FUNCTIONS IN: molecular_function unknown; INVOLVED IN: biological_process unknown; LOCATED IN: mitochondrion....
	AT5G66675	N	8.14	0	0	8.14	0	0	unknown protein; FUNCTIONS IN: molecular_function unknown; INVOLVED IN: biological_process unknown; LOCATED IN: cellular_comp....
612	AT5G66730	Y	12.59	0	0	12.59	0	3.4	zinc finger (C2H2 type) family protein; FUNCTIONS IN: transcription factor activity, zinc ion binding, nucleic acid binding; ....
	AT5G66740		12.59	3.4	12.59	0	0	0	unknown protein; FUNCTIONS IN: molecular_function unknown; INVOLVED IN: biological_process unknown; LOCATED IN: cellular_comp....
613	AT5G66910		8.91	4.05	8.91	0	0	0	disease resistance protein (CC-NBS-LRR class), putative; FUNCTIONS IN: protein binding, ATP binding; INVOLVED IN: defense res....
	AT5G66920	Y	8.91	0	0	0	8.91	4.05	SKU5 Similar 17 (sks17); FUNCTIONS IN: oxidoreductase activity, copper ion binding;

									LOCATED IN: cell wall, plant-type cell wa....
614	AT5G67180	Y	73.71	0	73.71	0	0	3.3	AP2 domain-containing transcription factor, putative; Identical to AP2-like ethylene-responsive transcription factor TARGET O....
615	AT5G67290	Y	8.14	8.14	6.15	4.43	0	4.5	FAD-dependent oxidoreductase family protein; FUNCTIONS IN: oxidoreductase activity; INVOLVED IN: biological_process unknown; ....
	AT5G67300	Y	8.14	6.15	0	8.14	4.43	0	Member of the R2R3 factor MYB gene family involved in mediating plant responses to a variety of abiotic stimiuli.
616	AT5G67370	N	19.45	0	0	19.45	0	0	unknown protein; FUNCTIONS IN: molecular_function unknown; INVOLVED IN: biological_process unknown; EXPRESSED IN: 21 plant st....
Peak	Locus	SEP3 target	max3kb1kb	u3000	u2000	u1000	d0	d1000	

Table S2. Primer list

Primer name	Primer number	Sequence 5'- 3'
AttB site addition For	PDS 7387	GGGGACAAGTTTGTACAAAAAAGCAGGCT
AttB site addition Rev	PDS 7388	GGGGACCACTTTGTACAAGAAAGCTGGGT
BP SAUR10 CDS For	none	AAAAAGCAGGCTATGGCAATAAAGAGATCGAG
BP SAUR10 CDS Rev	none	AGAAAGCTGGGTTATCTAAACATGGAGATAAGAG
ful-7 genotyping For	none	ATCAAATCACAATTAGGTACATCG
ful-7 genotyping Rev	none	GACATACTGTAGTTAATTATTTCAAC
pROK LB primer	PDS 8611	GAACAACACTCAACCCTATCTCG
pSAUR10 BP for	none	AAAAAGCAGGCTCTCTCAGACTTGAGCATC
pSAUR10 BP rev	none	AGAAAGCTGGGTGAAGAATGTTATATGGAGG

pFUL BP for	none	AAAAAGCAGGCTACATCATCTGTATTTGTTT
pFUL BP rev	none	AGAAAGCTGGGTATCTCTCTCTCTTCAAAA
QUBC21 For	PDS 7341	ATGCTTGGAGTCCTGCTTGG
QUBC21 Rev	PDS 7342	TGCCATTGAATTGAACCCTCTC
QTIP41 For	PDS 2989	GTGAAAACTGTTGGAGAGAAGCAA
QTIP41 Rev	PDS 2990	TCAACTGGATACCCTTTCGCA
QSAUR16 For	PDS 7492	AACAATGCTACGACGAGGAAG
QSAUR16 Rev	PDS 7493	ATTCAGGATGAGTCAAGAAGGAG
QSAUR12 For	PDS 7494	AATGCTATGATGAGGATGGTCTG
QSAUR12 Rev	PDS 7495	CTTAGGATGAGTCAAGAAGGATATG
QSAUR8 For	PDS 7185	GAAGAATGGAGGAGGAGGATATG
QSAUR8 Rev	PDS 7186	TGTATCTGCTTCTGTTGTGTCC
QSAUR50 For	PDS 7187	AAGATTGCCTTCCGCTTGAC
QSAUR50 Rev	PDS 7188	AATGTATCTGCTTCTGTTCTCTCC
QSAUR54 For	PDS 7189	AAAGAAACAGAGCAGTGAGTATAAC
QSAUR54 Rev	PDS 7190	TGGGAACATCCAGAGGAAGAG
QSAUR51 For	PDS 7191	CGAAAACGGATCTCCTCTTAAC
QSAUR51 Rev	PDS 7192	GTACCTAACCCGATTCTCTCC
QSAUR10 For	PDS 7193	CGAGTCTAAGAAAGATGAAGAACG
QSAUR10 Rev	PDS 7194	GTGATGGAGCCATGAGATAGG
QSAUR9 For	PDS 7195	CAAGGTAATTGCTACTTCAACGAC
QSAUR9 Rev	PDS 7196	CACGAGATTGGGACCACATAG
LBb3.1 SALK T-DNAl ines	PDS 5156	ATTTTGCCGATTTCGGAAC
LB primer SM lines	PDS 5384	CGTGAAGTTTCTCATCTAAGCCCC
SM 3_1724 genotyping Rev	PDS 8531	ATGGAGATAAGAGACCTGAAG
FLAG_590D09 genotyping For	PDS 6631	ATCCCAATGCAAAATCACTCC
FLAG_590D09 genotyping Rev	PDS 6632	AACGGGTTAATGCCTACGAAG
Tag3 RB FLAG T-DNA	PDS 7259	CTGATACCAGACGTTGCCCGCATAA

SAUR10 EMSA F	PDS 9281	TTCTTTGTTGAGGGAAGTG
SAUR10 EMSA R	PDS 9282	AACCATTGTGTTTATTTTGC
SAUR10 EMSA frag1 rev	PDS 9773	TACCATCGTTGGACATTATTGAAACATACA
SAUR10 EMSA frag2 for	PDS 9774	AATGTCCAACGATGGTAACAAAATACAC
pGEM-T, fwd + 5'-Cy3	PDS 6024	CATGGCCGCGGGATT
pGEM-T, fwd + 5'-Cy5	PDS 6025	AATCACTAGTGCGGCCGC
ChIP REF1 For	PDS 9817	TCTCCGACCTTTCTTCACACCCATTCC
ChIP REF1 R	PDS 9949	CTGAGAACTTGCTTACTTGATAGACTC
ChIP REF2 For	PDS 9819	GCTATCCACAGGTTAGATAAAGGAG
ChIP REF2 R	PDS 9950	GGACTAGATTTGAGGAAAGGAAGGA
Q PIL1 F	PDS 9884	GGCTTCATTGTTGGATGAGGCT
Q PIL1 R	PDS 9885	GGAGAGTAATGACCCATTGGCA
Q RGL2 F	PDS 9886	TGGACCTCCGTCGTTTCGTC
Q RGL2 R	PDS 9887	CGGGTTCAAGATCCGATAAACTCTC
Q IBH F	PDS 9888	CGTGAGGAAGATCAAGAAGGCT
Q IBH R	PDS 9889	TGTCATCTTTGTCTGCTCTACGC
Q PAR1 F	PDS 9890	TAGCCACTCCCGACGCCACGA
Q PAR1 R	PDS 9891	CTGCTTCTTCTCGGTCTTCACG
Q PIF4 F	PDS 9892	AGGAGGAGAGATAGGATCAATGAG
Q PIF4 R	PDS 9893	CCACTCCCCATCCACATCAC
Q RGL1 F	PDS 9894	AACTTGGTCAGCTTGCGAGTAC
Q RGL1 R	PDS 9895	ACCGCCACTGATTCTAAACCG
Q BZR2 F	PDS 9896	GCTGGTTGGGTTGTTGAAGAAG
Q BZR2 R	PDS 9897	ATGGGAAGAGTAAGGAGTTGCTC
Q RGA2 F	PDS 9898	CGGAGATTTTCACTGTGGTTGAG
Q RGA2 R	PDS 9899	CGGTACACCTTCCAACGAGTC
Q BZR1 F	PDS 9900	GTGTTGAAGCTGGTTGGGTTGTTG
Q BZR1 R	PDS 9901	AAGAGGGCTCTGGTTCTGTGATG

Q FHY F	PDS 9902	GATGATTCGGATCTTGAAAGCATC
Q FHY R	PDS 9903	CACTCTTTCCGTTACTCTTCATC
Q PAR2 F	PDS 9904	CCTAGCCACTTCCCATACCAAAC
Q PAR2 R	PDS 9905	CGTCGGTCTCGCTTACACTCG
Q PIL2 F	PDS 9906	GATGACGAGTCAGATGATGCGAG
Q PIL2 R	PDS 9907	TGTGAGAGTTAGGTAGTAGATTTTGC
Q BIN2 F	PDS 9908	CGAAATCTTGTGCCTCTTCTTGG
Q BIN2 R	PDS 9909	GTGTGGACCAGTTTAGTTTCACC
ChIP SAUR10 F	PDS 9951	GTACCTCTATGTTTTTCTTTGTTGAGG
ChIP SAUR10 R	PDS 9952	GGAGATACATACATAAGGAGGACTC
ChIP PIL1 F	PDS 9953	CTGTAGTGAGTGACATGCGAGAGA
ChIP PIL1 R	PDS 9954	CCAAAAAAAGCCTAATTGTGGGTCC
ChIP RGL2 F	PDS 9955	GTGGGGATTGTATATAGGTATGGTC
ChIP RGL2 R	PDS 9956	CAAATAATGTAAACCACAAAAGCAAAACAGG
ChIP GAI F	PDS 9957	GATTTAAGAATAGTTGCCGTAAGCGA
ChIP GAI R	PDS 9958	ACTATTTTGTCAATTACATGTTACGATAGAG
ChIP BIN1 F	PDS 9959	GTGGGTGATTTGTCACACGAG
ChIP BIN1 R	PDS 9960	GTTTAGTGATGTATTAATTTCGTATAATAGAAC
ChIP PIF4 F	PDS 9961	TCCTAATCTGGTCAGTAGTACTCTT
ChIP PIF4 R	PDS 9962	GCAAGTCCATGAGTCCGTTCTC
ChIP PAR2 F	PZN 460	TGGTTGTGCGATTTAGGTTTGG
ChIP PAR2 R	PZN 461	ACAAACACTCCAACACATAATTCG
SAUR16 EMSA Frag F	PDS 9975	TTCCTAAAGTAAGCAGACAAGT
SAUR16 EMSA Frag R	PDS 9976	TACTCTTGCCTCCTTCTTCC
Q CKX5 F	PZN 46	GATAAAGGCGTTTTCAAGGGCAT
Q CKX5 R	PZN 47	CGGCGTCACGGCTGAGCT
Q CKX6 F	PZN 48	ACATACTAACGGATACAAGCAACGG
Q CKX6 R	PZN 49	CACCAGGTAGAATACCTCTTCCTC

Q CKX7 F	PZN 50	CAAGAACGGAGTCAATGGTCCA
Q CKX7 R	PZN 51	CAATGTAGAATATCTCTCCTTCTTCCG
ChIP CKX5 F	PZN 54	TAGTGAGCCGATGAATGTTTGAC
ChIP CKX5 R	PZN 55	GATATTATTGACATAATTTACTATGCTGCA
ChIP CKX6 F	PZN 56	CTTATATGAGCAGAGAGAGAAATATGC
ChIP CKX6 R	PZN 57	CTCTCGTTTTCTTTTTACAGTTTGGAAG
**EMSA probe sequences**		
pSAUR10 fragment		TTCTTTGTTGAGGGAAGTGGGCATTTATGTATGTTTCAATAATGTCCAA[illegible]
mSAUR10 fragment		TTCTTTGTTGAGGGAAGTGGGCATTTATGTATGTTTCAATAATGTCCAA[illegible]
pSAUR16 fragment		TTTCCTAAAGTAAGCAGACAAGTTTCAAGACTAGTAAAATCTGCATTA[illegible]
